# Supplementary material for: Priorities of patients with multimorbidity and of clinicians regarding treatment and health outcomes: a systematic mixed studies review
Source: BMJ Open. 2020 Feb 12;10(2):e033445. doi: 10.1136/bmjopen-2019-033445 (PMC7045037; doi:10.1136/bmjopen-2019-033445)
Supplement: Supplementary data [file bmjopen-2019-033445supp001.pdf]

**1. Patient*.mp.**
**2. Patients/**
**3. 1 or 2**
**4. Priorit*.mp. [mp=title, abstract, original title, name of substance word, subject heading word, keyword heading word, protocol supplementary concept word, rare disease supplementary concept word, unique identifier, synonyms]**
**5. Choice*.mp.**
**6. Preference*.mp.**
**7. Aim*.mp.**
**8. Goal*.mp.**
**9. 4 or 5 or 6 or 7 or 8**
**10. Doctor*.mp.**
**11. Physicians/**
**12. Clinician*.mp.**
**13. Primary Health Care/ or Physicians, Family/ or Family Practice/ or General Practitioners/**
**14. General practitioner*.mp.**
**15. 10 or 11 or 12 or 13 or 14**
**16. Multimorbidit*.mp. [mp=title, abstract, original title, name of substance word, subject heading word, keyword heading word, protocol supplementary concept word, rare disease supplementary concept word, unique identifier, synonyms]**
**17. Multi-morbidit*.mp. [mp=title, abstract, original title, name of substance word, subject heading word, keyword heading word, protocol supplementary concept word, rare disease supplementary concept word, unique identifier, synonyms]**
**18. Multiple morbidit*.mp. [mp=title, abstract, original title, name of substance word, subject heading word, keyword heading word, protocol supplementary concept word, rare disease supplementary concept word, unique identifier, synonyms]**
**19. 16 or 17 or 18**
**20. 3 and 9 and 15 and 19**
**21. Multi morbid*.mp. [mp=title, abstract, original title, name of substance word, subject heading word, keyword heading word, protocol supplementary concept word, rare disease supplementary concept word, unique identifier, synonyms]**
**22. 16 or 17 or 18 or 21**
**23. 3 and 9 and 15 and 22**

Appendix 1
