# Supplementary material for: Priorities of patients with multimorbidity and of clinicians regarding treatment and health outcomes: a systematic mixed studies review
Source: BMJ Open. 2020 Feb 12;10(2):e033445. doi: 10.1136/bmjopen-2019-033445 (PMC7045037; doi:10.1136/bmjopen-2019-033445)
Supplement: Supplementary data [file bmjopen-2019-033445supp002.pdf]

CASP checklist for qualitative research summary table

	**Kuluski et al** [1]	**Schoenberg et al** [2]	**Cheraghi-Sohi et al** [3]	**Naik et al** [4]	**Lindsay et al** [5]	**Hansen et al** [6]	**Morris et al** [7]	**Elliott et al** [8]	**Fried et al** [9]	**Turner et al** [10]	**Van Summeren et al** [11]	**Caughey et al** [12]
Was there a clear statement of the aims of the research?	YES	YES	YES	YES	YES	YES	YES	YES	YES	YES	YES	YES
Is a qualitative methodology appropriate?	YES	YES	YES	YES	YES	YES	YES	YES	YES	NO- Quantitative or mixed methods methodology would have been more appropriate as the aim was to rank factors, although data collected using a qualitative technique, it lacks richness and appears to be presented in a quantitative manner	YES	YES
Was the research design appropriate to the aims of the research?	YES	YES	YES	YES	YES	YES	YES	YES	YES	YES	YES	YES
Was the recruitment strategy	YES	YES	YES	YES	YES	YES	NO- no explanation given as to	YES	YES	YES	YES	YES

appropriate to the aims of the research							why the specific conditions were chosen (COPD, IBS etc)					
Were the data collected in a way that addressed the research issue?	YES	YES	YES	YES	YES	YES	YES	YES	YES	YES	YES	YES
Has the relationship between researcher and participants been adequately considered?	YES	YES	YES	NO- no information given on background of main researcher and no consideration given to possibility of researcher bias at any point.	NO- There is no background information given on the researcher (sole in this case) and there has been no evidence of any consideration of researcher bias at any point during the study.	NO- there has been no evidence of any consideration of researcher bias at any point during the study	NO- no information given on background of main researcher and no consideration given to possibility of researcher bias at any point. However there was some evidence of reflexivity during the data collection process when emerging areas of interest that could be incorporated into future interviews	NO- background of RAE who conducted interviews and main aspect of analysis not specified and no consideration has been given to any possibility of researcher bias	NO- No explanation given of the professional background of the researchers or the moderator for the focus groups, and there has been no evidence of any consideration of researcher bias at any point during the study.	NO- no mention of the background of the researchers or how this may have influenced the results	NO- role of second interviewer carrying out the in-depth interviews not mentioned, and there has been no consideration given to the possibility of bias from the interviewers. One of the interviewers was a FP, which could have led to bias with the interviewees responses.	NO- there has been no consideration given to the role of the researcher and the potential for researcher bias at any point.

							were considered.					
Have ethical issues been taken into consideration ?	YES	YES	YES- in the original studies, however further ethical issues regarding secondary qualitative analysis were not taken into account.	YES	YES	YES	YES	YES	YES	YES	YES	YES
Was the data analysis sufficiently rigorous?	YES	YES	YES	YES	NO- superficial description of analytic process and no information given on how many researchers analysed the transcripts- assumed one as there is only one author- risk of bias not taken into account for the analytic process	YES	YES	YES	YES	NO- the data analysis process is very ambiguous and the qualitative analysis has not been described in sufficient depth.	YES- clear description of the analytic process with two researchers independently analysing the data for rigour. However no description of the interpretation phase from the data.	NO- there is only a superficial description of the data analysis process, and there is very little detail given on how the themes were derived from the data. There is no presentation at all of quotes from the data to support the authors interpretation of the data.
Is there a clear	YES	YES	YES	YES	YES	YES	YES	YES	YES	YES	YES- however the qualitative	YES- however no quotes given

statement of findings?											data from the patient interviews has only been summarised- no direct quotes given	to support findings
How valuable is the research?	Valuable	Valuable	Valuable	Valuable	Valuable	Valuable	Valuable	Valuable	Valuable	Valuable	Valuable	Valuable

Newcastle-ottawa scale for cohort and observational studies summary table								
	Representativeness of the exposed cohort	Selection of the non-exposed cohort	Ascertainment of exposure	Demonstration that outcome of interest was not present at start of study	Comparability of cohorts on the basis of the design or analysis controlled for confounders	Assessment of outcome	Was follow-up long enough for outcomes to occur	Adequacy of follow-up of cohorts
Zulman et al [13]	Somewhat representative (one star) *	Drawn from the same community as the exposed cohort (one star) *	Secure record (one star) *	N/A	The study controls for age, sex and marital status (one star)*	Self-report	N/A	No statement

Axis tool for cross-sectional studies summary table

**Introduction**		Junius-Walker et al [14]	Fried et al[15]	Fried et al [16]	Moore et al [17]	Van Summeren et al [18]	Voigt et al [19]	Van Summeren et al [11]	Caughey et al [20]	Mantelli et al [21]	Deruaz-Luyet et al [22]	Herzig et al [23]
1	Were the aims/objectives of the study clear?	Yes	Yes	Yes	Yes	Yes	Yes	Yes	Yes	Yes	Yes	Yes
Methods												
2	Was the study design appropriate for the stated aim(s)?	Yes	Yes	Yes	Yes	Yes	Yes	Yes	Yes	Yes	Yes	Yes
3	Was the sample size justified?	No- convenience sampling used, small sample size, however no explanation for sample size given	No- no justification for sample size given, convenience sampling used	No- recruitment strategy described clearly but no justification for sample size given	Yes	No	No- sampling strategy described well but no justification for sample size given	No- purposive sampling used, however no justification for sample size given	No- no justification for sample size given	No- convenience sampling used and no justification for sample size given	Yes- in the parent study [24]	Yes- in the parent study [24]
4	Was the target/reference population clearly defined? (Is it clear who the research was about?)	Yes	Yes	Yes	Yes	Yes	Yes	Yes	Yes	Yes	Yes	Yes
5	Was the sample frame taken from an appropriate population base so that it closely represented the target/reference population under investigation?	Yes	Yes- However assumption made that participants will have multiple chronic conditions	Yes	Yes	Yes	Yes	Yes	Yes	Yes- although only GP's who had previously taken part in other case-vignette studies were invited, leading to possibility of selection bias	Yes	Yes

6	Was the selection process likely to select subjects/participants that were representative of the target/reference population under investigation?	Yes	Yes- as above	Yes	Yes	Yes	Yes	Yes	Yes	Yes- as above	Yes	Yes
7	Were measures undertaken to address and categorise non-responders?	Yes	Don't know- not reported	Yes	No	No	No	Yes- Purposive sampling used with efforts made to address gaps in participant types	Don't know- not reported	Don't know- not reported	Yes in the parent study [25].<br><br>Characteristics of participants who were not included due to missing data, were described in this study	Yes in the parent study [25]
8	Were the risk factor and outcome variables measured appropriate to the aims of the study?	Yes	Yes	Yes	Yes	Yes	Yes	Yes	Yes	Yes	Yes	Yes
9	Were the risk factor and outcome variables measured correctly using instruments/measurements that had been trialled, piloted or published previously?	Yes	Yes- piloted in a previous study	No- Tested in this study as it was a feasibility study	No- Pre-tested in this study but only using 2 FP's and 1 NP	Yes	No- STEP assessment previously published however no testing done of measure used to collect importance ratings	Yes	Yes	Yes- the instruments used were piloted within this study using 5 GP's as participants, but had not been published previously	No- instruments designed through "internal consensus discussions".	No

10	Is it clear what was used to determined statistical significance and/or precision estimates? (e.g. p-values, confidence intervals)	Yes	N/A	Yes	Yes	N/A	Yes	N/A	N/A	Yes	Yes	Yes
11	Were the methods (including statistical methods) sufficiently described to enable them to be repeated?	Yes	Yes	Yes	Yes	Yes	Yes	Yes	Yes	Yes	Yes	Yes
Results												
12	Were the basic data adequately described?	Yes	Yes	Yes	Yes	No- No reporting of prioritisation of patients for whom no medication changes were proposed	Yes	Yes	Yes	Yes	Yes	Yes
13	Does the response rate raise concerns about non-response bias?	No	Don't know-response rate not reported	No	No	No	Don't know-response rate not reported	No	Don't know-response rate not reported	No	No	No
14	If appropriate, was information about non-responders described?	Yes	No	Yes	No	Yes	No	Yes	No	No	Yes in the parent study[25]<br><br>Characteristics of participants who were not included	Yes in the parent study[25]

											due to missing data, were described in this study	
15	Were the results internally consistent?	Yes	Yes	Yes	Yes	Yes	Yes	Yes	Yes	Yes	Yes	Yes
16	Were the results presented for all the analyses described in the methods?	Yes	Yes	Yes	Yes	No- No reporting of prioritisation of patients for whom no medication changes were proposed	Yes	Yes	Yes	Yes	Yes	Yes
Discussion												
17	Were the authors' discussions and conclusions justified by the results?	No- very small sample of GP's compared to patients therefore generalizable conclusions regarding concordance between doctors and patients cannot accurately be drawn from this study	Yes	Yes	Yes	Yes	Yes	Yes- Small sample size for quantitative aspect of study taken into account	No- very small sample size across patients and clinicians, meaning results are not generalizable	Yes	Yes	Yes

18	Were the limitations of the study discussed?	Yes	No	Yes	Yes	Yes	Yes	Yes	Yes	Yes	Yes	Yes
Other												
19	Were there any funding sources or conflicts of interest that may affect the authors' interpretation of the results?	No	No	No	No	No	No	No	No	No	No	No
20	Was ethical approval or consent of participants attained?	Yes	Yes	Yes	Yes	Yes	Yes	Yes	Yes	Yes	Yes	Yes

The Cochrane Collaboration’s tool for assessing risk of bias in randomised controlled trials summary table		
Study	Junius-Walker et al [26]	
Bias	Authors’ judgement	Support for judgement
Random sequence generation (selection bias)	Low risk	“Participating doctors were allocated 1:1 into the intervention and control group using random block sizes of four”
Allocation concealment (selection bias)	Unclear risk	No information given regarding any efforts to conceal the allocation sequence
Blinding of participants and researchers (performance bias)	Low risk	Participants were only informed of the procedures of their own trial arm.
Blinding of outcome assessment (detection bias)	Low risk	Participants were blinded to the pre-intervention importance ratings, when completing the final importance ratings.
Incomplete outcome data (attrition bias)	High risk	25 patients dropped out prior to baseline ratings and 5 further patients dropped out prior to final ratings, these patients were excluded from analysis, however intention to treat analysis cannot be carried out in this context due to the nature of the intervention
Selective reporting (reporting bias)	Low risk	Adequate reporting on all of the specified outcomes
Other bias	None detected	

References

1 Kuluski K, Gill A, Naganathan G, et al. A qualitative descriptive study on the alignment of care goals between older persons with multi-morbidities, their family physicians and informal caregivers. *BMC Family Practice* 2013;14:133.

2 Schoenberg NE, Leach C, Edwards W. "It's a toss up between my hearing, my heart, and my hip": prioritizing and accommodating multiple morbidities by vulnerable older adults, *J Health Care Poor Underserved* 2009;20:134-51.

3 Cheraghi-Sohi S, Morden A, Bower P, et al. Exploring patient priorities among long-term conditions in multimorbidity: A qualitative secondary analysis. *SAGE Open Medicine* 2013;1:2050312113503955.

4 Naik A.D., Martin L.A., Moye J., et al. Health Values and Treatment Goals of Older, Multimorbid Adults Facing Life-Threatening Illness. *J Am Geriatr Soc* 2016;64:625-31.

5 LINDSAY S. PRIORITIZING ILLNESS: LESSONS IN SELFa MANAGING MULTIPLE CHRONIC DISEASES, *CANADIAN JOURNAL OF SOCIOLOGY/CAHIERS CANADIENS DE SOCIOLOGIE* 2009;34:983.

6 Hansen H., Pohontsch N., van den Bussche H., et al. Reasons for disagreement regarding illnesses between older patients with multimorbidity and their GPs - a qualitative study. *BMC family practice* 2015;16:68.

7 Morris R.L., Sanders C., Kennedy A.P., et al. Shifting priorities in multimorbidity: A longitudinal qualitative study of patient's prioritization of multiple conditions. *Chronic Illness* 2011;7:147-61.

8 Elliott RA, Ross-Degnan D, Adams AS, et al. Strategies for coping in a complex world: adherence behavior among older adults with chronic illness, *Journal of General Internal Medicine* 2007;22:805-10.

9 Fried TR, McGraw S, Agostini JV, et al. Views of older persons with multiple morbidities on competing outcomes and clinical decision-making, *J Am Geriatr Soc* 2008;56:1839-44.

10 Turner J.P., Edwards S., Stanners M., et al. What factors are important for deprescribing in Australian long-term care facilities? Perspectives of residents and health professionals. *BMJ Open* 2016;6:Arte Number: e009781. ate of Pubaton: 2016.

11 Summeren JJ, Haaijer-Ruskamp FM, Schuling J. Eliciting preferences of multimorbid elderly adults in family practice using an outcome prioritization tool, *J Am Geriatr Soc* 2016;64.

12 Caughey G.E., Huynh E., Shakib S., et al. Influence of medication risks and benefits on patient and clinician preferences for treatment in multimorbidity: A discrete-choice experiment. 2017.

13 Zulman D.M., Kerr E.A., Hofer T.P., et al. Patient-provider concordance in the prioritization of health conditions among hypertensive diabetes patients. *Journal of General Internal Medicine* 2010;25:408-14.

14 Junius-Walker U, Stolberg D, Steinke P, et al. Health and treatment priorities of older patients and their general practitioners: a cross-sectional study. *Quality in primary care* 2011;19.

15 Fried TR, Tinetti ME, Iannone L, et al. Health outcome prioritization as a tool for decision making among older persons with multiple chronic conditions, *Arch Intern Med* 2011;171:1856-8.

16 Fried TR, Tinetti M, Agostini J, et al. Health outcome prioritization to elicit preferences of older persons with multiple health conditions, *Patient Educ Couns* 2011;83:278-82.

17 Moore A., Patterson C., Nair K., et al. Minding the gap: Prioritization of care issues among nurse practitioners, family physicians and geriatricians when caring for the elderly. *Journal of interprofessional care* 2015;29:401-3.

18 Van Summeren J.J.G.T., Schuling J., HaaijerRuskamp F.M., et al. Outcome prioritisation tool for medication review in older patients with multimorbidity: A pilot study in general practice. *British Journal of General Practice* 2017;67:e501-6.

19 Voigt I, Wrede J, Diederichs-Egidi H, et al. Priority setting in general practice: health priorities of older patients differ from treatment priorities of their physicians, *Croat Med J* 2010;51:483-92.

20 Caughey G.E., Tait K., Vitry A.I., et al. Influence of medication risks and benefits on treatment preferences in older patients with multimorbidity. *Patient Preference and Adherence* 2017;11:131-40.

21 Mantelli S, Jungo KT, Rozsnyai Z, et al. How general practitioners would deprescribe in frail oldest-old with polypharmacy—the LESS study, *BMC family practice* 2018;19:169.

22 Déruaz-Luyet A, N'Goran AA, Pasquier J, et al. Multimorbidity: can general practitioners identify the health conditions most important to their patients? Results from a national cross-sectional study in Switzerland, *BMC family practice* 2018;19:66.

23 Herzig L, Mueller Y, Haller DM, et al. Family practitioners' top medical priorities when managing patients with multimorbidity: a cross-sectional study, *BJGP open* 2019;3:bjgpopen18X101622.

24 DeruazLuyet A., Alexandra N'Goran A., Tandjung R., et al. Multimorbidity in primary care: Protocol of a national cross-sectional study in Switzerland. *BMJ Open* 2015;5:Arte Number: e009165. ate of Pubaton: 2015.

25 Deruaz-Luyet A, N'Goran AA, Senn N, et al. Multimorbidity and patterns of chronic conditions in a primary care population in Switzerland: a cross-sectional study, *BMJ Open* 2017;7:e013664,2016-013664.

26 Junius-Walker U, Wrede J, Voigt I, et al. Impact of a priority-setting consultation on doctor-patient agreement after a geriatric assessment: cluster randomised controlled trial in German general practices. *Quality in primary care* 2012;20.
