# Supplementary material for: Priorities of patients with multimorbidity and of clinicians regarding treatment and health outcomes: a systematic mixed studies review
Source: BMJ Open. 2020 Feb 12;10(2):e033445. doi: 10.1136/bmjopen-2019-033445 (PMC7045037; doi:10.1136/bmjopen-2019-033445)
Supplement: Author's manuscript [file bmjopen-2019-033445.draft_revisions.pdf]

BMJ Open is committed to open peer review. As part of this commitment we make the peer review history of every article we publish publicly available.

When an article is published we post the peer reviewers' comments and the authors' responses online. We also post the versions of the paper that were used during peer review. These are the versions that the peer review comments apply to.

The versions of the paper that follow are the versions that were submitted during the peer review process. They are not the versions of record or the final published versions. They should not be cited or distributed as the published version of this manuscript.

BMJ Open is an open access journal and the full, final, typeset and author-corrected version of record of the manuscript is available on our site with no access controls, subscription charges or pay-per-view fees (http://bmjopen.bmj.com).

If you have any questions on BMJ Open's open peer review process please email info.bmjopen@bmj.com

BMJ Open

A systematic mixed studies review of treatment and health outcome priorities of multi-morbid patients and clinicians

Journal:	*BMJ Open*
Manuscript ID	bmjopen-2019-033445
Article Type:	Research
Date Submitted by the Author:	05-Aug-2019
Complete List of Authors:	Sathanapally, Harini; University of Leicester Diabetes Research Centre,<br>Sidhu, Manbinder S.; University of Birmingham,<br>Fahami, Radia; University of Leicester Diabetes Research Centre<br>Gillies, Clare; University of Leicester Diabetes Research Centre<br>Kadam , Umesh ; University of Leicester,<br>Davies, Melanie; University of Leicester Diabetes Research Centre<br>Khunti, Kamlesh; University of Leicester Diabetes Research Centre<br>Seidu, Samuel; University of Leicester Diabetes Research Centre
Keywords:	PRIMARY CARE, GENERAL MEDICINE (see Internal Medicine), QUALITATIVE RESEARCH

SCHOLARONE™
Manuscripts

I, the Submitting Author has the right to grant and does grant on behalf of all authors of the Work (as defined in the below author licence), an exclusive licence and/or a non-exclusive licence for contributions from authors who are: i) UK Crown employees; ii) where BMJ has agreed a CC-BY licence shall apply, and/or iii) in accordance with the terms applicable for US Federal Government officers or employees acting as part of their official duties; on a worldwide, perpetual, irrevocable, royalty-free basis to BMJ Publishing Group Ltd ("BMJ") its licensees and where the relevant Journal is co-owned by BMJ to the co-owners of the Journal, to publish the Work in this journal and any other BMJ products and to exploit all rights, as set out in our licence.

The Submitting Author accepts and understands that any supply made under these terms is made by BMJ to the Submitting Author unless you are acting as an employee on behalf of your employer or a postgraduate student of an affiliated institution which is paying any applicable article publishing charge ("APC") for Open Access articles. Where the Submitting Author wishes to make the Work available on an Open Access basis (and intends to pay the relevant APC), the terms of reuse of such Open Access shall be governed by a Creative Commons licence – details of these licences and which Creative Commons licence will apply to this Work are set out in our licence referred to above.

Other than as permitted in any relevant BMJ Author's Self Archiving Policies, I confirm this Work has not been accepted for publication elsewhere, is not being considered for publication elsewhere and does not duplicate material already published. I confirm all authors consent to publication of this Work and authorise the granting of this licence.

For peer review only

A systematic mixed studies review of treatment and health outcome priorities of multi-morbid patients and clinicians

Harini Sathanapally[1], Manbinder Sidhu[2], Radia Fahami[1], Clare Gillies[1], Umesh Kadam[1], Melanie J Davies[1], Kamlesh Khunti[1] and Samuel Seidu[1]

1. *Diabetes research centre, College of Life Sciences, University of Leicester, UK*
2. *Health Services Management Centre, School of Social Policy, University of Birmingham, UK*

Corresponding author: Dr Harini Sathanapally (hs333@student.le.ac.uk)

Word count excluding titles, abstract, article summary, tables, figures and references: 4414

Author contributions: HS: Design of research question and methodology, data searching, data extraction, data analysis and manuscript development; MS: Design of methodology, data extraction, data analysis and manuscript development; RF: Data searching and data extraction; CG: Data analysis and manuscript development; UK: Data analysis and manuscript development; MJD: Data analysis and manuscript development; KK: Design of research question and methodology, manuscript development; SS: Conception of the idea for this review, design of research question and methodology, data extraction, data analysis and manuscript development. All authors have approved the final manuscript.

Funding: Harini Sathanapally is funded by the NIHR academic clinical fellowship award

Competing interests: None declared

Patient consent for publication: Not required

Data sharing statement: The majority of the data extracted and analysed as part of this review has been included in this article and associated appendices. Any further data can be acquired from the corresponding author by request.

Patient and public involvement: Patient and public involvement was not applicable in the design, conduct or reporting of this review.

For peer review only

ABSTRACT

Objectives : To identify studies that have investigated the health outcome and treatment priorities of multi-morbid patients, clinicians, or both, in order to assess whether the priorities of the two groups are in alignment, or whether there is a disparity between the priorities of multi-morbid patients and clinicians.

Design: Systematic review

Data sources: MEDLINE, EMBASE, CINHAL and Cochrane databases from inception to May 2019 using a pre-defined search strategy, as well as reference lists containing any relevant articles, as per PRISMA and Cochrane guidelines.

Eligibility criteria: We included studies reporting health outcome and treatment priorities of adult multi-morbid patients, or of clinicians in the context of multi-morbidity, or both. There was no restriction by study design, and studies using quantitative and/or qualitative methodologies were included.

Data synthesis: We used a narrative synthesis approach to synthesise the quantitative findings, and a meta-ethnography approach to synthesise the qualitative findings.

Results: Our search resulted in the identification of 24 studies for inclusion, which comprised of 12 quantitative studies, 10 qualitative studies and 2 mixed-methods studies. Twelve studies reported the priorities of both patients and clinicians (7 quantitative, 3 qualitative and 2 mixed-methods studies), ten studies reported the priorities of patients alone (3 quantitative and 7 qualitative studies) and two studies reported the priorities of clinicians alone (2 quantitative studies).

Conclusion: Our findings have shown that there is a mostly low level of agreement between the priorities of multi-morbid patients and clinicians. We found that prioritisation by multi-morbid patients was mainly driven by their illness experiences, whilst clinicians focused on longer term risks. Recognising that there may be a disparity in prioritisation and understanding the reasons for why this might occur, can facilitate clinicians in accurately eliciting the priorities that are most important to their patients and delivering patient-centred care.

KEY WORDS: Patient-centred care, Shared decision-making, Multi-morbidity

ARTICLE SUMMARY

Strengths and limitations

- This is the first systematic review to assimilate and compare the findings of existing literature on the health outcome and treatment priorities of both clinicians treating and patients living with multi-morbidity.
- We have included papers using both qualitative and quantitative methodologies and have been able to explore patterns and relationships in the findings, thus creating a comprehensive and well-rounded systematic review.
- Our findings facilitate clinicians in understanding both *how* and *why* the health outcome and treatment priorities of their multi-morbid patients might differ from their own priorities.
- Meta-analysis of the quantitative studies was unfeasible as there was a large variation in the tools used to ascertain priorities, and we have attempted to mitigate this by using a well-described and transparent method of narrative synthesis.
- A number of our included quantitative studies did not use pre-validated tools to ascertain priorities, leading to a risk of measurement bias.

For peer review only - http://bmjopen.bmj.com/site/about/guidelines.xhtml

INTRODUCTION

Multi-morbidity, defined as the co-existence of two or more long-term conditions [1] is a global problem [2], which has become the norm across high-income countries [2, 3][4, 5] and becoming increasingly prevalent in middle and low-income countries [6][7][2]. Guidelines for the management of chronic diseases are often single disease-orientated, and can lead to confusion and complications when applied to patients with multi-morbidity [8]. Multi-morbid patients have an increased risk of adverse drug-related events as a result of high levels of polypharmacy and receiving un-coordinated care from multiple healthcare providers [9]. These patients have a poorer health-related quality of life [10], poorer functional status [11] and greater psychological distress [12]. As a result, understanding and finding better strategies to facilitate the management of multi-morbid patients has been identified as a priority for health research [13] .

Key to the effective management of multi-morbidity is using patient-centred care and shared decision-making to set management goals that are acceptable to both the patient and the clinician [14]. Incorporating the priorities of patients in relation to treatments and health outcomes is integral to this process [15-17]. However, previous research has shown that whilst doctors recognise the importance of eliciting and incorporating the priorities of their multi-morbid patients, they do not always engage with this process in real world settings, and find eliciting patients' priorities to be difficult [18] [19]. Previous research, completed in a single disease context, has shown that the treatment and health outcome priorities of patients and clinicians can differ [20-22], and some studies have highlighted a gap between what doctors' perceive to be the priorities of their patients, and the actual priorities of their patients [23-25].

This systematic review aims to identify studies that have investigated the health outcome and treatment priorities of multi-morbid patients, clinicians, or both, in order to assess whether the priorities of the two groups are in alignment, or whether there is a disparity between the priorities of multi-morbid patients and clinicians.

METHODS

Search strategy

This systematic review has been registered on PROSPERO (ID: CRD42018076076). A comprehensive search strategy (Appendix 1), was developed using guidance for best practice [26] and input from academic librarians at the University of Leicester. The search strategy was used to search MEDLINE, EMBASE, CINHAL and COCHRANE databases from inception to May 2019, as well as searching reference lists for any relevant articles based on PRISMA and Cochrane guidelines [26-28]. Citations were stored using Refworks. We have presented our process of article selection in Figure 1.

We included studies reporting the health outcome and treatment priorities of adult patients with multimorbidity [1] and/or clinicians, in relation to patients with multi-morbidity. Studies which did not specify the definition of multi-morbidity as "two or more chronic conditions" [1] in their inclusion criteria, but had a sample patients representative of being diagnosed with multi-morbidity (i.e. with a minimum of two chronic conditions), were also included. There was no restriction by study design, and we included studies using quantitative and/or qualitative methodologies. We excluded studies not published in English language, studies with participants aged under 18 years, and studies focusing on a single disease area.

For peer review only - http://bmjopen.bmj.com/site/about/guidelines.xhtml

Study selection

The titles and abstracts of all articles identified by the literature search were assessed independently and in duplicate by two reviewers (HS and RF). Studies that did not meet inclusion criteria were discarded. Full text of selected articles were retrieved and assessed to determine if they met the inclusion criteria, and those studies which met the inclusion criteria were included in the review. Any discrepancies regarding eligibility of an article were discussed, and consensus reached with MS and SS.

Methodological quality assessment and data extraction

Data was extracted using standardised data extraction forms by a single reviewer (HS), and these were checked independently for accuracy by a second reviewer (SS). The reported health outcome and treatment priorities of study participants were the key outcomes that were extracted.

Quality assessment was carried out in parallel with the data extraction process. For the quantitative studies, due to the heterogeneity of study design, we used the AXIS tool for assessment for the cross-sectional studies [29], the Newcastle-Ottawa scale for assessment of the longitudinal observational and cohort studies [30], and the Cochrane collaboration's risk of bias tool for assessment of randomised controlled trials [31]. For the qualitative studies, we used the CASP checklist for appraisal of qualitative research [32]. For the two mixed-methods studies, we used the AXIS tool [29] to assess the quantitative aspects of the study (both cross-sectional in study design), and the CASP checklist for qualitative research [32], to assess the qualitative aspects of these studies.

Data synthesis

We decided *a priori* not to carry out a meta-analysis due to the heterogeneity of the quantitative studies. Therefore, we have taken a narrative synthesis approach, described by Popay et al [33] to synthesise our quantitative findings. Our approach consists of three key steps:

1) *Development of a preliminary synthesis* in which study characteristics and descriptions are collated and findings presented in a summary table

2) Exploring relationships in the data between study characteristics and their findings, as well as between the reported findings across different studies with explanations considered where relationships were identified.

c) *Assessing the robustness of the synthesis using* quality assessment tools to guide conclusions and identify directions for clinical practice.

Qualitative studies were synthesised using a meta-ethnography approach [34, 35], which consisted of careful reading of the papers, extracting information regarding the context of the study and findings. *Key concepts* arising from each paper were also identified, with preservation of the terminology used by the authors where possible to ensure accurate representation of the findings of the original studies. The key concepts across the papers were then *translated* using a table summarising the studies, their findings in relation to the key concepts and the *second order* interpretations of the authors, which enabled the exploration of any relationships and differences. The translations were then synthesised using a table containing the *first order* and *second order* interpretations for the key concepts across the studies, which then led to the development of further, *third order* interpretations by reviewers [34, 35].

For peer review only - http://bmjopen.bmj.com/site/about/guidelines.xhtml

RESULTS

Overall study characteristics

Our search resulted in the identification of 24 studies for inclusion, which comprised of 12 quantitative studies, 10 qualitative studies and 2 mixed-methods studies. The characteristics of all of the included studies are described in Table 1. The included studies had all been conducted in high income developed countries, including Canada [36, 37], USA[38-45], Netherlands[46, 47], Australia[48, 49], UK[50-52], Germany [53-56]and Switzerland [57-59]. Sample sizes ranged from 15 to 1169 patients and 5 to 92 clinicians in the quantitative studies, and 15 to 146 patients and 4 to 19 clinicians in the qualitative studies.

Author and year of publication	Setting	Study type	Study aims	Target group and number of participants (n)	Outcomes measured
**QUANTITATIVE**					
Moore et al, 2014 [36]	Canada- Databases of all practising nurse practitioners, family practitioners and geriatricians in Ontario	Quantitative: Cross-sectional survey	*To quantify how family physicians, nurse practitioners and geriatricians prioritize syndromes, diseases and conditions when caring for seniors*	Nurse practitioners (n=68)<br><br>Family practitioners (n=84)<br><br>Geriatricians (n=27)	Frequency and importance rankings given by family practitioners, nurse practitioners and geriatricians to 41 health issues known to arise in elderly patients
Fried et al, 2011 [39]	USA- 3 senior centres and 1 assisted living facility	Quantitative: Cross-sectional study	*To explore the use of a simple tool to elicit older persons' health outcome priorities*	All volunteers included (n=357)	The prioritisation by participants of 4 universal health outcomes, namely:<br>-keeping alive<br>- maintaining independence<br>- reducing or eliminating pain<br>-reducing or eliminating other symptoms
Fried et al , 2011, [40]	USA- recruited from participants in a larger study, where they had been recruited from age-aggregated community housing [60]	Quantitative: Cross-sectional survey	*To determine the feasibility of using a simple tool to elicit the preferences of older persons based on their prioritization of universal outcomes*	Patients aged 65 and over with a known diagnosis of hypertension or use of anti-hypertensive medications, and having a known risk of falls (n=81)	> Rankings given by participants to 4 universal health outcomes in the outcome prioritisation tool:<br>--keeping alive<br>- maintaining independence<br>- reducing or eliminating pain<br>-reducing or eliminating other symptoms<br>> Feasibility of the use of outcome prioritisation tool
Fried et al, 2006 [42]	USA- sub-speciality outpatient practices, a community hospital, a university teaching hospital and a veterans administration hospital.	Quantitative: Longitudinal observational study	*To examine changes over time in end-of-life treatment preferences, measured in terms of willingness to undergo treatment based on the health state that would result from the treatment, in a cohort of older persons with advanced chronic illness*	Patients aged 60 or over with a primary diagnosis of cancer, congestive heart failure or chronic obstructive pulmonary disease and need assistance with at least 1 instrumental activity of daily living (n=226 at baseline, 98 at follow up)	Patient reported acceptability of four health states that could result from treatment (at baseline and 4 monthly intervals over 2 years) namely:<br>-unable to leave house<br>-only able to get from bed to chair<br>-Severe memory problems<br>-Daily pain

For peer review only

For peer review only - http://bmjopen.bmj.com/site/about/guidelines.xhtml

Zulman et al, 2010 [45]	USA- Scheduled primary care visit for patients at 9 veteran affairs facilities	Quantitative: Prospective cohort study	*To understand patterns of patient-provider concordance in the prioritization of health conditions in patients with multimorbidity*	Patients with diabetes and hypertension who had their primary diabetes care provider enrolled in the study (n = 1169)<br><br>Primary care providers i.e. physicians, physician assistants or nurse practitioners (n= 92)	-Patient rankings given in terms of their most important health concerns and providers rankings in terms of conditions most likely to affect each patient's outcomes<br>-Concordance between the importance ratings of patient-provider "pairs"
Van Summeren et al, 2017 [47]	Netherlands-General practice centres	Quantitative: Cross-sectional and implementation study	*To determine proposed and observed medication changes when using an outcome prioritisation tool during a medication review in older patients with multimorbidity and polypharmacy. A secondary aim was to explore the relationship between the prioritized health outcome of patients and the type of medication change, such as a stop, a dose adjustment, or a switch.*	Patients aged 69 or over with two or more chronic diseases (one of which had to be cardiovascular disease) and daily use of five or more medications. (n=59)<br><br>General practitioners (n=17)	>Patients' priority rankings of the four health outcomes in the outcome prioritisation tool:<br>-Maintaining independence<br>-Remaining alive<br>-Reducing other symptoms<br>-Reducing pain<br><br>>Medication changes proposed by the GP, and observed in the patient records following incorporation of the priority rankings given by patients, into a medication review consultation.
Junius-Walker et al 2012 [53]	Germany- General practice centres	Quantitative: Randomised controlled trial	*To investigate whether a structured priority-setting consultation reconciles the often-differing doctor–patient views on the importance of problems.*	Patients aged 70 or over (n=317)<br><br>General practitioners (n=40)	-Baseline importance rankings given by patients and clinicians to a list of problems generated from a geriatric assessment for each patient.<br>- Importance rankings given again after a structured consultation incorporating the baseline problem list and importance rankings and degree of reconciliation in doctor-patient agreement after the structured consultation
Junius-Walker et al, 2011[54]	Germany- General practice centres	Quantitative: Cross-sectional survey	*To gain insight into setting individual priorities with older patients using a priority definition that was coherent to the patients' life and doctors' work context*	Patients aged 70 or over and living at home (n=123)<br><br>General practitioners (n=11)	Importance rankings given by patients and clinicians to a list of problems generated from a geriatric assessment for each patient.
Voigt et al, 2010 [55]	Germany-General practice centres	Quantitative: Cross-sectional survey	*To ascertain health priorities of older patients and treatment priorities of their general practitioners (GP) on the basis of a*	Patients aged 70 or over and at least one contact with the general practitioner in the preceding 3 months (n= 35)	-Importance rankings given to problems generated from a geriatric assessment by patients and clinicians<br>-Degree of agreement between patients and clinicians on the above

For peer review only - http://bmjopen.bmj.com/site/about/guidelines.xhtml

			geriatric assessment and to determine the agreement between these priorities.	General practitioners (n=9)	
Herzig et al, 2019 [57]	Switzerland- Primary data was from "Multimorbidity in Family medicine" study[61].<br><br>Patients enrolled by General practitioners during scheduled consultations.	Quantitative: Cross-sectional survey	*To describe FPs' medical priority ranking of conditions relative to their prevalence in patients with multimorbidity*	Patients suffering from at least 3 of 75 chronic conditions on a pre-defined list (based on the International classification of primary care 2 (n=888)<br><br>General Practitioners (n=100)	Importance rankings given by family practitioners to the list of chronic conditions that each patient had on the day of their inclusion in the study
Mantelli et al, 2018[58]	Switzerland- General practitioners working in Switzerland who had previously taken part in case-vignette studies	Quantitative: cross-sectional survey	*To determine whether, how and why GPs de-prescribe in frail oldest-old patients with multimorbidity and polypharmacy, and to identify factors that influenced their decision to de-prescribe*	General Practitioners (n=157)	- Percentage of GPs willing to de-prescribe at least one medication in the case of frail older patients with CVD and compared to frail older patients without CVD<br>- Reasons for de-prescribing<br>- Importance ratings given to factors influencing decision to de-prescribe
Déruaz-Luyet et al, 2018 [59]	Switzerland- Primary data was from "Multimorbidity in Family medicine" study [61].<br><br>Patients enrolled by General practitioners during scheduled consultations.	Quantitative: Cross-sectional survey	*To evaluate whether GPs could identify the condition that their patients with multimorbidity considered most important.*	Patients suffering from at least 3 of 75 chronic conditions on a pre-defined list (based on the International classification of primary care 2, and receiving follow-up from their GP for at least the preceding 6 months (n= 572 for main analysis, 585 for sensitivity analysis)<br><br>General Practitioners (n=100)	Whether there is agreement between what patients considered to be their most important health condition and what GPs thought patients considered to be their most important health condition
**MIXED-METHODS**					
Van Summeren et al, 2016 [46]	Netherlands- General practice centres	Mixed-methods: Cross-sectional survey pilot and qualitative interviews to assess acceptability (semi-structured and indepth)	*To explore whether an outcome prioritization tool (OPT) is appropriate in the context of medication review in family practice, focusing on its acceptability and practicality*	Patients aged 69 or over with two or more chronic diseases (one of which had to be cardiovascular disease) and daily use of five or more medications (n=60)<br><br>General practitioners (n=13)	>Patients' prioritisation of the four domains of the outcome prioritisation tool:<br>-Maintaining independence<br>-Remaining alive<br>-Reducing other symptoms<br>-Reducing pain<br><br>> Family practitioners views on the acceptability and practicality of using the outcome prioritisation tool for medication review

For peer review only - http://bmjopen.bmj.com/site/about/guidelines.xhtml

Caughey et al, 2017 [48]	Australia- Multi-disciplinary ambulatory consulting service clinics at tertiary teaching hospitals	Mixed-methods: Structured quantitative interviews with patients then semi-structured qualitative interviews with patients and clinicians	*To investigate how older patients with multi-morbidity balance the benefits and harms associated with medication for prevention of CVD, and in the presence of competing health outcomes. To investigate the factors that clinicians consider when making treatment decisions for older, multimorbid patients.*	Patients aged 65 or older with 2 or more chronic conditions (n=15)<br><br>Clinicians (n=5)	-Patient willingness to take a medication when presented with different scenarios with variable degree of benefit, impact on daily living, adverse outcomes and impact on other comorbid conditions<br><br>-Patient-reported data during semi-structured interviews where they were asked about their treatment preferences, medication effects and shared decision making<br><br>-Clinician reported data during semi-structured interviews on treatment decisions, patient preferences and polypharmacy.
**QUALITATIVE**					
Kuluski et a, 2013 [37]	Canada- A Family Health Team in Ontario	Qualitative: Semi-structured interviews	*To examine patient goals of care from the perspectives of older persons with multi-morbidities, their family physicians and informal caregivers (i.e., family member or friend who provides ongoing support) and then examine the extent of alignment between these three perspectives*	Patients aged 65 or older with a diagnosis of at least two chronic health conditions (n=28)<br><br>Informal Caregivers of included patients (n=28)<br><br>Family physicians (n=4)	>Patient, caregiver and physician reported data on goals of care for the patients<br><br>>Degree of alignment of goals of care across patient, caregiver and physician “triads”
Schoenberg et al, 2009 [38]	USA- Senior centres, Low income senior housing complexes, churches and a civic meeting hall	Qualitative: In-depth interviews	*To understand how vulnerable older adults with multimorbidity prioritize and manage their chronic conditions*	Patients aged 55 or older with a diagnosis of at least two chronic illnesses, from low-income backgrounds (n= 41)	Patient-reported data from in-depth interviews, regarding their previous health, perceptions and self-care procedures in relation to their multi-morbidity
Fried et al, 2008 [41]	USA- Senior centres, Doctors' practices and a congregate housing site	Qualitative: Focus groups	*To examine the ways in which older persons with multiple conditions think about potentially competing outcomes, in order to gain insight into how processes to elicit values regarding these outcomes can be grounded in the patient's perspective*	Patients aged 65 or older and were taking 5 or more medications (participants also had a minimum of 3 chronic conditions)	Patient-reported data regarding their perceptions of the interactions between their different illnesses and treatment regimens, goals of treatment and decisions regarding treatment

For peer review only - http://bmjopen.bmj.com/site/about/guidelines.xhtml

Naik et al, 2016 [43]	USA- Qualitative data from the VETCARES study [62] , in which participants recruited from the VA tumour registry	Qualitative: Open-ended questions as part of mixed methods interviews which also included structured questions	*To identify a taxonomy of health-related values that frame goals of care of older, multi-morbid adults who recently faced cancer diagnosis and treatment*	Veterans with a diagnosis of head and neck, gastric, oesophageal, or colorectal cancer, and diagnosis fell one month prior to the study's opening eligibility window (6 months) (n=146)	Patient-reported data regarding their priorities or concerns regarding their future healthcare decisions
Elliott et al, 2007 [44]	USA- Harvard Pilgrim Health Centre, a HMO (health maintenance organisation) in New England	Qualitative: Semi-structured interviews	*To explore how older adults with multiple illnesses make choices about medicines*	Patients taking more than three medicines with purposive sampling to reflect symptomatic comorbidities and asymptomatic comorbidities and mental health issues (participants had a minimum of 3 comorbidities) (n=20)	Patient-reported data regarding beliefs about medicines, medicine-taking behaviour, historical vs potential choices between different medicines, and factors influencing these choices
Turner et al, 2016 [49]	Australia- Long term care facilities in South Australia	Qualitative: Nominal group technique	*To use nominal group technique to generate then rank factors that general medical practitioners, nurses, pharmacists and residents or their representatives perceive are most important when deciding whether or not to de-prescribe medication*	Residents/representatives of residents (n=11)<br><br>General Practitioners (n=19)<br><br>Nurses (n=12)<br><br>Pharmacists (n=14)	-Generated factors important for de-prescribing according to residents/resident representatives, general practitioners, nurses and pharmacists<br><br>-Priority rankings given by groups containing representatives from all of the above, to the list of priorities generated previously.
Lindsay, 2009 [50]	UK- Participants recruited from CHD registries in Greater Manchester as part of a larger RCT[63]	Qualitative: Focus groups and two interviews	*To use the concepts of "chronic illness trajectory" and "biographical disruption" to examine how patients self-manage multiple chronic conditions and especially how they prioritize their conditions*	Participants from the parent study who had more than one chronic condition (i.e. at least two) (n=53)	Patient-reported data regarding how they prioritised their multiple conditions, what strategies they used to cope with their conditions and barriers in being able to manage their illnesses
Cheraghi-Sohi et al, 2013 [51]	UK- secondary analysis of qualitative data from four other studies [64-67]	Qualitative: In-depth interviews	*To explore how and why people with multimorbidity prioritise some long-term conditions over others and what the potential implications may be for self-management activity, and in turn, suggest how such information may help clinicians negotiate the management of*	Participants from original studies who had two or more long term conditions, and had given data regarding prioritisation (n=41)	Patient-reported data pertaining to prioritisation of their long term conditions

For peer review only

For peer review only - http://bmjopen.bmj.com/site/about/guidelines.xhtml

			multimorbidity patients		
Morris et al [52]	UK- General Practices in North-West England	Qualitative: Semi-structured interviews	*To examine what influences self-management priorities for individuals with multiple long-term conditions and how this changes over time*	Patients with more than one chronic condition and at least one of COPD, IBS or Diabetes (n=21)	Patient-reported data on management strategies and experiences with primary health care, and data from follow-up interviews on any changes in their illness management.
Hansen et al, 2015 [56]	Germany- Participants recruited from the "Multicare cohort study" [68]	Qualitative: Focus groups	*To identify reasons for disagreement regarding illnesses between patients and their GPs*	Patients who had 3 or more chronic conditions from a list of 29 conditions (n=21)<br><br>General Practitioners of the recruited patients (n=15)	Data from separate focus groups for patients and clinicians in which any communication problems and reasons for disagreement between patients and clinicians were explored

Table 1 Characteristics of all of the included studies in order of reference

Summary of quality assessment

The outcome of quality assessment based on each of the afore-mentioned tools is summarised in Appendix 2. The majority of the quantitative studies were cross-sectional in design [36, 39, 40, 46, 47, 54, 55, 57-59] [48], including the quantitative elements of the two mixed-methods studies. The other studies included one longitudinal observational study [42], one cohort study [45] and one randomised controlled trial [53]. The cross-sectional studies were of moderate quality, with a number of studies having small sample sizes [40, 46, 47, 55]. The sample sizes of clinicians in most of the cross-sectional studies were particularly small, ranging from of 9 to 157 clinicians [46, 47, 55, 58], which impacts upon the generalisability and application of their findings. We noted that a number of the studies did not use pre-validated questions and tools to ascertain priorities [36, 55, 57-59], leading to a degree of subjectivity in the way in which priorities were ascertained, and the risk of measurement bias which again impacts on the generalisability of their findings.

The majority of the qualitative studies, including the qualitative aspects of the two mixed-methods studies, used interviews for data collection (n=8). Two studies used focus groups [41, 56], one study used a combination of focus groups and interviews [50] and one study used the nominal group technique [49]. The qualitative studies were of good quality, with appropriate use of qualitative methodology and transparent descriptions of the data analysis processes. Three studies only gave a limited description of their analytic process [48, 49]**,** with two of these studies not presenting any quotes [48, 49]**.**

QUANTITATIVE SYNTHESIS

Within our quantitative synthesis, we found that the studies focused either on the overall state of the patients' health, the problems posed by different chronic disease groups, or the patients' treatment regimens. Some of the quantitative studies elicited patient and/or clinician priorities as part of an intervention [53] [47]. Therefore, in order to reduce the risk of bias from the interventions, we included only the pre-intervention results from these studies.

Health outcome priorities

Four studies reported patient priorities of overall health outcomes using a "health outcome prioritisation tool" [39, 40, 46], which is a visual analogue scale requiring the following health

For peer review only - http://bmjopen.bmj.com/site/about/guidelines.xhtml

outcomes to be given a score out of 100: "Maintaining independence"; "Staying alive"; "Pain relief"; "Symptom relief". Maintaining independence was the outcome that had the highest importance after a pooling of the *most important* rankings from the four studies, followed by "Staying alive" (Table 2). For clinicians' priorities, one study reported that 98% of a sample of 157 general practitioners identified the "quality of life for the patient", and 96% identified the "life expectancy of the patient", as the most important factors in influencing their clinical decision-making to de-prescribe for elderly, multi-morbid patients [58].

Study	*Health outcome prioritisation as a tool for decision making among older persons with multiple chronic conditions*[39]	*Health outcome prioritisation to elicit preferences of older persons with multiple health conditions*[40]	*Outcome prioritisation tool for medication review in older patients with multimorbidity: A pilot study in general practice*[47]	*Eliciting Preferences of multi-morbid Elderly Adults in Family Practice Using an Outcome Prioritisation Tool*[46]	*Aggregate ranking as most important (%)*
**Maintaining independence**	270 (75.6%)	34 (42.0%)	7 (36.8%)	19 (35.8%)	330 (64.7%)
**Staying alive**	40 (11.2%)	22 (27.2%)	6 (31.6%)	18 (34.0%)	86 (16.9%)
**Pain relief**	26 (7.3%)	17 (21.0%)	1 (5.3 %)	6 (11.3%)	50 (9.8%)
**Symptom relief**	21 (5.9%)	8 (9.8%)	5 (26.3%)	10 (18.9%)	44 (8.6%)
**Total number of participants**	357	81	19[a]	53	510

Table 2-Summary of most important rankings for studies using the Outcome Prioritisation Tool
a= although there were 59 patients included in this study [47] *priorities were only reported for 19 patients*

Priorities based on health problems

Three studies reported patient and general practitioners' priorities based on various health *problems*, following a geriatric assessment [53-55]. These problems were then categorised into domains, and the importance rankings for each of the domains were presented. Problems in the domains of "Social" "Mood" and "Function" recurrently featured in the top four of the most highly ranked priorities by patients across all three studies. In terms of the importance rankings by clinicians, problems in the domains of "Mood" and "Function" also featured in the top four importance rankings across all three studies, whilst "Social" problems were rated highly in one study [54] and problems in the domain of "Medication" were ranked highly in the other two studies [53, 55]. Interestingly, the authors in one study[54] found that patients feeling "Emotionally affected" was the strongest predictor for a problem being rated as important (OR 11.1 CI 6.73 to 18.33), whereas "Poor prognosis" was the strongest predictor for clinicians (OR 6.39 CI 4.61 TO 8.87)

Disease-specific priorities

Two studies reported patient priorities in relation to specific diseases or disease groups [45, 59]. Zulman et al. reported that "Diabetes/glycaemic control" was most frequently ranked as "most important", with "Hypertension" coming second [45]. However, the sample of patients included in this study were all diabetic, hypertensive patients. Deruaz-luyet et al. found that musculoskeletal conditions including back pain, were most frequently reported to be the most important conditions for their patients, however endocrine/metabolic conditions (including obesity) were second and cardiovascular conditions were third [59].

For peer review only - http://bmjopen.bmj.com/site/about/guidelines.xhtml

Three studies reported disease-specific, or disease-group-specific priorities of clinicians. Herzig et al. reported the priorities of general practitioners alone [57], and found that “multiple sclerosis”, “mental retardation”, and “bronchus lung neoplasm” were all highly prioritised by their participants. Zulman et al. reported the priorities of “primary care providers” who consisted of physicians, physician assistants or nurse practitioners [45], and found that diabetes was the top priority for primary care providers, with hypertension coming second, in alignment with their previously described patient priorities [45]. Moore et al. examined the priorities of different types of clinicians, including family physicians, geriatricians and nurse practitioners [36], and as with Zulman et al., found that diabetes was the top priority for family physicians and also nurse practitioners, whereas dementia was the top priority for geriatricians [36]. In addition, heart failure, atrial fibrillation and hypertension formed three of the top five conditions considered to be most important by the family practitioners in the study [36].

Treatment priorities

Fried et al. examined patient-ratings as *acceptable vs unacceptable* for four adverse outcomes from treatment, namely ‘unable to leave house’; ‘only able to get from bed to chair’; ‘severe memory problems’; and ‘daily pain’, at baseline and over the course of a two year follow-up period [42]. They found that participants were more likely to find the health states relating to functional ability (i.e. ‘unable to leave house’ and ‘only able to get from bed to chair’) as *acceptable* at baseline and throughout the study, whereas ‘severe memory problems’ and ‘daily pain’ were more likely to be rated as *unacceptable*, at baseline and throughout the study.

As part of a study to examine the influence of the risks and benefits of medications on treatment preferences of patients, Caughey et al. also examined the priorities of patients in the face of “competing outcomes” [48]. They found that 80% of participants would not be willing to take medication to reduce “joint pain”, if the medication increased their risk of a myocardial infarction by 10%. However, this was deduced from a sample of only 15 patients [48].

Agreement between patients and clinicians

Five of the included studies investigated the level of agreement in priority rankings between patients and their clinicians [45, 53-55, 59]. Three studies reported a low level of agreement between patient and clinicians’ priority rankings [53-55]. Two of these studies used a Cohen’s Kappa calculation to estimate the degree of agreement between the importance ratings of patients and clinicians, and the values of which were 0.18 and 0.11 respectively, indicating “slight agreement” after allowing for chance [54][55]. One study used a weighted kappa calculation to measure the degree of agreement, which, at a pre-intervention point in this study, was low at 6% [53].

Two studies reported that there was a “high” level of agreement [45, 59]. Deruaz-Luyet et al. found that in the case of 54.9% (n=314) of their patients, the condition that their GP had considered to be either the first or second most important, was in the same disease-group as the condition that the patient considered to be most important [59].

Zulman et al. reported that 60% of “patient-provider pairs” had a “high concordance”, meaning that the same three conditions had been rated as top three priorities by both parties, or that two of the same conditions had been rated in the top three priorities by both parties [45]. In this case, given that the sample of patients were all diabetic and hypertensive could have led to a narrowing of the range of chronic diseases across the sample, which in turn could have led to an increased likelihood of agreement. However, the participant characteristics reported by the authors state that the

For peer review only - http://bmjopen.bmj.com/site/about/guidelines.xhtml

patients had a mean of eight health conditions (SD 3.00), suggesting that the patients did not have a narrow range of chronic diseases.

QUALITATIVE SYNTHESIS

Whilst our quantitative synthesis allowed us to investigate *which* health outcomes, diseases or treatments were important to multi-morbid patients and their clinicians, our qualitative analysis enabled us to explore *how* prioritisation occurs. Below, we describe the key findings from our qualitative analysis.

Mechanisms of prioritisation

In the qualitative studies that approached prioritisation from a disease-specific perspective, patients were able to identify an illness as their main priority [50, 51]. For many patients, prioritisation appeared to be driven by their experience of the illness, which formed part of its "meaning as consequence" [51] as phrased by Cheraghi-Sohi et al. The 'consequences' of an illness consisted of the *impact* that the illness was having on the patients' everyday lives, which included functional limitation and the symptomatic burden of the illness, including its "unpredictability" (Table 3) [50]. For others, prioritisation appeared to be driven by their perception of the risk now and in the future with respect to functional deterioration and mortality.

In other studies, patients framed their priorities between *quality of life vs length of life* (Table 3) [43]. Patients in the study by Naik et al. who were multi-morbid adults with cancer, prioritised "quality of life" more highly than "length of life" [43]. This was also reflected in the findings of Fried et al., who found that when considering medication with competing outcomes in terms of extending life compared to quality of life, participants appeared to prioritise preserving quality of life [41].

Van Summeren et al. found that prioritisation was "difficult" when there was no "specific need" for a treatment decision to be made [46]. This concept of a difference in prioritisation based on hypothetical, or experiential levels, was also shared in the findings of Elliott et al [44] and Fried et al [41].

Where clinicians' perspectives were explored alongside patients, clinicians reported that exploring patients' priorities was "extremely important" when managing "competing interests" [48] and beneficial in providing patient-centred care [46]. Some clinicians in the mixed-methods study carried out by Van Summeren et al. reported that exploring their patients' priorities allowed them to have a "deeper understanding" of the patient, helped with making patient-centred treatment decisions and advance care planning (Table 3) [46]. However, other clinicians in the same study found exploring patient priorities to be difficult due its "novelty" and the fact that it represented a change to their usual consultations [46].

	Concept	Examples from included studies
Mechanisms of prioritisation	Unpredictability of symptoms	"My final issue is diverticulitis. In many ways that is the thing that makes the most impact on my life because of the unreliability of it. You make plans to do something to go somewhere and at the last minute you don't dare leave the house because you don't leave the loo. In itself it's not an important medical issue. It's the social problem more than anything else." – Lindsay et al [50]
	Quality of life vs length of life	"If you don't feel good, you can't take care of yourself and you have to depend on somebody else, what's the good of living another 10 years?"- Fried et al [41]

For peer review only - http://bmjopen.bmj.com/site/about/guidelines.xhtml

	Facilitating clinicians' decision making	"In future, I'll be happier to be more decisive in keeping an eye on what we do and do not do as regards this patient." Van Summeren et al [46]

Table 3- Examples from included studies for key concepts relating to mechanisms of prioritisation

Factors influencing prioritisation:

Our analysis revealed that there were a number of factors that appeared to influence how both patients and clinicians arrived at their priorities, and which priorities they chose.

i. **Functional ability**

Preserving functional ability as a priority for patients was a dominant concept across the majority of the qualitative studies [37, 38, 50, 52] [43][48][41]. Preserving independence emerged as the most significant reason for prioritising functional ability for patients, and maintaining the ability to engage in activities of daily living, mobility, maintaining cognitive ability and wanting to avoid being a "burden" or lacking social support to help them cope with functional deterioration (Table 4) [38, 50, 51].

Conditions which caused limitation to patients' ability to self-manage their health conditions, led to a "tension" between the patients' expectations of themselves and what they were physically able to do [52]. Lifestyle management, particularly reduced ability to exercise and the adverse impact of this on weight, was cited as part of patients' ability to self-manage [50].

Maintaining patients' functional ability was reported as a priority by some clinicians [37] [48]. Clinicians considered the wider implications of the patients' functional deterioration, particularly cognitive deterioration, and spoke of wanting to reduce the risk of "burnout" for the patients' family members/caregivers [37].

ii. **Mortality**

Reducing the risk of mortality emerged as a recurrent priority for clinicians [48, 56]. Caughey et al found that clinicians prioritised mortality in younger (less than 65 years) multi-morbid patients rather than older multi-morbid patients, as they felt they could be more "aggressive" in their treatment [48]. Reducing the risk of mortality also emerged as a priority for patients across a number of studies [37, 38, 44, 51, 52] [43]. Some patients found the asymptomatic nature of hypertension to be concerning; hence, the consequences of hypertension could be unpredictable, compared to some other chronic illnesses where symptoms can give warning of onset and severity (Table 4) [38, 44].

iii. **Symptom control**

The symptomatic burden of a condition contributed to its "meaning as consequence" for patients [51]. Symptoms were cited as being a cause of functional limitation [38, 50], and in some cases their "unpredictability" could cause significant disruption to patients' daily lives [50]. Symptom control was reported to be a priority by some clinicians [37][48]. However, clinicians in one study considered symptom control to be less important, particularly when there was no risk of mortality [56]. In these cases, clinicians seemed to be aware that patients may still be prioritising symptom control highly, even if the clinicians did not (Table 4).

iv. **Treatment burden**

For peer review only - http://bmjopen.bmj.com/site/about/guidelines.xhtml

Factors related to the treatment burden of an illness appeared to adversely impact prioritisation for patients, leading to *de-prioritisation* of certain medications and treatments [38, 41, 44, 49]. Elliot et al. reported that cost and distressing side effects, were factors which led patients to stop taking a medication [44]. Similarly, Fried et al. found that patients reported unpleasant side effects to be a "competing outcome", which negatively influenced their decision regarding continuing a medication [41]. However, difficulty with achieving control over the management of an illness, as well as requirement for high levels of engagement with self-management, emerged as factors that contributed to the prioritisation of an illness by some patients (Table 4) [50].

	Concept	Examples from included studies
Factors influencing prioritisation	Functional ability	"I mean, because I have to be mobile, I am living on my own, no one is going to take care of me, I have got to look after myself.." Cheraghi-Sohi et al [51]
	Mortality	"Well I really do worry the most about the high blood pressure. 'Cause see you know you got arthritis and you can tell when it's coming on. But you can't hardly tell about high blood pressure. It can just hit you like that [snaps fingers] ….'" Lindsay et al [50]
	Symptom control	"I would not want to live with pain. I won't allow that to happen"- Naik et al [43]
	Disparity in prioritisation of symptom control	".. I talk [to her] for a quarter of an hour about this and that every time after which she replies, "but my vertigo," and I answer every time, well, unfortunately there is nothing I can do about it, we have already tried and done everything. But it is probably the first diagnosis she will mention: "What are you suffering from?". "Vertigo". For me, this would be somewhere all the way at the bottom." – Hansen et al [56]
	Treatment burden	"It's the knee that's the most concerning because everything else is controlled by tablets. The knee is a problem because if I have one little slip I'm in plaster again for 6 weeks." Lindsay et al [50]

Table 4- Examples from included studies for key concepts relating to factors influencing prioritisation

DISCUSSION

Health outcome and treatment priorities

From our findings, patients' prioritisation appeared to be driven by weighing up the empirical compared to the hypothetical impact of a disease, whereby the empirical impact of a disease, which included its impact on function, symptomatic and treatment burden, was the most dominant driver of prioritisation. This is consistent with the findings of previous literature showing patients with rheumatoid arthritis who had reported experiencing higher levels of pain, were more likely to report pain as a priority [69].

Amongst empirical factors, preserving functionality emerged as most highly prioritised by patients amongst the quantitative studies that took a health outcome approach[39, 40, 47], whilst "function" was a domain that was prioritised highly by both patients and clinicians in the studies where prioritisation of various health *problems* were investigated [53-55]. From our qualitative findings, functional ability formed a key part of the preservation of various aspects of the patients'

For peer review only - http://bmjopen.bmj.com/site/about/guidelines.xhtml

independence and their quality of life, as well as their ability to self-manage. Existing evidence shows that the prevalence of multi-morbidity is highest in those aged over 65 years [70], and the population for the majority of the included studies were older multi-morbid adults. This could provide an explanation for why preserving functionality was highly prioritised.

Prioritisation was not a static process and was subject to change, based on factors such as illness exacerbations, life events, whether there was a need for a treatment decision to be made, and whether the priority related to retrospective or prospective healthcare [50, 52]. When considering the hypothetical impact of an illness, perceptions of future risk came into play, and in particular, the risk of mortality [44]. This was particularly evident in relation to cardiovascular disease, where patients appeared to perceive the risk of mortality to be high [38].

Risk of mortality was a dominant driver for prioritisation amongst clinicians. This was shown in our quantitative synthesis, where amongst studies assessing disease-specific priorities, conditions with a higher risk of mortality, such as cardiovascular disease and diabetes, recurrently emerged as being highly prioritised by clinicians [36, 45, 57] and differentiated by age [48]. This age-based consideration could explain why clinicians prioritised “quality of life for the patient” as higher, albeit marginally, than “life expectancy of the patient” in their clinical decision-making for de-prescribing for elderly, multi-morbid patients [58].

Our findings show a varying degree of agreement between the priorities of multi-morbid patients and clinicians. Previous studies carried out in the context of diabetes[71], and psoriasis[72] have found a low level of agreement on health outcome and treatment priorities between patients and clinicians, which correlates with the findings of some studies included in this review [53-55], but not others [45]. The nature of the patients’ illnesses emerged as a factor for concordance or discordance of priorities with their clinicians [37]. Patients and clinicians were in agreement in situations where patients were currently experiencing an exacerbation of a particular condition, or had a “stable” state of health. However, in patients who suffered from illnesses with more complex courses, discordance of priorities tended to occur between patients and clinicians [37].

Strengths and limitations

To our knowledge, this is the first systematic review to assimilate and compare the findings of existing literature on the health outcome and treatment priorities of both patients and clinicians for patients living with multi-morbidities. In this review, we have been able to add a novel line of argument to the ongoing discussion on this subject. By incorporating papers using both qualitative and quantitative methodologies, we have been able to explore patterns and relationships in the findings of a wide range of studies, thus creating a comprehensive and well-rounded systematic review.

There are noteworthy limitations. We did not include the term “comorbidity”, in our search terms, and whilst “comorbidity” is distinctive from multi-morbidity, there is also some conceptual overlap between the two terms. We felt that including “comorbidity” in our search strategy would identify studies focusing on a specific condition rather than multi-morbidity.

A number of the quantitative studies did not use pre-validated tools to ascertain priorities [36, 55, 57-59], leading to a risk of measurement bias, which could limit the generalisability of findings in this review. We also detected a large variation in the tools used to ascertain priorities, which meant that carrying out a meta-analysis to synthesise the findings of the quantitative studies was not possible. Yet, we have tried to mitigate the lack of meta-analysis by using a well-described and well-established method of narrative synthesis [33], in order to maintain rigour and transparency.

For peer review only - http://bmjopen.bmj.com/site/about/guidelines.xhtml

Recommendations for the future

We recommend that future guidelines developed for clinicians in the management of multi-morbidity highlight the need to elicit and consider both short term and long term priorities for their patients', and review these priorities continually, and particularly when exacerbations, changes to illness course or treatment regimens, or other wider socially-contextualised changes occur in their patients' lives.

There was a large variation in how priorities were ascertained, and in the tools used to ascertain priorities. The relative lack of standardised and validated tools for use to ascertain patient priorities in everyday clinical practice has also been described in previous literature [73]. We highlight a need for the development of a standardised and validated tool that is acceptable to both patients and clinicians, and can be used to ascertain patient-priorities in the multiple dimensions described in this review. Such a tool would a valuable aid to treatment decision-making, advance care planning and achieving patient-centeredness for patients living with multi-morbidity.

Conclusion

The findings from this review show the priorities of patients and clinicians can have varying degrees of concordance, being mostly low [53, 55], in alignment with previous findings in single disease contexts [71, 72]. We have found that the mechanisms of prioritisation can also differ between our two groups, in that patients are driven by illness experiences, whereas clinicians may be focused on managing longer term risks. Understanding these differences can help clinicians to better recognise situations where the patients' priorities may be different to theirs and elicit the most important priorities for their patients.

For peer review only

For peer review only - http://bmjopen.bmj.com/site/about/guidelines.xhtml

REFERENCES

1 van den Akker M, Buntinx F, Knottnerus JA. Comorbidity or multimorbidity: what's in a name? A review of literature, *The European Journal of General Practice* 1996;2:65-70.

2 Garin N, Koyanagi A, Chatterji S, et al. Global multimorbidity patterns: a cross-sectional, population-based, multi-country study, *Journals of Gerontology Series A: Biomedical Sciences and Medical Sciences* 2015;71:205-14.

3 Rijken M, Struckmann V, Dyakova M, et al. ICARE4EU: Improving care for people with multiple chronic conditions in Europe. 2013.

4 Roberts KC, Rao DP, Bennett TL, et al. Prevalence and patterns of chronic disease multimorbidity and associated determinants in Canada, *Health Promot Chronic Dis Prev Can* 2015;35:87-94.

5 Rocca WA, Boyd CM, Grossardt BR, et al. Prevalence of multimorbidity in a geographically defined American population: patterns by age, sex, and race/ethnicity. 2014;89:1336-49.

6 Arokiasamy P, Uttamacharya U, Jain K, et al. The impact of multimorbidity on adult physical and mental health in low-and middle-income countries: what does the study on global ageing and adult health (SAGE) reveal? *BMC medicine* 2015;13:178.

7 Afshar S, Roderick PJ, Kowal P, et al. Multimorbidity and the inequalities of global ageing: a cross-sectional study of 28 countries using the World Health Surveys, *BMC Public Health* 2015;15:776.

8 Tinetti ME, Bogardus Jr ST, Agostini JV. Potential pitfalls of disease-specific guidelines for patients with multiple conditions, *N Engl J Med* 2004;351:2870-4.

9 Calderon-Larranaga A, Poblador-Plou B, Gonzalez-Rubio F, et al. Multimorbidity, polypharmacy, referrals, and adverse drug events: are we doing things well? *Br J Gen Pract* 2012;62:e821-6.

10 Fortin M, Bravo G, Hudon C, et al. Relationship between multimorbidity and health-related quality of life of patients in primary care, *Quality of Life Research* 2006;15:83-91.

11 Marengoni A, Angleman S, Melis R, et al. Aging with multimorbidity: a systematic review of the literature, *Ageing research reviews* 2011;10:430-9.

12 Fortin M, Bravo G, Hudon C, et al. Psychological distress and multimorbidity in primary care, *Ann Fam Med* 2006;4:417-22.

13 Academy of Medical Sciences. Multimorbidity: a priority for global health research, 2018.

14 Muth C, van den Akker M, Blom JW, et al. The Ariadne principles: how to handle multimorbidity in primary care consultations. *BMC Medicine* 2014;12:223.

For peer review only - http://bmjopen.bmj.com/site/about/guidelines.xhtml

15 Azad NA, Mielniczuk L. A call for collaboration: improving cardiogeriatric care, *Can J Cardiol* 2016;32:1041-4.

16 Roland M, Paddison C. Better management of patients with multimorbidity, *BMJ: British Medical Journal (Online)* 2013;346.

17 Bierman AS, Tinetti ME. Precision medicine to precision care: managing multimorbidity, *Lancet* 2016;388:2721-3.

18 Mc Namara KP, Breken BD, Alzubaidi HT, et al. Health professional perspectives on the management of multimorbidity and polypharmacy for older patients in Australia, *Age & Ageing* 2017;46:291-9.

19 Sinnott C, Mc Hugh S, Browne J, et al. GPs' perspectives on the management of patients with multimorbidity: systematic review and synthesis of qualitative research, *BMJ Open* 2013;3:e003610,2013-003610.

20 Rothwell PM, McDowell Z, Wong CK, et al. Doctors and patients don't agree: cross sectional study of patients' and doctors' perceptions and assessments of disability in multiple sclerosis, *BMJ* 1997;314:1580-3.

21 Montgomery AA, Fahey T. How do patients' treatment preferences compare with those of clinicians? *Qual Health Care* 2001;10 Suppl 1:i39-43.

22 Thomson S, Doody G. Parallel paths? Patient and doctor priorities in psychiatric outpatient consultations, *Journal of Mental Health* 2010;19:461-9.

23 Lee CN, Hultman CS, Sepucha K. Do patients and providers agree about the most important facts and goals for breast reconstruction decisions? *Ann Plast Surg* 2010;64:563-6.

24 Volandes AE, Paasche-Orlow MK, Barry MJ, et al. Video decision support tool for advance care planning in dementia: randomised controlled trial, *BMJ* 2009;338:b2159.

25 Pager CK, McCluskey PJ. Surgeons' perceptions of their patients' priorities, *Journal of Cataract & Refractive Surgery* 2004;30:591-7.

26 Higgins JP, Green S. Cochrane handbook for systematic reviews of interventions: John Wiley & Sons 2011.

27 Moher D, Shamseer L, Clarke M, et al. Preferred reporting items for systematic review and meta-analysis protocols (PRISMA-P) 2015 statement, *Systematic reviews* 2015;4:1.

28 Moher D, Liberati A, Tetzlaff J, et al. Preferred reporting items for systematic reviews and meta-analyses: the PRISMA statement, *Ann Intern Med* 2009;151:264-9.

29 Downes MJ, Brennan ML, Williams HC, et al. Development of a critical appraisal tool to assess the quality of cross-sectional studies (AXIS), *BMJ Open* 2016;6:e011458,2016-011458.

30 Stang A. Critical evaluation of the Newcastle-Ottawa scale for the assessment of the quality of nonrandomized studies in meta-analyses, *Eur J Epidemiol* 2010;25:603-5.

For peer review only

For peer review only - http://bmjopen.bmj.com/site/about/guidelines.xhtml

31 Higgins JP, Altman DG, Gotzsche PC, et al. The Cochrane Collaboration's tool for assessing risk of bias in randomised trials, *BMJ* 2011;343:d5928.

32 Critical Appraisal Skills Programme UK. CASP qualitative research checklist. *CASP checklists* 13/03/2017.

33 Popay J, Roberts H, Sowden A, et al. Guidance on the conduct of narrative synthesis in systematic reviews, *A product from the ESRC methods programme Version* 2006;1:b92.

34 Noblit GW, Hare RD. Meta-ethnography: Synthesizing qualitative studies: sage 1988.

35 Britten N, Campbell R, Pope C, et al. Using meta ethnography to synthesise qualitative research: a worked example, *J Health Serv Res Policy* 2002;7:209-15.

36 Moore A., Patterson C., Nair K., et al. Minding the gap: Prioritization of care issues among nurse practitioners, family physicians and geriatricians when caring for the elderly. *Journal of interprofessional care* 2015;29:401-3.

37 Kuluski K, Gill A, Naganathan G, et al. A qualitative descriptive study on the alignment of care goals between older persons with multi-morbidities, their family physicians and informal caregivers. *BMC Family Practice* 2013;14:133.

38 Schoenberg NE, Leach C, Edwards W. "It's a toss up between my hearing, my heart, and my hip": prioritizing and accommodating multiple morbidities by vulnerable older adults, *J Health Care Poor Underserved* 2009;20:134-51.

39 Fried TR, Tinetti ME, Iannone L, et al. Health outcome prioritization as a tool for decision making among older persons with multiple chronic conditions, *Arch Intern Med* 2011;171:1856-8.

40 Fried TR, Tinetti M, Agostini J, et al. Health outcome prioritization to elicit preferences of older persons with multiple health conditions, *Patient Educ Couns* 2011;83:278-82.

41 Fried TR, McGraw S, Agostini JV, et al. Views of older persons with multiple morbidities on competing outcomes and clinical decision-making, *J Am Geriatr Soc* 2008;56:1839-44.

42 Fried TR, Byers AL, Gallo WT, et al. Prospective study of health status preferences and changes in preferences over time in older adults, *Arch Intern Med* 2006;166:890-5.

43 Naik A.D., Martin L.A., Moye J., et al. Health Values and Treatment Goals of Older, Multimorbid Adults Facing Life-Threatening Illness. *J Am Geriatr Soc* 2016;64:625-31.

44 Elliott RA, Ross-Degnan D, Adams AS, et al. Strategies for coping in a complex world: adherence behavior among older adults with chronic illness, *Journal of General Internal Medicine* 2007;22:805-10.

45 Zulman D.M., Kerr E.A., Hofer T.P., et al. Patient-provider concordance in the prioritization of health conditions among hypertensive diabetes patients. *Journal of General Internal Medicine* 2010;25:408-14.

46 Summeren JJ, Haaijer-Ruskamp FM, Schuling J. Eliciting preferences of multimorbid elderly adults in family practice using an outcome prioritization tool, *J Am Geriatr Soc* 2016;64.

For peer review only

For peer review only - http://bmjopen.bmj.com/site/about/guidelines.xhtml

47 van Summeren JJ, Schuling J, Haaijer-Ruskamp FM, et al. Outcome prioritisation tool for medication review in older patients with multimorbidity: a pilot study in general practice, *Br J Gen Pract* 2017;67:e501-6.

48 Caughey G.E., Tait K., Vitry A.I., et al. Influence of medication risks and benefits on treatment preferences in older patients with multimorbidity. *Patient Preference and Adherence* 2017;11:131-40.

49 Turner J.P., Edwards S., Stanners M., et al. What factors are important for deprescribing in Australian long-term care facilities? Perspectives of residents and health professionals. *BMJ Open* 2016;6:Arte Number: e009781. ate of Pubaton: 2016.

50 LINDSAY S. PRIORITIZING ILLNESS: LESSONS IN SELFa MANAGING MULTIPLE CHRONIC DISEASES, *CANADIAN JOURNAL OF SOCIOLOGY/CAHIERS CANADIENS DE SOCIOLOGIE* 2009;34:983.

51 Cheraghi-Sohi S, Morden A, Bower P, et al. Exploring patient priorities among long-term conditions in multimorbidity: A qualitative secondary analysis. *SAGE Open Medicine* 2013;1:2050312113503955.

52 Morris R.L., Sanders C., Kennedy A.P., et al. Shifting priorities in multimorbidity: A longitudinal qualitative study of patient's prioritization of multiple conditions. *Chronic Illness* 2011;7:147-61.

53 Junius-Walker U, Wrede J, Voigt I, et al. Impact of a priority-setting consultation on doctor-patient agreement after a geriatric assessment: cluster randomised controlled trial in German general practices. *Quality in primary care* 2012;20.

54 Junius-Walker U, Stolberg D, Steinke P, et al. Health and treatment priorities of older patients and their general practitioners: a cross-sectional study. *Quality in primary care* 2011;19.

55 Voigt I, Wrede J, Diederichs-Egidi H, et al. Priority setting in general practice: health priorities of older patients differ from treatment priorities of their physicians, *Croat Med J* 2010;51:483-92.

56 Hansen H., Pohontsch N., van den Bussche H., et al. Reasons for disagreement regarding illnesses between older patients with multimorbidity and their GPs - a qualitative study. *BMC family practice* 2015;16:68.

57 Herzig L, Mueller Y, Haller DM, et al. Family practitioners' top medical priorities when managing patients with multimorbidity: a cross-sectional study, *BJGP open* 2019;3:bjgpopen18X101622.

58 Mantelli S, Jungo KT, Rozsnyai Z, et al. How general practitioners would deprescribe in frail oldest-old with polypharmacy—the LESS study, *BMC family practice* 2018;19:169.

59 Déruaz-Luyet A, N'Goran AA, Pasquier J, et al. Multimorbidity: can general practitioners identify the health conditions most important to their patients? Results from a national cross-sectional study in Switzerland, *BMC family practice* 2018;19:66.

60 Tinetti ME, McAvay GJ, Fried TR, et al. Health outcome priorities among competing cardiovascular, fall injury, and medication-related symptom outcomes, *J Am Geriatr Soc* 2008;56:1409-16.

For peer review only

61 DeruazLuyet A., Alexandra N'Goran A., Tandjung R., et al. Multimorbidity in primary care: Protocol of a national cross-sectional study in Switzerland. *BMJ Open* 2015;5:Arte Number: e009165. ate of Pubaton: 2015.

62 Martin LA, Moye J, Street Jr RL, et al. Reconceptualizing cancer survivorship through veterans' lived experiences, *J Psychosoc Oncol* 2014;32:289-309.

63 Lindsay S, Bellaby P, Smith S, et al. Enabling healthy choices: is ICT the highway to health improvement? *Health:* 2008;12:313-31.

64 Hurley MV, Walsh N, Bhavnani V, et al. Health beliefs before and after participation on an exercised-based rehabilitation programme for chronic knee pain: doing is believing, *BMC Musculoskeletal Disorders* 2010;11:31.

65 Bower P, Harkness E, Macdonald W, et al. Illness representations in patients with multimorbid long-term conditions: Qualitative study, *Psychol Health* 2012;27:1211-26.

66 Grime J, Richardson JC, Ong BN. Perceptions of joint pain and feeling well in older people who reported being healthy: a qualitative study, *Br J Gen Pract* 2010;60:597-603.

67 Nio Ong B, Jinks C, Morden A. The hard work of self-management: Living with chronic knee pain, *International journal of qualitative studies on health and well-being* 2011;6:7035.

68 Hansen H., Schafer I., Schon G., et al. Agreement between self-reported and general practitioner-reported chronic conditions among multimorbid patients in primary care - results of the MultiCare Cohort Study. *BMC family practice* 2014;15:39.

69 Heiberg T, Kvien TK. Preferences for improved health examined in 1,024 patients with rheumatoid arthritis: pain has highest priority, *Arthritis Care & Research* 2002;47:391-7.

70 Barnett K, Mercer SW, Norbury M, et al. Epidemiology of multimorbidity and implications for health care, research, and medical education: a cross-sectional study, *The Lancet* 2012;380:37-43.

71 Heisler M, Vijan S, Anderson RM, et al. When do patients and their physicians agree on diabetes treatment goals and strategies, and what difference does it make? *Journal of general internal medicine* 2003;18:893-902.

72 Okubo Y, Tsuruta D, Tang A, et al. Analysis of treatment goal alignment between Japanese psoriasis patients and their paired treating physicians, *Journal of the European Academy of Dermatology and Venereology* 2018;32:606-14.

73 Mangin D., Stephen G., Bismah V., et al. Making patient values visible in healthcare: A systematic review of tools to assess patient treatment priorities and preferences in the context of multimorbidity. *BMJ Open* 2016;6:Arte Number: e010903. ate of Pubaton: 01 Jun 2016.

For peer review only

For peer review only - http://bmjopen.bmj.com/site/about/guidelines.xhtml

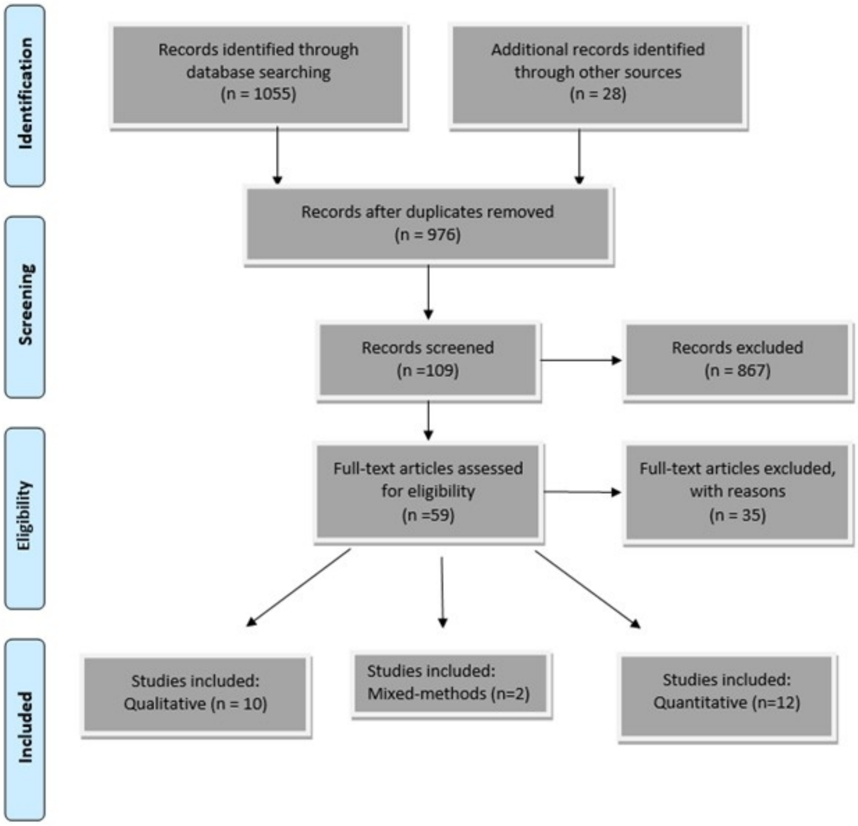

Figure 1 Flow diagram to illustrate process from literature searching to selection of studies for inclusion [28]

121x111mm (150 x 150 DPI)

**1. Patient*.mp.**
**2. Patients/**
**3. 1 or 2**
**4. Priorit*.mp. [mp=title, abstract, original title, name of substance word, subject heading word, keyword heading word, protocol supplementary concept word, rare disease supplementary concept word, unique identifier, synonyms]**
**5. Choice*.mp.**
**6. Preference*.mp.**
**7. Aim*.mp.**
**8. Goal*.mp.**
**9. 4 or 5 or 6 or 7 or 8**
**10. Doctor*.mp.**
**11. Physicians/**
**12. Clinician*.mp.**
**13. Primary Health Care/ or Physicians, Family/ or Family Practice/ or General Practitioners/**
**14. General practitioner*.mp.**
**15. 10 or 11 or 12 or 13 or 14**
**16. Multimorbidit*.mp. [mp=title, abstract, original title, name of substance word, subject heading word, keyword heading word, protocol supplementary concept word, rare disease supplementary concept word, unique identifier, synonyms]**
**17. Multi-morbidit*.mp. [mp=title, abstract, original title, name of substance word, subject heading word, keyword heading word, protocol supplementary concept word, rare disease supplementary concept word, unique identifier, synonyms]**
**18. Multiple morbidit*.mp. [mp=title, abstract, original title, name of substance word, subject heading word, keyword heading word, protocol supplementary concept word, rare disease supplementary concept word, unique identifier, synonyms]**
**19. 16 or 17 or 18**
**20. 3 and 9 and 15 and 19**
**21. Multi morbid*.mp. [mp=title, abstract, original title, name of substance word, subject heading word, keyword heading word, protocol supplementary concept word, rare disease supplementary concept word, unique identifier, synonyms]**
**22. 16 or 17 or 18 or 21**
**23. 3 and 9 and 15 and 22**

Appendix 1

For peer review only

CASP checklist for qualitative research summary table

	**Kuluski et al** [1]	**Schoenberg et al** [2]	**Cheraghi-Sohi et al** [3]	**Naik et al** [4]	**Lindsay et al** [5]	**Hansen et al** [6]	**Morris et al** [7]	**Elliott et al** [8]	**Fried et al** [9]	**Turner et al** [10]	**Van Summeren et al** [11]	**Caughey et al** [12]
Was there a clear statement of the aims of the research?	YES	YES	YES	YES	YES	YES	YES	YES	YES	YES	YES	YES
Is a qualitative methodology appropriate?	YES	YES	YES	YES	YES	YES	YES	YES	YES	NO- Quantitative or mixed methods methodology would have been more appropriate as the aim was to rank factors, although data collected using a qualitative technique, it lacks richness and appears to be presented in a quantitative manner	YES	YES
Was the research design appropriate to the aims of the research?	YES	YES	YES	YES	YES	YES	YES	YES	YES	YES	YES	YES
Was the recruitment strategy	YES	YES	YES	YES	YES	YES	NO- no explanation given as to	YES	YES	YES	YES	YES

For peer review only

appropriate to the aims of the research							why the specific conditions were chosen (COPD, IBS etc)					
Were the data collected in a way that addressed the research issue?	YES	YES	YES	YES	YES	YES	YES	YES	YES	YES	YES	YES
Has the relationship between researcher and participants been adequately considered?	YES	YES	YES	NO- no information given on background of main researcher and no consideration given to possibility of researcher bias at any point.	NO- There is no background information given on the researcher (sole in this case) and there has been no evidence of any consideration of researcher bias at any point during the study.	NO- there has been no evidence of any consideration of researcher bias at any point during the study	NO- no information given on background of main researcher and no consideration given to possibility of researcher bias at any point. However there was some evidence of reflexivity during the data collection process when emerging areas of interest that could be incorporated into future interviews	NO- background of RAE who conducted interviews and main aspect of analysis not specified and no consideration has been given to any possibility of researcher bias	NO- No explanatio n given of the profession al backgroun d of the researcher s or the moderator for the focus groups, and there has been no evidence of any considerati on of researcher bias at any point during the study.	NO- no mention of the background of the researchers or how this may have influenced the results	NO- role of second interviewer carrying out the in-depth interviews not mentioned, and there has been no consideration given to the possibility of bias from the interviewers. One of the interviewers was a FP, which could have led to bias with the interviewees responses.	NO- there has been no consideratio n given to the role of the researcher and the potential for researcher bias at any point.

							were considered.					
Have ethical issues been taken into consideration ?	YES	YES	YES- in the original studies, however further ethical issues regarding secondary qualitative analysis were not taken into account.	YES	YES	YES	YES	YES	YES	YES	YES	YES
Was the data analysis sufficiently rigorous?	YES	YES	YES	YES	NO- superficial description of analytic process and no information given on how many researchers analysed the transcripts- assumed one as there is only one author- risk of bias not taken into account for the analytic process	YES	YES	YES	YES	NO- the data analysis process is very ambiguous and the qualitative analysis has not been described in sufficient depth.	YES- clear description of the analytic process with two researchers independently analysing the data for rigour. However no description of the interpretation phase from the data.	NO- there is only a superficial description of the data analysis process, and there is very little detail given on how the themes were derived from the data. There is no presentation at all of quotes from the data to support the authors interpretation of the data.
Is there a clear	YES	YES	YES	YES	YES	YES	YES	YES	YES	YES	YES- however the qualitative	YES- however no quotes given

For peer review only

statement of findings?											data from the patient interviews has only been summarised- no direct quotes given	to support findings
How valuable is the research?	Valuable	Valuable	Valuable	Valuable	Valuable	Valuable	Valuable	Valuable	Valuable	Valuable	Valuable	Valuable

For peer review only

Newcastle-ottawa scale for cohort and observational studies summary table								
	Representativeness of the exposed cohort	Selection of the non-exposed cohort	Ascertainment of exposure	Demonstration that outcome of interest was not present at start of study	Comparability of cohorts on the basis of the design or analysis controlled for confounders	Assessment of outcome	Was follow-up long enough for outcomes to occur	Adequacy of follow-up of cohorts
Zulman et al [13]	Somewhat representative (one star) *	Drawn from the same community as the exposed cohort (one star) *	Secure record (one star) *	N/A	The study controls for age, sex and marital status (one star)*	Self-report	N/A	No statement
Fried et al [14]	Somewhat representative (one star) *	N/A	Secure record (one star) *	N/A	Cohorts are not comparable on the basis of the design	Self-report	Yes (one star) *	Follow up rate less than 80%

For peer review only

Axis tool for cross-sectional studies summary table

**Introduction**		Junius-Walker et al [15]	Fried et al[16]	Fried et al [17]	Moore et al [18]	Van Summeren et al [19]	Voigt et al [20]	Van Summeren et al [11]	Caughey et al [21]	Mantelli et al [22]	Deruaz-Luyet et al [23]	Herzig et al [24]
1	Were the aims/objectives of the study clear?	Yes	Yes	Yes	Yes	Yes	Yes	Yes	Yes	Yes	Yes	Yes
Methods												
2	Was the study design appropriate for the stated aim(s)?	Yes	Yes	Yes	Yes	Yes	Yes	Yes	Yes	Yes	Yes	Yes
3	Was the sample size justified?	No- convenience sampling used, small sample size, however no explanation for sample size given	No- no justification for sample size given, convenience sampling used	No- recruitment strategy described clearly but no justification for sample size given	Yes	No	No- sampling strategy described well but no justification for sample size given	No- purposive sampling used, however no justification for sample size given	No- no justification for sample size given	No- convenience sampling used and no justification for sample size given	Yes- in the parent study [25]	Yes- in the parent study [25]
4	Was the target/reference population clearly defined? (Is it clear who the research was about?)	Yes	Yes	Yes	Yes	Yes	Yes	Yes	Yes	Yes	Yes	Yes
5	Was the sample frame taken from an appropriate population base so that it closely represented the target/reference population under investigation?	Yes	Yes- However assumption made that participants will have multiple chronic conditions	Yes	Yes	Yes	Yes	Yes	Yes	Yes- although only GP's who had previously taken part in other case-vignette studies were invited, leading to possibility of selection bias	Yes	Yes

For peer review only

6	Was the selection process likely to select subjects/participants that were representative of the target/reference population under investigation?	Yes	Yes- as above	Yes	Yes	Yes	Yes	Yes	Yes	Yes- as above	Yes	Yes
7	Were measures undertaken to address and categorise non-responders?	Yes	Don't know- not reported	Yes	No	No	No	Yes- Purposive sampling used with efforts made to address gaps in participant types	Don't know- not reported	Don't know- not reported	Yes in the parent study [26].<br><br>Characteristics of participants who were not included due to missing data, were described in this study	Yes in the parent study [26]
8	Were the risk factor and outcome variables measured appropriate to the aims of the study?	Yes	Yes	Yes	Yes	Yes	Yes	Yes	Yes	Yes	Yes	Yes
9	Were the risk factor and outcome variables measured correctly using instruments/measurements that had been trialled, piloted or published previously?	Yes	Yes- piloted in a previous study	No- Tested in this study as it was a feasibility study	No- Pre-tested in this study but only using 2 FP's and 1 NP	Yes	No- STEP assessment previously published however no testing done of measure used to collect importance ratings	Yes	Yes	Yes- the instruments used were piloted within this study using 5 GP's as participants, but had not been published previously	No- instruments designed through "internal consensus discussions".	No

For peer review only

10	Is it clear what was used to determined statistical significance and/or precision estimates? (e.g. p-values, confidence intervals)	Yes	N/A	Yes	Yes	N/A	Yes	N/A	N/A	Yes	Yes	Yes
11	Were the methods (including statistical methods) sufficiently described to enable them to be repeated?	Yes	Yes	Yes	Yes	Yes	Yes	Yes	Yes	Yes	Yes	Yes
Results												
12	Were the basic data adequately described?	Yes	Yes	Yes	Yes	No- No reporting of prioritisation of patients for whom no medication changes were proposed	Yes	Yes	Yes	Yes	Yes	Yes
13	Does the response rate raise concerns about non-response bias?	No	Don't know-response rate not reported	No	No	No	Don't know-response rate not reported	No	Don't know-response rate not reported	No	No	No
14	If appropriate, was information about non-responders described?	Yes	No	Yes	No	Yes	No	Yes	No	No	Yes in the parent study[26]<br><br>Characteristics of participants who were not included	Yes in the parent study[26]

For peer review only

											due to missing data, were described in this study	
15	Were the results internally consistent?	Yes	Yes	Yes	Yes	Yes	Yes	Yes	Yes	Yes	Yes	Yes
16	Were the results presented for all the analyses described in the methods?	Yes	Yes	Yes	Yes	No- No reporting of prioritisation of patients for whom no medication changes were proposed	Yes	Yes	Yes	Yes	Yes	Yes
Discussion												
17	Were the authors' discussions and conclusions justified by the results?	No- very small sample of GP’s compared to patients therefore generalizable conclusions regarding concordance between doctors and patients cannot accurately be drawn from this study	Yes	Yes	Yes	Yes	Yes	Yes- Small sample size for quantitative aspect of study taken into account	No- very small sample size across patients and clinicians, meaning results are not generalizable	Yes	Yes	Yes

For peer review only

18	Were the limitations of the study discussed?	Yes	No	Yes	Yes	Yes	Yes	Yes	Yes	Yes	Yes	Yes
Other												
19	Were there any funding sources or conflicts of interest that may affect the authors' interpretation of the results?	No	No	No	No	No	No	No	No	No	No	No
20	Was ethical approval or consent of participants attained?	Yes	Yes	Yes	Yes	Yes	Yes	Yes	Yes	Yes	Yes	Yes

For peer review only

The Cochrane Collaboration's tool for assessing risk of bias in randomised controlled trials summary table		
Study	Junius-Walker et al [27]	
Bias	Authors' judgement	Support for judgement
Random sequence generation (selection bias)	Low risk	"Participating doctors were allocated 1:1 into the intervention and control group using random block sizes of four"
Allocation concealment (selection bias)	Unclear risk	No information given regarding any efforts to conceal the allocation sequence
Blinding of participants and researchers (performance bias)	Low risk	Participants were only informed of the procedures of their own trial arm.
Blinding of outcome assessment (detection bias)	Low risk	Participants were blinded to the pre-intervention importance ratings, when completing the final importance ratings.
Incomplete outcome data (attrition bias)	High risk	25 patients dropped out prior to baseline ratings and 5 further patients dropped out prior to final ratings, these patients were excluded from analysis, however intention to treat analysis cannot be carried out in this context due to the nature of the intervention
Selective reporting (reporting bias)	Low risk	Adequate reporting on all of the specified outcomes
Other bias	None detected	

For peer review only

References

1 Kuluski K, Gill A, Naganathan G, et al. A qualitative descriptive study on the alignment of care goals between older persons with multi-morbidities, their family physicians and informal caregivers. *BMC Family Practice* 2013;14:133.

2 Schoenberg NE, Leach C, Edwards W. "It's a toss up between my hearing, my heart, and my hip": prioritizing and accommodating multiple morbidities by vulnerable older adults, *J Health Care Poor Underserved* 2009;20:134-51.

3 Cheraghi-Sohi S, Morden A, Bower P, et al. Exploring patient priorities among long-term conditions in multimorbidity: A qualitative secondary analysis. *SAGE Open Medicine* 2013;1:2050312113503955.

4 Naik A.D., Martin L.A., Moye J., et al. Health Values and Treatment Goals of Older, Multimorbid Adults Facing Life-Threatening Illness. *J Am Geriatr Soc* 2016;64:625-31.

5 LINDSAY S. PRIORITIZING ILLNESS: LESSONS IN SELFa MANAGING MULTIPLE CHRONIC DISEASES, *CANADIAN JOURNAL OF SOCIOLOGY/CAHIERS CANADIENS DE SOCIOLOGIE* 2009;34:983.

6 Hansen H., Pohontsch N., van den Bussche H., et al. Reasons for disagreement regarding illnesses between older patients with multimorbidity and their GPs - a qualitative study. *BMC family practice* 2015;16:68.

7 Morris R.L., Sanders C., Kennedy A.P., et al. Shifting priorities in multimorbidity: A longitudinal qualitative study of patient's prioritization of multiple conditions. *Chronic Illness* 2011;7:147-61.

8 Elliott RA, Ross-Degnan D, Adams AS, et al. Strategies for coping in a complex world: adherence behavior among older adults with chronic illness, *Journal of General Internal Medicine* 2007;22:805-10.

9 Fried TR, McGraw S, Agostini JV, et al. Views of older persons with multiple morbidities on competing outcomes and clinical decision-making, *J Am Geriatr Soc* 2008;56:1839-44.

10 Turner J.P., Edwards S., Stanners M., et al. What factors are important for deprescribing in Australian long-term care facilities? Perspectives of residents and health professionals. *BMJ Open* 2016;6:Arte Number: e009781. ate of Pubaton: 2016.

11 Summeren JJ, Haaijer-Ruskamp FM, Schuling J. Eliciting preferences of multimorbid elderly adults in family practice using an outcome prioritization tool, *J Am Geriatr Soc* 2016;64.

12 Caughey G.E., Huynh E., Shakib S., et al. Influence of medication risks and benefits on patient and clinician preferences for treatment in multimorbidity: A discrete-choice experiment. 2017.

13 Zulman D.M., Kerr E.A., Hofer T.P., et al. Patient-provider concordance in the prioritization of health conditions among hypertensive diabetes patients. *Journal of General Internal Medicine* 2010;25:408-14.

14 Fried TR, Byers AL, Gallo WT, et al. Prospective study of health status preferences and changes in preferences over time in older adults, *Arch Intern Med* 2006;166:890-5.

15 Junius-Walker U, Stolberg D, Steinke P, et al. Health and treatment priorities of older patients and their general practitioners: a cross-sectional study. *Quality in primary care* 2011;19.

16 Fried TR, Tinetti ME, Iannone L, et al. Health outcome prioritization as a tool for decision making among older persons with multiple chronic conditions, *Arch Intern Med* 2011;171:1856-8.

17 Fried TR, Tinetti M, Agostini J, et al. Health outcome prioritization to elicit preferences of older persons with multiple health conditions, *Patient Educ Couns* 2011;83:278-82.

18 Moore A., Patterson C., Nair K., et al. Minding the gap: Prioritization of care issues among nurse practitioners, family physicians and geriatricians when caring for the elderly. *Journal of interprofessional care* 2015;29:401-3.

19 Van Summeren J.J.G.T., Schuling J., HaaijerRuskamp F.M., et al. Outcome prioritisation tool for medication review in older patients with multimorbidity: A pilot study in general practice. *British Journal of General Practice* 2017;67:e501-6.

20 Voigt I, Wrede J, Diederichs-Egidi H, et al. Priority setting in general practice: health priorities of older patients differ from treatment priorities of their physicians, *Croat Med J* 2010;51:483-92.

21 Caughey G.E., Tait K., Vitry A.I., et al. Influence of medication risks and benefits on treatment preferences in older patients with multimorbidity. *Patient Preference and Adherence* 2017;11:131-40.

For peer review only

22 Mantelli S, Jungo KT, Rozsnyai Z, et al. How general practitioners would deprescribe in frail oldest-old with polypharmacy—the LESS study, *BMC family practice* 2018;19:169.

23 Déruaz-Luyet A, N'Goran AA, Pasquier J, et al. Multimorbidity: can general practitioners identify the health conditions most important to their patients? Results from a national cross-sectional study in Switzerland, *BMC family practice* 2018;19:66.

24 Herzig L, Mueller Y, Haller DM, et al. Family practitioners' top medical priorities when managing patients with multimorbidity: a cross-sectional study, *BJGP open* 2019;3:bjgpopen18X101622.

25 DeruazLuyet A., Alexandra N'Goran A., Tandjung R., et al. Multimorbidity in primary care: Protocol of a national cross-sectional study in Switzerland. *BMJ Open* 2015;5:Arte Number: e009165. ate of Pubaton: 2015.

26 Deruaz-Luyet A, N'Goran AA, Senn N, et al. Multimorbidity and patterns of chronic conditions in a primary care population in Switzerland: a cross-sectional study, *BMJ Open* 2017;7:e013664,2016-013664.

27 Junius-Walker U, Wrede J, Voigt I, et al. Impact of a priority-setting consultation on doctor-patient agreement after a geriatric assessment: cluster randomised controlled trial in German general practices. *Quality in primary care* 2012;20.

For peer review only

Reporting checklist for systematic review and meta-analysis.

Based on the PRISMA guidelines.

For peer review only

		Reporting Item	Page Number
**Title**			
	#1	Identify the report as a systematic review, meta-analysis, or both.	1
**Abstract**			
Structured summary	#2	Provide a structured summary including, as applicable: background; objectives; data sources; study eligibility criteria, participants, and interventions; study appraisal and synthesis methods; results; limitations; conclusions and implications of key findings; systematic review registration number	2
**Introduction**			
Rationale	#3	Describe the rationale for the review in the context of what is already known.	3
Objectives	#4	Provide an explicit statement of questions being addressed with reference to participants,	3

		interventions, comparisons, outcomes, and study design (PICOS).	
**Methods**			
Protocol and registration	#5	Indicate if a review protocol exists, if and where it can be accessed (e.g., Web address) and, if available, provide registration information including the registration number.	3
Eligibility criteria	#6	Specify study characteristics (e.g., PICOS, length of follow-up) and report characteristics (e.g., years considered, language, publication status) used as criteria for eligibility, giving rational	3
Information sources	#7	Describe all information sources in the search (e.g., databases with dates of coverage, contact with study authors to identify additional studies) and date last searched.	3
Search	#8	Present full electronic search strategy for at least one database, including any limits used, such that it could be repeated.	3, Appendix 1
Study selection	#9	State the process for selecting studies (i.e., for screening, for determining eligibility, for inclusion in the systematic review, and, if applicable, for inclusion in the meta-analysis).	4, Figure 1

For peer review only

Data collection process	#10	Describe the method of data extraction from reports (e.g., piloted forms, independently by two reviewers) and any processes for obtaining and confirming data from investigators.	4
Data items	#11	List and define all variables for which data were sought (e.g., PICOS, funding sources), and any assumptions and simplifications made.	3
Risk of bias in individual studies	#12	Describe methods used for assessing risk of bias in individual studies (including specification of whether this was done at the study or outcome level, or both), and how this information is to be used in any data synthesis.	4
Summary measures	#13	State the principal summary measures (e.g., risk ratio, difference in means).	N/A
Planned methods of analyis	#14	Describe the methods of handling data and combining results of studies, if done, including measures of consistency (e.g., I2) for each meta-analysis.	4
Risk of bias across studies	#15	Specify any assessment of risk of bias that may affect the cumulative evidence (e.g., publication bias, selective reporting within studies).	4
Additional analyses	#16	Describe methods of additional analyses (e.g., sensitivity or subgroup analyses, meta-regression), if done, indicating which were pre-specified.	N/A

For peer review only

Results

Study selection	#17	Give numbers of studies screened, assessed for eligibility, and included in the review, with reasons for exclusions at each stage, ideally with a flow diagram.	Figure 1
Study characteristics	#18	For each study, present characteristics for which data were extracted (e.g., study size, PICOS, follow-up period) and provide the citation.	5,6,7,8,9,10 (Table 1)
Risk of bias within studies	#19	Present data on risk of bias of each study and, if available, any outcome-level assessment (see Item 12).	Appendix 2
Results of individual studies	#20	For all outcomes considered (benefits and harms), present, for each study: (a) simple summary data for each intervention group and (b) effect estimates and confidence intervals, ideally with a forest plot.	N/A
Synthesis of results	#21	Present the main results of the review. If meta-analyses are done, include for each, confidence intervals and measures of consistency.	10,11,12,13,14,15
Risk of bias across studies	#22	Present results of any assessment of risk of bias across studies (see Item 15).	10
Additional analysis	#23	Give results of additional analyses, if done (e.g., sensitivity or subgroup analyses, meta-regression [see Item 16]).	N/A

Discussion

For peer review only

Summary of Evidence	#24	Summarize the main findings, including the strength of evidence for each main outcome; consider their relevance to key groups (e.g., health care providers, users, and policy makers	15,16
Limitations	#25	Discuss limitations at study and outcome level (e.g., risk of bias), and at review level (e.g., incomplete retrieval of identified research, reporting bias).	16
Conclusions	#26	Provide a general interpretation of the results in the context of other evidence, and implications for future research.	17
**Funding**			
Funding	#27	Describe sources of funding or other support (e.g., supply of data) for the systematic review; role of funders for the systematic review.	1

Notes:

- 8: 3, appendix 1
- 9: 4, Figure 1
- 18: 5,6,7,8,9,10 (Table 1)
- 21: 10,11,12,13,14,15

The PRISMA checklist is distributed under the terms of the Creative Commons Attribution License CC-BY. This checklist was completed on 30. July 2019 using

For peer review only

https://www.goodreports.org/, a tool made by the EQUATOR Network in collaboration with Penelope.ai

For peer review only

BMJ Open

Priorities of clinicians and of multi-morbid patients regarding treatment and health outcome priorities: a systematic mixed studies review

Journal:	*BMJ Open*
Manuscript ID	bmjopen-2019-033445.R1
Article Type:	Original research
Date Submitted by the Author:	14-Nov-2019
Complete List of Authors:	Sathanapally, Harini; University of Leicester Diabetes Research Centre,<br>Sidhu, Manbinder S.; University of Birmingham,<br>Fahami, Radia; University of Leicester Diabetes Research Centre<br>Gillies, Clare; University of Leicester Diabetes Research Centre<br>Kadam , Umesh ; University of Leicester,<br>Davies, Melanie; University of Leicester Diabetes Research Centre<br>Khunti, Kamlesh; University of Leicester Diabetes Research Centre<br>Seidu, Samuel; University of Leicester Diabetes Research Centre
**Primary Subject Heading**:	General practice / Family practice
Secondary Subject Heading:	Patient-centred medicine, Communication
Keywords:	PRIMARY CARE, GENERAL MEDICINE (see Internal Medicine), QUALITATIVE RESEARCH

SCHOLARONE™
Manuscripts

I, the Submitting Author has the right to grant and does grant on behalf of all authors of the Work (as defined in the below author licence), an exclusive licence and/or a non-exclusive licence for contributions from authors who are: i) UK Crown employees; ii) where BMJ has agreed a CC-BY licence shall apply, and/or iii) in accordance with the terms applicable for US Federal Government officers or employees acting as part of their official duties; on a worldwide, perpetual, irrevocable, royalty-free basis to BMJ Publishing Group Ltd ("BMJ") its licensees and where the relevant Journal is co-owned by BMJ to the co-owners of the Journal, to publish the Work in this journal and any other BMJ products and to exploit all rights, as set out in our licence.

The Submitting Author accepts and understands that any supply made under these terms is made by BMJ to the Submitting Author unless you are acting as an employee on behalf of your employer or a postgraduate student of an affiliated institution which is paying any applicable article publishing charge ("APC") for Open Access articles. Where the Submitting Author wishes to make the Work available on an Open Access basis (and intends to pay the relevant APC), the terms of reuse of such Open Access shall be governed by a Creative Commons licence – details of these licences and which Creative Commons licence will apply to this Work are set out in our licence referred to above.

Other than as permitted in any relevant BMJ Author's Self Archiving Policies, I confirm this Work has not been accepted for publication elsewhere, is not being considered for publication elsewhere and does not duplicate material already published. I confirm all authors consent to publication of this Work and authorise the granting of this licence.

For peer review only

Priorities of clinicians and of multi-morbid patients regarding treatment and health outcome priorities: a systematic mixed studies review

Harini Sathanapally[1], Manbinder Sidhu[2], Radia Fahami[1], Clare Gillies[1], Umesh Kadam[1], Melanie J Davies[1], Kamlesh Khunti[1] and Samuel Seidu[1]

1. *Diabetes research centre, College of Life Sciences, University of Leicester, UK*
2. *Health Services Management Centre, School of Social Policy, University of Birmingham, UK*

Corresponding author: Dr Harini Sathanapally (hs333@student.le.ac.uk)

Word count excluding titles, abstract, article summary, tables, figures and references: 4992

Author contributions: HS: Design of research question and methodology, data searching, data extraction, data analysis and manuscript development; MS: Design of methodology, data extraction, data analysis and manuscript development; RF: Data searching and data extraction; CG: Data analysis and manuscript development; UK: Data analysis and manuscript development; MJD: Data analysis and manuscript development; KK: Design of research question and methodology, manuscript development; SS: Conception of the idea for this review, design of research question and methodology, data extraction, data analysis and manuscript development. All authors have approved the final manuscript.

Funding: Harini Sathanapally is funded by the NIHR academic clinical fellowship award

Competing interests: None declared

Patient consent for publication: Not required

Data sharing statement: The majority of the data extracted and analysed as part of this review has been included in this article and associated appendices. Any further data can be acquired from the corresponding author by request.

For peer review only

ABSTRACT

Objectives: To identify studies that have investigated the health outcome and treatment priorities of multi-morbid patients, clinicians, or both, in order to assess whether the priorities of the two groups are in alignment, or whether a disparity exists between the priorities of multi-morbid patients and clinicians.

Design: Systematic review

Data sources: MEDLINE, EMBASE, CINHAL and Cochrane databases from inception to May 2019 using a pre-defined search strategy, as well as reference lists containing any relevant articles, as per PRISMA and Cochrane guidelines.

Eligibility criteria: We included studies reporting health outcome and treatment priorities of adult multi-morbid patients, defined as suffering from two or more chronic conditions, or of clinicians in the context of multi-morbidity, or both. There was no restriction by study design, and studies using quantitative and/or qualitative methodologies were included.

Data synthesis: We used a narrative synthesis approach to synthesise the quantitative findings, and a meta-ethnography approach to synthesise the qualitative findings.

Results: Our search identified of 24 studies for inclusion, which comprised of 12 quantitative studies, 10 qualitative studies and two mixed-methods studies. Twelve studies reported the priorities of both patients and clinicians (seven quantitative, three qualitative and two mixed-methods studies), ten studies reported the priorities of patients alone (three quantitative and seven qualitative studies) and two studies reported the priorities of clinicians alone (two quantitative studies).

Conclusion: Our findings have shown that there is a mostly low level of agreement between the priorities of multi-morbid patients and clinicians. We found that prioritisation by multi-morbid patients was mainly driven by their illness experiences, whilst clinicians focused on longer term risks. Recognising that there may be a disparity in prioritisation and understanding the reasons for why this might occur, can facilitate clinicians in accurately eliciting the priorities that are most important to their patients and delivering patient-centred care.

KEY WORDS: Patient-centred care, Shared decision-making, Multi-morbidity

ARTICLE SUMMARY

Strengths and limitations

- This is the first systematic review to assimilate and compare the findings of existing literature on the health outcome and treatment priorities of both clinicians treating and patients living with multi-morbidity.
- We have included papers using both qualitative and quantitative methodologies and have been able to explore patterns and relationships in the findings, thus creating a comprehensive and well-rounded systematic review.
- Our findings facilitate clinicians in understanding both *how* and *why* the health outcome and treatment priorities of their multi-morbid patients might differ from their own priorities.
- Meta-analysis of the quantitative studies was unfeasible as there was a large variation in the tools used to ascertain priorities, and we have attempted to mitigate this by using a well-described and transparent method of narrative synthesis.
- A number of our included quantitative studies did not use pre-validated tools to ascertain priorities, leading to a risk of measurement bias.

For peer review only

INTRODUCTION

Multi-morbidity, defined as the co-existence of two or more long-term conditions [1] is a global problem [2], which has become the norm across high-income countries [2, 3][4, 5] and becoming increasingly prevalent in middle and low-income countries [6][7][2]. Guidelines for the management of chronic diseases are often single disease-orientated, and can lead to confusion and complications when applied to patients with multi-morbidity [8]. Multi-morbid patients have an increased risk of adverse drug-related events as a result of high levels of polypharmacy and receiving un-coordinated care from multiple healthcare providers [9]. These patients have a poorer health-related quality of life [10], poorer functional status [11] and greater psychological distress [12]. As a result, understanding and finding better strategies to facilitate the management of multi-morbid patients has been identified as a priority for health research [13] .

Key to the effective management of multi-morbidity is using patient-centred care and shared decision-making to set management goals that are acceptable to both the patient and the clinician [14]. Incorporating the priorities of patients in relation to treatments and health outcomes is integral to this process [15-17]. However, previous research has shown that whilst doctors recognise the importance of eliciting and incorporating the priorities of their multi-morbid patients, they do not always engage with this process in real world settings, and find eliciting patients' priorities to be difficult [18] [19]. Previous research, completed in a single disease context, has shown that the treatment and health outcome priorities of patients and clinicians can differ [20-22], and some studies have highlighted a gap between what doctors' perceive to be the priorities of their patients, and the actual priorities of their patients [23-25].

This systematic review aims to identify studies that have investigated the health outcome and treatment priorities of multi-morbid patients, clinicians, or both, in order to assess whether the priorities of the two groups are in alignment, or whether there is a disparity between the priorities of multi-morbid patients and clinicians.

METHODS

Search strategy

This systematic review has been registered on PROSPERO (ID: CRD42018076076). A comprehensive search strategy (Appendix 1), was developed using guidance for best practice [26] and input from academic librarians at the University of Leicester. The search strategy was used to search MEDLINE, EMBASE, CINHAL and COCHRANE databases from inception to May 2019, as well as searching reference lists for any relevant articles based on PRISMA and Cochrane guidelines [26-28]. We undertook a scoping search using google scholar using our key terms (Patient*; Priorit*; Clinician, Physician, Doctor, General-practitioner, Family-practitioner; Multi-morbid*) to identify relevant grey literature. Citations were stored using Refworks. We have presented our process of article selection in Figure 1.

We included studies reporting the health outcome and treatment priorities of adult patients with multimorbidity [1] and/or clinicians, in relation to patients with multi-morbidity. Studies which did not specify the definition of multi-morbidity as "two or more chronic conditions" [1] in their inclusion criteria, but had a sample patients representative of being diagnosed with multi-morbidity (i.e. with a minimum of two chronic conditions), were also included. There was no restriction by

For peer review only - http://bmjopen.bmj.com/site/about/guidelines.xhtml

study design, and we included studies using quantitative and/or qualitative methodologies. We excluded studies not published in English language, studies with participants aged under 18 years, and studies focusing on a single disease area.

Patient and Public Involvement

Patient and public involvement was not applicable in the design, conduct or reporting of this review.

Study selection

The titles and abstracts of all articles identified by the literature search were assessed independently and in duplicate by two reviewers (HS and RF). Studies that did not meet inclusion criteria were discarded. Full text of selected articles were retrieved and assessed to determine if they met the inclusion criteria, and those studies which met the inclusion criteria were included in the review. Any discrepancies regarding eligibility of an article were discussed, and consensus reached with MS and SS.

Methodological quality assessment and data extraction

Data was extracted using standardised data extraction forms by a single reviewer (HS), and these were checked independently for accuracy by a second reviewer (SS). The reported health outcome and treatment priorities of study participants were the key outcomes that were extracted.

Quality assessment was carried out in parallel with the data extraction process. For the quantitative studies, due to the heterogeneity of study design, we used the AXIS tool for assessment for the cross-sectional studies [29], the Newcastle-Ottawa scale for assessment of the longitudinal observational and cohort studies [30], and the Cochrane collaboration's risk of bias tool for assessment of randomised controlled trials [31]. For the qualitative studies, we used the CASP checklist for appraisal of qualitative research [32]. For the two mixed-methods studies, we used the AXIS tool [29] to assess the quantitative aspects of the study (both cross-sectional in study design), and the CASP checklist for qualitative research [32], to assess the qualitative aspects of these studies.

Data synthesis

We decided *a priori* not to carry out a meta-analysis due to the heterogeneity of the quantitative studies. Therefore, we have taken a narrative synthesis approach, described by Popay et al [33] to synthesise our quantitative findings. Our approach consists of three key steps:

1) *Development of a preliminary synthesis* in which study characteristics and descriptions are collated and findings presented in a summary table

2) Exploring relationships in the data between study characteristics and their findings, as well as between the reported findings across different studies with explanations considered where relationships were identified.

c) *Assessing the robustness of the synthesis using* quality assessment tools to guide conclusions and identify directions for clinical practice.

Qualitative studies were synthesised using a meta-ethnography approach [34, 35], which consisted of careful reading of the papers, extracting information regarding the context of the study and findings. *Key concepts* arising from each paper were also identified, with preservation of the terminology used by the authors where possible to ensure accurate representation of the findings of

For peer review only - http://bmjopen.bmj.com/site/about/guidelines.xhtml

the original studies. The key concepts across the papers were then *translated* using a table summarising the studies, their findings in relation to the key concepts and the *second order* interpretations of the authors, which enabled the exploration of any relationships and differences. The translations were then synthesised using a table containing the *first order* and *second order* interpretations for the key concepts across the studies, which then led to the development of further, *third order* interpretations by reviewers [34, 35].

From the results of our narrative synthesis of the quantitative studies and meta-ethnography of the qualitative studies, we considered how the findings of the two syntheses complement one another, particularly where our qualitative findings may provide possible explanations for our quantitative findings. The outcome of this process is described in the discussion section.

RESULTS

Overall study characteristics

Our search resulted in the identification of 24 studies for inclusion, which comprised of 12 quantitative studies, 10 qualitative studies and two mixed-methods studies. The characteristics of all of the included studies are described in Table 1. The included studies had all been conducted in high income developed countries, including Canada [36, 37], USA[38-44], Netherlands[45, 46], Australia[47, 48], UK[49-51], Germany [52-55]and Switzerland [56-58]. Sample sizes ranged from 15 to 1169 patients and 5 to 92 clinicians in the quantitative studies, and 15 to 146 patients and 4 to 19 clinicians in the qualitative studies.

Author and year of publication	Setting	Study type	Study aims	Target group and number of participants (n)	Outcomes measured
**QUANTITATIVE**					
**Health outcome priorities**					
Fried et al, 2011 [39]	USA- 3 senior centres and 1 assisted living facility	Quantitative: Cross-sectional study	*To explore the use of a simple tool to elicit older persons' health outcome priorities*	All volunteers included (n=357)	The prioritisation by participants of 4 universal health outcomes, namely:<br>-keeping alive<br>- maintaining independence<br>- reducing or eliminating pain<br>-reducing or eliminating other symptoms
Fried et al , 2011, [40]	USA- recruited from participants in a larger study, where they had been recruited from age-aggregated community housing [59]	Quantitative: Cross-sectional survey	*To determine the feasibility of using a simple tool to elicit the preferences of older persons based on their prioritization of universal outcomes*	Patients aged 65 and over with a known diagnosis of hypertension or use of anti-hypertensive medications, and having a known risk of falls (n=81)	> Rankings given by participants to 4 universal health outcomes in the outcome prioritisation tool:<br>--keeping alive<br>- maintaining independence<br>- reducing or eliminating pain<br>-reducing or eliminating other symptoms<br>> Feasibility of the use of outcome prioritisation tool

For peer review only - http://bmjopen.bmj.com/site/about/guidelines.xhtml

Mantelli et al, 2018[57]	Switzerland- General practitioners working in Switzerland who had previously taken part in case-vignette studies	Quantitative: cross-sectional survey	*To determine whether, how and why GPs de-prescribe in frail oldest-old patients with multimorbidity and polypharmacy, and to identify factors that influenced their decision to de-prescribe*	General Practitioners (n=157)	- Percentage of GPs willing to de-prescribe at least one medication in the case of frail older patients with CVD and compared to frail older patients without CVD<br>- Reasons for de-prescribing<br>- Importance ratings given to factors influencing decision to de-prescribe
Van Summeren et al, 2017 [46]	Netherlands- General practice centres	Quantitative: Cross-sectional and implementation study	*To determine proposed and observed medication changes when using an outcome prioritisation tool during a medication review in older patients with multimorbidity and polypharmacy. A secondary aim was to explore the relationship between the prioritized health outcome of patients and the type of medication change, such as a stop, a dose adjustment, or a switch.*	Patients aged 69 or over with two or more chronic diseases (one of which had to be cardiovascular disease) and daily use of five or more medications. (n=59)<br><br>General practitioners (n=17)	>Patients' priority rankings of the four health outcomes in the outcome prioritisation tool:<br>-Maintaining independence<br>-Remaining alive<br>-Reducing other symptoms<br>-Reducing pain<br><br>>Medication changes proposed by the GP, and observed in the patient records following incorporation of the priority rankings given by patients, into a medication review consultation.
Van Summeren et al, 2016 [45]	Netherlands- General practice centres	Mixed-methods: Cross-sectional survey pilot and qualitative interviews to assess acceptability (semi-structured and in-depth)	*To explore whether an outcome prioritization tool (OPT) is appropriate in the context of medication review in family practice, focusing on its acceptability and practicality*	Patients aged 69 or over with two or more chronic diseases (one of which had to be cardiovascular disease) and daily use of five or more medications (n=60)<br><br>General practitioners (n=13)	>Patients' prioritisation of the four domains of the outcome prioritisation tool:<br>-Maintaining independence<br>-Remaining alive<br>-Reducing other symptoms<br>-Reducing pain<br><br>> Family practitioners views on the acceptability and practicality of using the outcome prioritisation tool for medication review
**Problem-based priorities**					
Junius-Walker et al 2012 [52]	Germany- General practice centres	Quantitative: Randomised controlled trial	*To investigate whether a structured priority-setting consultation reconciles the often-differing doctor–patient views on the importance of problems.*	Patients aged 70 or over (n=317)<br><br>General practitioners (n=40)	-Baseline importance rankings given by patients and clinicians to a list of problems generated from a geriatric assessment for each patient.<br>- Importance rankings given again after a structured consultation incorporating the baseline problem list and importance rankings and degree of reconciliation in doctor-patient agreement after the structured consultation

For peer review only - http://bmjopen.bmj.com/site/about/guidelines.xhtml

Junius-Walker et al, 2011[53]	Germany-General practice centres	Quantitative: Cross-sectional survey	*To gain insight into setting individual priorities with older patients using a priority definition that was coherent to the patients' life and doctors' work context*	Patients aged 70 or over and living at home (n=123)<br><br>General practitioners (n=11)	Importance rankings given by patients and clinicians to a list of problems generated from a geriatric assessment for each patient.
Voigt et al, 2010 [54]	Germany-General practice centres	Quantitative: Cross-sectional survey	*To ascertain health priorities of older patients and treatment priorities of their general practitioners (GP) on the basis of a geriatric assessment and to determine the agreement between these priorities.*	Patients aged 70 or over and at least one contact with the general practitioner in the preceding 3 months (n= 35)<br><br>General practitioners (n=9)	-Importance rankings given to problems generated from a geriatric assessment by patients and clinicians<br>-Degree of agreement between patients and clinicians on the above
**Condition-focused priorities**					
Moore et al, 2014 [36]	Canada-Databases of all practising nurse practitioners, family practitioners and geriatricians in Ontario	Quantitative: Cross-sectional survey	*To quantify how family physicians, nurse practitioners and geriatricians prioritize syndromes, diseases and conditions when caring for seniors*	Nurse practitioners (n=68)<br><br>Family practitioners (n=84)<br><br>Geriatricians (n=27)	Frequency and importance rankings given by family practitioners, nurse practitioners and geriatricians to 41 health issues known to arise in elderly patients
Zulman et al, 2010 [44]	USA- Scheduled primary care visit for patients at 9 veteran affairs facilities	Quantitative: Prospective cohort study	*To understand patterns of patient-provider concordance in the prioritization of health conditions in patients with multimorbidity*	Patients with diabetes and hypertension who had their primary diabetes care provider enrolled in the study (n = 1169)<br><br>Primary care providers i.e. physicians, physician assistants or nurse practitioners (n= 92)	-Patient rankings given in terms of their most important health concerns and providers rankings in terms of conditions most likely to affect each patient's outcomes<br>-Concordance between the importance ratings of patient-provider "pairs"
Herzig et al, 2019 [56]	Switzerland-Primary data was from "Multimorbidity in Family medicine" study [60].<br><br>Patients enrolled by General practitioners during scheduled consultations.	Quantitative: Cross-sectional survey	*To describe FPs' medical priority ranking of conditions relative to their prevalence in patients with multimorbidity*	Patients suffering from at least 3 of 75 chronic conditions on a pre-defined list (based on the International classification of primary care 2 (n=888)<br><br>General Practitioners (n=100)	Importance rankings given by family practitioners to the list of chronic conditions that each patient had on the day of their inclusion in the study
Déruaz-Luyet et al, 2018 [58]	Switzerland-Primary data was from "Multimorbidity in Family medicine" study [60].	Quantitative: Cross-sectional survey	*To evaluate whether GPs could identify the condition that their patients with multimorbidity considered most important.*	Patients suffering from at least 3 of 75 chronic conditions on a pre-defined list (based on the International classification of primary care 2, and receiving follow-up from their GP for at least the	Whether there is agreement between what patients considered to be their most important health condition and what GPs thought patients considered to be their most important health condition

For peer review only - http://bmjopen.bmj.com/site/about/guidelines.xhtml

	Patients enrolled by General practitioners during scheduled consultations.			preceding 6 months (n= 572 for main analysis, 585 for sensitivity analysis)<br><br>General Practitioners (n=100)	
**Treatment priorities**					
Caughey et al, 2017 [47]	Australia- Multi-disciplinary ambulatory consulting service clinics at tertiary teaching hospitals	Mixed-methods: Structured quantitative interviews with patients then semi-structured qualitative interviews with patients and clinicians	*To investigate how older patients with multi-morbidity balance the benefits and harms associated with medication for prevention of CVD, and in the presence of competing health outcomes.*<br>*To investigate the factors that clinicians consider when making treatment decisions for older, multimorbid patients.*	Patients aged 65 or older with 2 or more chronic conditions (n=15)<br><br>Clinicians (n=5)	-Patient willingness to take a medication when presented with different scenarios with variable degree of benefit, impact on daily living, adverse outcomes and impact on other comorbid conditions<br><br>-Patient-reported data during semi-structured interviews where they were asked about their treatment preferences, medication effects and shared decision making<br><br>-Clinician reported data during semi-structured interviews on treatment decisions, patient preferences and polypharmacy.
**QUALITATIVE**					
Kuluski et a, 2013 [37]	Canada- A Family Health Team in Ontario	Qualitative: Semi-structured interviews	*To examine patient goals of care from the perspectives of older persons with multi-morbidities, their family physicians and informal caregivers (i.e., family member or friend who provides ongoing support) and then examine the extent of alignment between these three perspectives*	Patients aged 65 or older with a diagnosis of at least two chronic health conditions (n=28)<br><br>Informal Caregivers of included patients (n=28)<br><br>Family physicians (n=4)	>Patient, caregiver and physician reported data on goals of care for the patients<br><br>>Degree of alignment of goals of care across patient, caregiver and physician "triads"
Schoenberg et al, 2009 [38]	USA- Senior centres, Low income senior housing complexes, churches and a civic meeting hall	Qualitative: In-depth interviews	*To understand how vulnerable older adults with multimorbidity prioritize and manage their chronic conditions*	Patients aged 55 or older with a diagnosis of at least two chronic illnesses, from low-income backgrounds (n= 41)	Patient-reported data from in-depth interviews, regarding their medical history, self-care procedures, patient prioritisation by means of health-related areas of worry and health-related "expenditures" in terms of money, time and need for reliance on others.
Fried et al, 2008 [41]	USA- Senior centres, Doctors' practices and a congregate housing site	Qualitative: Focus groups	*To examine the ways in which older persons with multiple conditions think about potentially competing outcomes, in order*	Patients aged 65 or older and were taking 5 or more medications (participants also had a minimum of 3 chronic conditions)	Patient-reported data regarding their perceptions of the interactions between their different illnesses and treatment regimens, goals of treatment and decisions regarding treatment

For peer review only - http://bmjopen.bmj.com/site/about/guidelines.xhtml

			to gain insight into how processes to elicit values regarding these outcomes can be grounded in the patient's perspective		
Naik et al, 2016 [42]	USA- Qualitative data from the VETCARES study [61] , in which participants recruited from the VA tumour registry	Qualitative: Open-ended questions as part of mixed methods interviews which also included structured questions	*To identify a taxonomy of health-related values that frame goals of care of older, multi-morbid adults who recently faced cancer diagnosis and treatment*	Veterans with a diagnosis of head and neck, gastric, oesophageal, or colorectal cancer, and diagnosis fell one month prior to the study's opening eligibility window (6 months) (n=146)	Patient-reported data regarding their priorities or concerns regarding their future healthcare decisions
Elliott et al, 2007 [43]	USA- Harvard Pilgrim Health Centre, a HMO (health maintenance organisation) in New England	Qualitative: Semi-structured interviews	*To explore how older adults with multiple illnesses make choices about medicines*	Patients taking more than three medicines with purposive sampling to reflect symptomatic comorbidities and asymptomatic comorbidities and mental health issues (participants had a minimum of 3 comorbidities) (n=20)	Patient-reported data regarding beliefs about medicines, medicine-taking behaviour, historical vs potential choices between different medicines, and factors influencing these choices
Turner et al, 2016 [48]	Australia- Long term care facilities in South Australia	Qualitative: Nominal group technique	*To use nominal group technique to generate then rank factors that general medical practitioners, nurses, pharmacists and residents or their representatives perceive are most important when deciding whether or not to de-prescribe medication*	Residents/representatives of residents (n=11)<br><br>General Practitioners (n=19)<br><br>Nurses (n=12)<br><br>Pharmacists (n=14)	-Generated factors important for de-prescribing according to residents/resident representatives, general practitioners, nurses and pharmacists<br><br>-Priority rankings given by groups containing representatives from all of the above, to the list of priorities generated previously.
Lindsay, 2009 [49]	UK- Participants recruited from CHD registries in Greater Manchester as part of a larger RCT[62]	Qualitative: Focus groups and two interviews	*To use the concepts of "chronic illness trajectory" and "biographical disruption" to examine how patients self-manage multiple chronic conditions and especially how they prioritize their conditions*	Participants from the parent study who had more than one chronic condition (i.e. at least two) (n=53)	Patient-reported data regarding how they prioritised their multiple conditions, what strategies they used to cope with their conditions and barriers in being able to manage their illnesses
Cheraghi-Sohi et al, 2013 [50]	UK- secondary analysis of qualitative data from four other studies [63-66]	Qualitative: In-depth interviews	*To explore how and why people with multimorbidity prioritise some long-term conditions over others and what the potential implications may be for self-management activity, and in turn, suggest how*	Participants from original studies who had two or more long term conditions, and had given data regarding prioritisation (n=41)	Patient-reported data pertaining to prioritisation of their long term conditions

For peer review only - http://bmjopen.bmj.com/site/about/guidelines.xhtml

			such information may help clinicians negotiate the management of multimorbidity patients		
Morris et al [51]	UK- General Practices in North-West England	Qualitative: Semi-structured interviews	*To examine what influences self-management priorities for individuals with multiple long-term conditions and how this changes over time*	Patients with more than one chronic condition and at least one of COPD, IBS or Diabetes (n=21)	Patient-reported data on management strategies and experiences with primary health care, and data from follow-up interviews on any changes in their illness management.
Hansen et al, 2015 [55]	Germany- Participants recruited from the "Multicare cohort study" [67]	Qualitative: Focus groups	*To identify reasons for disagreement regarding illnesses between patients and their GPs*	Patients who had 3 or more chronic conditions from a list of 29 conditions (n=21)<br><br>General Practitioners of the recruited patients (n=15)	Data from separate focus groups for patients and clinicians in which any communication problems and reasons for disagreement between patients and clinicians were explored

Table 1 Characteristics of all of the included studies in order of reference

Summary of quality assessment

The outcome of quality assessment based on each of the afore-mentioned tools is summarised in Appendix 2. The majority of the quantitative studies were cross-sectional in design [36, 39, 40, 45, 46, 53, 54, 56-58] [47], including the quantitative elements of the two mixed-methods studies. The other studies included one cohort study [44] and one randomised controlled trial [52]. The cross-sectional studies were of moderate quality, with a number of studies having small sample sizes [40, 45, 46, 54]. The sample sizes of clinicians in most of the cross-sectional studies were particularly small, ranging from of 9 to 157 clinicians [45, 46, 54, 57], which impacts upon the generalisability and application of their findings. We noted that a number of the studies did not use pre-validated questions and tools to ascertain priorities [36, 54, 56-58], leading to a degree of subjectivity in the way in which priorities were ascertained, and the risk of measurement bias which again impacts on the generalisability of their findings.

The majority of the qualitative studies, including the qualitative aspects of the two mixed-methods studies, used interviews for data collection (n=8). Two studies used focus groups [41, 55], one study used a combination of focus groups and interviews [49] and one study used the nominal group technique [48]. The qualitative studies were of good quality, with appropriate use of qualitative methodology and transparent descriptions of the data analysis processes. Three studies only gave a limited description of their analytic process [47-49], with two of these studies [47, 48] and one mixed-methods study [45], not presenting any quotes.

QUANTITATIVE SYNTHESIS

Within our quantitative synthesis, we found that the studies focused either on the overall state of the patients' health, the problems posed by different chronic disease groups, or the patients' treatment regimens. Some of the quantitative studies elicited patient and/or clinician priorities as part of an intervention [52] [46]. Therefore, in order to reduce the risk of bias from the interventions, we included only the pre-intervention results from these studies.

For peer review only

Health outcome priorities

Four studies reported patient priorities of overall health outcomes using a “health outcome prioritisation tool” [39, 40, 45], which is a visual analogue scale requiring the following health outcomes to be given a score out of 100: “Maintaining independence”; “Staying alive”; “Pain relief”; “Symptom relief”. Maintaining independence was the outcome that had the highest importance after a pooling of the *most important* rankings from the four studies, followed by “Staying alive” (Table 2). For clinicians’ priorities, one study reported that 98% of a sample of 157 general practitioners identified the “quality of life for the patient”, and 96% identified the “life expectancy of the patient”, as the most important factors in influencing their clinical decision-making to de-prescribe for elderly, multi-morbid patients [57].

Study	*Health outcome prioritisation as a tool for decision making among older persons with multiple chronic conditions*[39]	*Health outcome prioritisation to elicit preferences of older persons with multiple health conditions*[40]	*Outcome prioritisation tool for medication review in older patients with multimorbidity: A pilot study in general practice*[46]	*Eliciting Preferences of multi-morbid Elderly Adults in Family Practice Using an Outcome Prioritisation Tool*[45]	*Aggregate ranking as most important (%)*
**Maintaining independence**	270 (75.6%)	34 (42.0%)	7 (36.8%)	19 (35.8%)	330 (64.7%)
**Staying alive**	40 (11.2%)	22 (27.2%)	6 (31.6%)	18 (34.0%)	86 (16.9%)
**Pain relief**	26 (7.3%)	17 (21.0%)	1 (5.3 %)	6 (11.3%)	50 (9.8%)
**Symptom relief**	21 (5.9%)	8 (9.8%)	5 (26.3%)	10 (18.9%)	44 (8.6%)
**Total number of participants**	357	81	19[a]	53	510

Table 2-Summary of most important rankings for studies using the Outcome Prioritisation Tool
a= although there were 59 patients included in this study [46] *priorities were only reported for 19 patients*

Priorities based on health problems

Three studies reported patient and general practitioners’ priorities based on various health *problems*, following a geriatric assessment [52-54]. These problems were then categorised into domains, and the importance rankings for each of the domains were presented. Problems in the domains of “Social” “Mood” and “Function” recurrently featured in the top four of the most highly ranked priorities by patients across all three studies. In terms of the importance rankings by clinicians, problems in the domains of “Mood” and “Function” also featured in the top four importance rankings across all three studies, whilst “Social” problems were rated highly in one study [53] and problems in the domain of “Medication” were ranked highly in the other two studies [52, 54]. Interestingly, the authors in one study[53] found that patients feeling “Emotionally affected” was the strongest predictor for a problem being rated as important (OR 11.1 CI 6.73 to 18.33), whereas “Poor prognosis” was the strongest predictor for clinicians (OR 6.39 CI 4.61 TO 8.87)

For peer review only - http://bmjopen.bmj.com/site/about/guidelines.xhtml

Condition-focused priorities

Two studies reported patient priorities in relation to specific conditions or groups of conditions [44, 58], in the context of multi-morbidity. Zulman et al. reported that “Diabetes/glycaemic control” was most frequently ranked as “most important”, with “Hypertension” coming second [44]. However, the sample of patients included in this study were all diabetic, hypertensive patients. Deruaz-luyet et al. found that musculoskeletal conditions including back pain, were most frequently reported to be the most important conditions for their patients, however endocrine/metabolic conditions (including obesity) were second and cardiovascular conditions were third [58].

Three studies reported condition-focused priorities of clinicians in the context of multi-morbidity. Herzig et al. reported the priorities of general practitioners alone [56], and found that “multiple sclerosis”, “mental retardation”, and “bronchus lung neoplasm” were all highly prioritised by their participants. Zulman et al reported the priorities of “primary care providers” who consisted of physicians, physician assistants or nurse practitioners [44], and found that diabetes was the top priority for primary care providers, with hypertension coming second, in alignment with their previously described patient priorities [44]. Moore et al. examined the priorities of different types of clinicians, including family physicians, geriatricians and nurse practitioners [36], and as with Zulman et al., found that diabetes was the top priority for family physicians and also nurse practitioners, whereas dementia was the top priority for geriatricians [36]. In addition, heart failure, atrial fibrillation and hypertension formed three of the top five conditions considered to be most important by the family practitioners in the study [36].

Treatment priorities

As part of a study to examine the influence of the risks and benefits of medications on treatment preferences of patients, Caughey et al. also examined the priorities of patients in the face of “competing outcomes” [47]. They found that 80% of participants would not be willing to take medication to reduce “joint pain”, if the medication increased their risk of a myocardial infarction by 10%. However, this was deduced from a sample of only 15 patients [47].

Agreement between patients and clinicians

Five of the included studies investigated the level of agreement in priority rankings between patients and their clinicians [44, 52-54, 58]. Three studies reported a low level of agreement between patient and clinicians’ priority rankings [52-54]. Two of these studies used a Cohen’s Kappa calculation to estimate the degree of agreement between the importance ratings of patients and clinicians, and the values of which were 0.18 and 0.11 respectively, indicating “slight agreement” after allowing for chance [53][54]. One study used a weighted kappa calculation to measure the degree of agreement, which, at a pre-intervention point in this study, was low at 6% [52].

Two studies reported that there was a “high” level of agreement [44, 58]. Deruaz-Luyet et al. found that in the case of 54.9% (n=314) of their patients, the condition that their GP had considered to be either the first or second most important, was in the same disease-group as the condition that the patient considered to be most important [58].

Zulman et al. reported that 60% of “patient-provider pairs” had a “high concordance”, meaning that the same three conditions had been rated as top three priorities by both parties, or that two of the same conditions had been rated in the top three priorities by both parties [44]. In this case, given that the sample of patients were all diabetic and hypertensive could have led to a narrowing of the

For peer review only - http://bmjopen.bmj.com/site/about/guidelines.xhtml

range of chronic diseases across the sample, which in turn could have led to an increased likelihood of agreement. However, the participant characteristics reported by the authors state that the patients had a mean of eight health conditions (SD 3.00), suggesting that the patients did not have a narrow range of chronic diseases. Furthermore, the questions posed to patients and providers were phrased differently, in that providers were asked to choose the top three most important medical concerns “that are likely to affect health outcomes for this patient”, whereas patients were asked to choose their top three most important health concerns. The authors acknowledge this in their paper, and justify this difference as being due to their aim of exploring the concordance in priorities about the “most important problems facing the patient”, rather which problems “providers thought the patient would have prioritised”, which, they argue, is a different concept to their aim [44].

QUALITATIVE SYNTHESIS

Whilst our quantitative synthesis allowed us to investigate *which* health outcomes, diseases or treatments were important to multi-morbid patients and their clinicians, our qualitative analysis enabled us to explore *how* prioritisation occurs. Below, we describe the key findings from our qualitative analysis.

Mechanisms of prioritisation

In the qualitative studies that approached prioritisation from a disease-specific perspective, patients were able to identify an illness as their main priority [49, 50]. For many patients, prioritisation appeared to be driven by their experience of the illness, which formed part of its “meaning as consequence” [50] as phrased by Cheraghi-Sohi et al. The ‘consequences’ of an illness consisted of the *impact* that the illness was having on the patients’ everyday lives, which included functional limitation and the symptomatic burden of the illness, including its “unpredictability” (Table 3) [49]. For others, prioritisation appeared to be driven by their perception of the risk now and in the future with respect to functional deterioration and mortality.

In other studies, patients framed their priorities between *quality of life vs length of life* (Table 3) [42]. Patients in the study by Naik et al. who were multi-morbid adults with cancer, prioritised “quality of life” more highly than “length of life” [42]. This was also reflected in the findings of Fried et al., who found that when considering medication with competing outcomes in terms of extending life compared to quality of life, participants appeared to prioritise preserving quality of life [41].

Van Summeren et al. found that prioritisation was “difficult” when there was no “specific need” for a treatment decision to be made [45]. This concept of a difference in prioritisation based on hypothetical, or experiential levels, was also shared in the findings of Elliott et al [43] and Fried et al [41].

Where clinicians’ perspectives were explored alongside patients, clinicians reported that exploring patients’ priorities was “extremely important” when managing “competing interests” [47] and beneficial in providing patient-centred care [45]. Some clinicians in the mixed-methods study carried out by Van Summeren et al. reported that exploring their patients’ priorities allowed them to have a “deeper understanding” of the patient, helped with making patient-centred treatment decisions and advance care planning (Table 3) [45]. However, other clinicians in the same study found exploring patient priorities to be difficult due its “novelty” and the fact that it represented a change to their usual consultations [45].

For peer review only - http://bmjopen.bmj.com/site/about/guidelines.xhtml

	Concept	Examples from included studies
Mechanisms of prioritisation	Unpredictability of symptoms	"My final issue is diverticulitis. In many ways that is the thing that makes the most impact on my life because of the unreliability of it. You make plans to do something to go somewhere and at the last minute you don't dare leave the house because you don't leave the loo. In itself it's not an important medical issue. It's the social problem more than anything else." – Lindsay et al [49]
	Quality of life vs length of life	"If you don't feel good, you can't take care of yourself and you have to depend on somebody else, what's the good of living another 10 years?"- Fried et al [41]
	Facilitating clinicians' decision making	"In future, I'll be happier to be more decisive in keeping an eye on what we do and do not do as regards this patient." Van Summeren et al [45]

Table 3- Examples from included studies for key concepts relating to mechanisms of prioritisation

Factors influencing prioritisation:

Our analysis revealed that there were a number of factors that appeared to influence how both patients and clinicians arrived at their priorities, and which priorities they chose.

i. Functional ability

Preserving functional ability as a priority for patients was a dominant concept across the majority of the qualitative studies [37, 38, 49, 51] [42][47][41]. Preserving independence emerged as the most significant reason for prioritising functional ability for patients, and maintaining the ability to engage in activities of daily living, mobility, maintaining cognitive ability and wanting to avoid being a "burden" or lacking social support to help them cope with functional deterioration (Table 4) [38, 49, 50].

Conditions which caused limitation to patients' ability to self-manage their health conditions, led to a "tension" between the patients' expectations of themselves and what they were physically able to do [51]. Lifestyle management, particularly reduced ability to exercise and the adverse impact of this on weight, was cited as part of patients' ability to self-manage [49].

Maintaining patients' functional ability was reported as a priority by some clinicians [37] [47]. Clinicians considered the wider implications of the patients' functional deterioration, particularly cognitive deterioration, and spoke of wanting to reduce the risk of "burnout" for the patients' family members/caregivers [37].

ii. Mortality

Reducing the risk of mortality emerged as a recurrent priority for clinicians [47, 55]. Caughey et al found that clinicians prioritised mortality in younger (less than 65 years) multi-morbid patients rather than older multi-morbid patients, as they felt they could be more "aggressive" in their treatment [47]. Reducing the risk of mortality also emerged as a priority for patients across a number of studies [37, 38, 43, 50, 51] [42]. Some patients found the asymptomatic nature of hypertension to be concerning; hence, the consequences of hypertension could be unpredictable, compared to some other chronic illnesses where symptoms can give warning of onset and severity (Table 4) [38, 43].

For peer review only

For peer review only - http://bmjopen.bmj.com/site/about/guidelines.xhtml

iii. Symptom control

The symptomatic burden of a condition contributed to its “meaning as consequence” for patients [50]. Symptoms were cited as being a cause of functional limitation [38, 49], and in some cases their “unpredictability” could cause significant disruption to patients’ daily lives [49]. Symptom control was reported to be a priority by some clinicians [37][47]. However, clinicians in one study considered symptom control to be less important, particularly when there was no risk of mortality [55]. In these cases, clinicians seemed to be aware that patients may still be prioritising symptom control highly, even if the clinicians did not (Table 4).

iv. Treatment burden

Factors related to the treatment burden of an illness appeared to adversely impact prioritisation for patients, leading to *de-prioritisation* of certain medications and treatments [38, 41, 43, 48]. Elliot et al. reported that cost and distressing side effects, were factors which led patients to stop taking a medication [43]. Similarly, Fried et al. found that patients reported unpleasant side effects to be a “competing outcome”, which negatively influenced their decision regarding continuing a medication [41]. However, difficulty with achieving control over the management of an illness, as well as requirement for high levels of engagement with self-management, emerged as factors that contributed to the prioritisation of an illness by some patients (Table 4) [49].

	Concept	Examples from included studies
Factors influencing prioritisation	Functional ability	“I mean, because I have to be mobile, I am living on my own, no one is going to take care of me, I have got to look after myself..” Cheraghi-Sohi et al [50]
	Mortality	“Well I really do worry the most about the high blood pressure. ’Cause see you know you got arthritis and you can tell when it’s coming on. But you can’t hardly tell about high blood pressure. It can just hit you like that [snaps fingers] ….”” Lindsay et al [49]
	Symptom control	“I would not want to live with pain. I won't allow that to happen”- Naik et al [42]
	Disparity in prioritisation of symptom control	“.. I talk [to her] for a quarter of an hour about this and that every time after which she replies, “but my vertigo,” and I answer every time, well, unfortunately there is nothing I can do about it, we have already tried and done everything. But it is probably the first diagnosis she will mention: “What are you suffering from?”. “Vertigo”. For me, this would be somewhere all the way at the bottom.” – Hansen et al [55]
	Treatment burden	“It’s the knee that’s the most concerning because everything else is controlled by tablets. The knee is a problem because if I have one little slip I’m in plaster again for 6 weeks.” Lindsay et al [49]

Table 4- Examples from included studies for key concepts relating to factors influencing prioritisation

DISCUSSION

Prioritisation as a concept is broad, context-dependent and difficult to confine into a single definitive definition. As a result, determining what can be interpreted as a health outcome or treatment priority as part of our study selection in this review, was inherently difficult. We excluded some studies that investigated the preferences of multi-morbid patients or clinicians, in contexts that we judged to be different to the aim of this review. These included patient preferences for healthcare

For peer review only - http://bmjopen.bmj.com/site/about/guidelines.xhtml

delivery [68][69], levels of engagement with self-management practices [70][71] and clinicians' experiences of the management of multi-morbid patients [18][72][73]. Whilst these studies represent very important areas of research, they were not within the scope of our aim in this review i.e. identifying studies that report the health outcome and treatment priorities of multi-morbid patients or those of clinicians in relation to multi-morbid patients. A discussion from our synthesis of findings of the included studies in this review is presented below.

Health outcome and treatment priorities

From our findings, patients' prioritisation appeared to be driven by weighing up the empirical compared to the hypothetical impact of a disease, whereby the empirical impact of a disease, which included its impact on function, symptomatic and treatment burden, was the most dominant driver of prioritisation. This is consistent with the findings of previous literature showing patients with rheumatoid arthritis who had reported experiencing higher levels of pain, were more likely to report pain as a priority [74].

Amongst empirical factors, preserving functionality emerged as most highly prioritised by patients amongst the quantitative studies that took a health outcome approach[39, 40, 46], whilst "function" was a domain that was prioritised highly by both patients and clinicians in the studies where prioritisation of various health *problems* were investigated [52-54]. From our qualitative findings, functional ability formed a key part of the preservation of various aspects of the patients' independence and their quality of life, as well as their ability to self-manage. Existing evidence shows that the prevalence of multi-morbidity is highest in those aged over 65 years [75], and the population for the majority of the included studies were older multi-morbid adults. This could provide an explanation for why preserving functionality was highly prioritised.

Prioritisation was not a static process and was subject to change, based on factors such as illness exacerbations, life events, whether there was a need for a treatment decision to be made, and whether the priority related to retrospective or prospective healthcare [49, 51]. When considering the hypothetical impact of an illness, perceptions of future risk came into play, and in particular, the risk of mortality [43]. This was particularly evident in relation to cardiovascular disease, where patients appeared to perceive the risk of mortality to be high [38].

Risk of mortality was a dominant driver for prioritisation amongst clinicians. This was shown in our quantitative synthesis, where amongst studies assessing disease-specific priorities, conditions with a higher risk of mortality, such as cardiovascular disease and diabetes, recurrently emerged as being highly prioritised by clinicians [36, 44, 56] and differentiated by age [47]. This age-based consideration could explain why clinicians prioritised "quality of life for the patient" as higher, albeit marginally, than "life expectancy of the patient" in their clinical decision-making for de-prescribing for elderly, multi-morbid patients [57].

Smith et al previously developed a "Core Outcome Set" [76] in which a Delphi consensus panel formed of 26 international health experts, identified and prioritised a set of outcomes tailored for application to research studies targeting multi-morbid patients. Mortality, mental health outcomes and quality of life featured most highly in their list of prioritised outcomes, which also emerged in this review. However, we found that relatively few studies reported the prioritisation of mental health outcomes, with the exception of the studies that took a *problem-based* approach to prioritisation, where problems with regard to "Mood" were prioritised highly by both patients and clinicians [52-54].

For peer review only - http://bmjopen.bmj.com/site/about/guidelines.xhtml

Our findings show a varying degree of agreement between the priorities of multi-morbid patients and clinicians. Previous studies carried out in the context of diabetes[77], and psoriasis[78] have found a low level of agreement on health outcome and treatment priorities between patients and clinicians, which correlates with the findings of some studies included in this review [52-54], but not others [44]. The nature of the patients' illnesses emerged as a factor for concordance or discordance of priorities with their clinicians [37]. Patients and clinicians were in agreement in situations where patients were currently experiencing an exacerbation of a particular condition, or had a "stable" state of health. However, in patients who suffered from illnesses with more complex courses, discordance of priorities tended to occur between patients and clinicians [37].

In recent times, the traditional *paternalistic* model for the doctor-patient relationship has given way to an *egalitarian* model [79], where doctors and patients each play an equitable role in a shared-decision making process, which places the patient at its core and thus achieving greater *patient-centred* care [80][79]. A shared agreement between patients and doctors on treatment priorities have been highlighted to play an important part in achieving patient-centred care and creating a *therapeutic alliance*, the benefits of which can include improved treatment adherence [79, 80]. Indeed, Jowsey et al found that agreement between patients and clinicians in the formulation of care plans promoted adherence to these plans, whereas a lack of agreement led to disengagement with care plans by patients [81].

Strengths and limitations

To our knowledge, this is the first systematic review to assimilate and compare the findings of existing literature on the health outcome and treatment priorities of both patients and clinicians for patients living with multi-morbidities. In this review, we have been able to add a novel line of argument to the ongoing discussion on this subject. By incorporating papers using both qualitative and quantitative methodologies, we have been able to explore patterns and relationships in the findings of a wide range of studies, thus creating a comprehensive and well-rounded systematic review.

There are noteworthy limitations. We did not include the term "comorbidity", in our search terms, and whilst "comorbidity" is distinctive from multi-morbidity, there is also some conceptual overlap between the two terms. We felt that including "comorbidity" in our search strategy would identify studies focusing on a specific condition rather than multi-morbidity.

A number of the quantitative studies did not use pre-validated tools to ascertain priorities [36, 54, 56-58], leading to a risk of measurement bias, which could limit the generalisability of findings in this review. We also detected a large variation in the tools used to ascertain priorities, which meant that carrying out a meta-analysis to synthesise the findings of the quantitative studies was not possible. Yet, we have tried to mitigate the lack of meta-analysis by using a well-described and well-established method of narrative synthesis [33], in order to maintain rigour and transparency.

Another limitation is that in our inclusion criteria we chose to also include studies which did not explicitly specify a definition of multi-morbidity as "two or more chronic conditions" in their inclusion criteria but had a sample of participants that were reflective of multi-morbidity (i.e. with a minimum of two chronic conditions which could be identified from participant demographic data). We chose to do this as in the absence of a universally accepted and uniform definition of multi-morbidity, we sought to base our judgement on the inclusivity of each paper on its value in answering our review question. This, along with the previously discussed difficulty in defining prioritisation, may have introduced a degree of subjective interpretation in the process of study

For peer review only - http://bmjopen.bmj.com/site/about/guidelines.xhtml

selection, despite our attempt to mitigate this by incorporating independent review of the results of our literature searching by two reviewers in duplicate.

Recommendations for the future

We recommend that future guidelines developed for clinicians in the management of multi-morbidity highlight the need to elicit and consider both short term and long term priorities for their patients', as our review has shown that patients' priorities for their current illness experiences and future risks posed by illnesses, may differ. In accordance with current NICE guidance, we also reiterate the need to review these priorities continually, and particularly when exacerbations, changes to illness course or treatment regimens, or other wider socially-contextualised changes occur in their patients' lives.

There was a large variation in how priorities were ascertained, and in the tools used to ascertain priorities. The relative lack of standardised and validated tools for use to ascertain patient priorities in everyday clinical practice has also been described in previous literature [82]. We highlight a need for the development of a standardised and validated tool that is acceptable to both patients and clinicians, and can be used to ascertain patient-priorities in the multiple dimensions described in this review. Such a tool would a valuable aid to treatment decision-making, advance care planning and achieving patient-centeredness for patients living with multi-morbidity.

Conclusion

The findings from this review show the priorities of patients and clinicians can have varying degrees of concordance, being mostly low [52, 54], in alignment with previous findings in single disease contexts [77, 78]. We have found that the mechanisms of prioritisation can also differ between our two groups, in that patients are driven by illness experiences, whereas clinicians may be focused on managing longer term risks. Understanding these differences can help clinicians to better recognise situations where the patients' priorities may be different to theirs and elicit the most important priorities for their patients.

For peer review only - http://bmjopen.bmj.com/site/about/guidelines.xhtml

REFERENCES

1 van den Akker M, Buntinx F, Knottnerus JA. Comorbidity or multimorbidity: what's in a name? A review of literature, *The European Journal of General Practice* 1996;2:65-70.

2 Garin N, Koyanagi A, Chatterji S, et al. Global multimorbidity patterns: a cross-sectional, population-based, multi-country study, *Journals of Gerontology Series A: Biomedical Sciences and Medical Sciences* 2015;71:205-14.

3 Rijken M, Struckmann V, Dyakova M, et al. ICARE4EU: Improving care for people with multiple chronic conditions in Europe. 2013.

4 Roberts KC, Rao DP, Bennett TL, et al. Prevalence and patterns of chronic disease multimorbidity and associated determinants in Canada, *Health Promot Chronic Dis Prev Can* 2015;35:87-94.

5 Rocca WA, Boyd CM, Grossardt BR, et al. Prevalence of multimorbidity in a geographically defined American population: patterns by age, sex, and race/ethnicity. 2014;89:1336-49.

6 Arokiasamy P, Uttamacharya U, Jain K, et al. The impact of multimorbidity on adult physical and mental health in low-and middle-income countries: what does the study on global ageing and adult health (SAGE) reveal? *BMC medicine* 2015;13:178.

7 Afshar S, Roderick PJ, Kowal P, et al. Multimorbidity and the inequalities of global ageing: a cross-sectional study of 28 countries using the World Health Surveys, *BMC Public Health* 2015;15:776.

8 Tinetti ME, Bogardus Jr ST, Agostini JV. Potential pitfalls of disease-specific guidelines for patients with multiple conditions, *N Engl J Med* 2004;351:2870-4.

9 Calderon-Larranaga A, Poblador-Plou B, Gonzalez-Rubio F, et al. Multimorbidity, polypharmacy, referrals, and adverse drug events: are we doing things well? *Br J Gen Pract* 2012;62:e821-6.

10 Fortin M, Bravo G, Hudon C, et al. Relationship between multimorbidity and health-related quality of life of patients in primary care, *Quality of Life Research* 2006;15:83-91.

11 Marengoni A, Angleman S, Melis R, et al. Aging with multimorbidity: a systematic review of the literature, *Ageing research reviews* 2011;10:430-9.

12 Fortin M, Bravo G, Hudon C, et al. Psychological distress and multimorbidity in primary care, *Ann Fam Med* 2006;4:417-22.

13 Academy of Medical Sciences. Multimorbidity: a priority for global health research, 2018.

14 Muth C, van den Akker M, Blom JW, et al. The Ariadne principles: how to handle multimorbidity in primary care consultations. *BMC Medicine* 2014;12:223.

15 Azad NA, Mielniczuk L. A call for collaboration: improving cardiogeriatric care, *Can J Cardiol* 2016;32:1041-4.

16 Roland M, Paddison C. Better management of patients with multimorbidity, *BMJ: British Medical Journal (Online)* 2013;346.

For peer review only

17 Bierman AS, Tinetti ME. Precision medicine to precision care: managing multimorbidity, *Lancet* 2016;388:2721-3.

18 Mc Namara KP, Breken BD, Alzubaidi HT, et al. Health professional perspectives on the management of multimorbidity and polypharmacy for older patients in Australia, *Age & Ageing* 2017;46:291-9.

19 Sinnott C, Mc Hugh S, Browne J, et al. GPs' perspectives on the management of patients with multimorbidity: systematic review and synthesis of qualitative research, *BMJ Open* 2013;3:e003610,2013-003610.

20 Rothwell PM, McDowell Z, Wong CK, et al. Doctors and patients don't agree: cross sectional study of patients' and doctors' perceptions and assessments of disability in multiple sclerosis, *BMJ* 1997;314:1580-3.

21 Montgomery AA, Fahey T. How do patients' treatment preferences compare with those of clinicians? *Qual Health Care* 2001;10 Suppl 1:i39-43.

22 Thomson S, Doody G. Parallel paths? Patient and doctor priorities in psychiatric outpatient consultations, *Journal of Mental Health* 2010;19:461-9.

23 Lee CN, Hultman CS, Sepucha K. Do patients and providers agree about the most important facts and goals for breast reconstruction decisions? *Ann Plast Surg* 2010;64:563-6.

24 Volandes AE, Paasche-Orlow MK, Barry MJ, et al. Video decision support tool for advance care planning in dementia: randomised controlled trial, *BMJ* 2009;338:b2159.

25 Pager CK, McCluskey PJ. Surgeons' perceptions of their patients' priorities, *Journal of Cataract & Refractive Surgery* 2004;30:591-7.

26 Higgins JP, Green S. Cochrane handbook for systematic reviews of interventions: John Wiley & Sons 2011.

27 Moher D, Shamseer L, Clarke M, et al. Preferred reporting items for systematic review and meta-analysis protocols (PRISMA-P) 2015 statement, *Systematic reviews* 2015;4:1.

28 Moher D, Liberati A, Tetzlaff J, et al. Preferred reporting items for systematic reviews and meta-analyses: the PRISMA statement, *Ann Intern Med* 2009;151:264-9.

29 Downes MJ, Brennan ML, Williams HC, et al. Development of a critical appraisal tool to assess the quality of cross-sectional studies (AXIS), *BMJ Open* 2016;6:e011458,2016-011458.

30 Stang A. Critical evaluation of the Newcastle-Ottawa scale for the assessment of the quality of nonrandomized studies in meta-analyses, *Eur J Epidemiol* 2010;25:603-5.

31 Higgins JP, Altman DG, Gotzsche PC, et al. The Cochrane Collaboration's tool for assessing risk of bias in randomised trials, *BMJ* 2011;343:d5928.

32 Critical Appraisal Skills Programme UK. CASP qualitative research checklist. *CASP checklists* 13/03/2017.

For peer review only

For peer review only - http://bmjopen.bmj.com/site/about/guidelines.xhtml

33 Popay J, Roberts H, Sowden A, et al. Guidance on the conduct of narrative synthesis in systematic reviews, *A product from the ESRC methods programme Version* 2006;1:b92.

34 Noblit GW, Hare RD. Meta-ethnography: Synthesizing qualitative studies: sage 1988.

35 Britten N, Campbell R, Pope C, et al. Using meta ethnography to synthesise qualitative research: a worked example, *J Health Serv Res Policy* 2002;7:209-15.

36 Moore A., Patterson C., Nair K., et al. Minding the gap: Prioritization of care issues among nurse practitioners, family physicians and geriatricians when caring for the elderly. *Journal of interprofessional care* 2015;29:401-3.

37 Kuluski K, Gill A, Naganathan G, et al. A qualitative descriptive study on the alignment of care goals between older persons with multi-morbidities, their family physicians and informal caregivers. *BMC Family Practice* 2013;14:133.

38 Schoenberg NE, Leach C, Edwards W. "It's a toss up between my hearing, my heart, and my hip": prioritizing and accommodating multiple morbidities by vulnerable older adults, *J Health Care Poor Underserved* 2009;20:134-51.

39 Fried TR, Tinetti ME, Iannone L, et al. Health outcome prioritization as a tool for decision making among older persons with multiple chronic conditions, *Arch Intern Med* 2011;171:1856-8.

40 Fried TR, Tinetti M, Agostini J, et al. Health outcome prioritization to elicit preferences of older persons with multiple health conditions, *Patient Educ Couns* 2011;83:278-82.

41 Fried TR, McGraw S, Agostini JV, et al. Views of older persons with multiple morbidities on competing outcomes and clinical decision-making, *J Am Geriatr Soc* 2008;56:1839-44.

42 Naik A.D., Martin L.A., Moye J., et al. Health Values and Treatment Goals of Older, Multimorbid Adults Facing Life-Threatening Illness. *J Am Geriatr Soc* 2016;64:625-31.

43 Elliott RA, Ross-Degnan D, Adams AS, et al. Strategies for coping in a complex world: adherence behavior among older adults with chronic illness, *Journal of General Internal Medicine* 2007;22:805-10.

44 Zulman D.M., Kerr E.A., Hofer T.P., et al. Patient-provider concordance in the prioritization of health conditions among hypertensive diabetes patients. *Journal of General Internal Medicine* 2010;25:408-14.

45 Summeren JJ, Haaijer-Ruskamp FM, Schuling J. Eliciting preferences of multimorbid elderly adults in family practice using an outcome prioritization tool, *J Am Geriatr Soc* 2016;64.

46 van Summeren JJ, Schuling J, Haaijer-Ruskamp FM, et al. Outcome prioritisation tool for medication review in older patients with multimorbidity: a pilot study in general practice, *Br J Gen Pract* 2017;67:e501-6.

47 Caughey G.E., Tait K., Vitry A.I., et al. Influence of medication risks and benefits on treatment preferences in older patients with multimorbidity. *Patient Preference and Adherence* 2017;11:131-40.

For peer review only - http://bmjopen.bmj.com/site/about/guidelines.xhtml

48 Turner J.P., Edwards S., Stanners M., et al. What factors are important for deprescribing in Australian long-term care facilities? Perspectives of residents and health professionals. *BMJ Open* 2016;6:Arte Number: e009781. ate of Pubaton: 2016.

49 LINDSAY S. PRIORITIZING ILLNESS: LESSONS IN SELFa MANAGING MULTIPLE CHRONIC DISEASES, *CANADIAN JOURNAL OF SOCIOLOGY/CAHIERS CANADIENS DE SOCIOLOGIE* 2009;34:983.

50 Cheraghi-Sohi S, Morden A, Bower P, et al. Exploring patient priorities among long-term conditions in multimorbidity: A qualitative secondary analysis. *SAGE Open Medicine* 2013;1:2050312113503955.

51 Morris R.L., Sanders C., Kennedy A.P., et al. Shifting priorities in multimorbidity: A longitudinal qualitative study of patient's prioritization of multiple conditions. *Chronic Illness* 2011;7:147-61.

52 Junius-Walker U, Wrede J, Voigt I, et al. Impact of a priority-setting consultation on doctor-patient agreement after a geriatric assessment: cluster randomised controlled trial in German general practices. *Quality in primary care* 2012;20.

53 Junius-Walker U, Stolberg D, Steinke P, et al. Health and treatment priorities of older patients and their general practitioners: a cross-sectional study. *Quality in primary care* 2011;19.

54 Voigt I, Wrede J, Diederichs-Egidi H, et al. Priority setting in general practice: health priorities of older patients differ from treatment priorities of their physicians, *Croat Med J* 2010;51:483-92.

55 Hansen H., Pohontsch N., van den Bussche H., et al. Reasons for disagreement regarding illnesses between older patients with multimorbidity and their GPs - a qualitative study. *BMC family practice* 2015;16:68.

56 Herzig L, Mueller Y, Haller DM, et al. Family practitioners' top medical priorities when managing patients with multimorbidity: a cross-sectional study, *BJGP open* 2019;3:bjgpopen18X101622.

57 Mantelli S, Jungo KT, Rozsnyai Z, et al. How general practitioners would deprescribe in frail oldest-old with polypharmacy—the LESS study, *BMC family practice* 2018;19:169.

58 Déruaz-Luyet A, N'Goran AA, Pasquier J, et al. Multimorbidity: can general practitioners identify the health conditions most important to their patients? Results from a national cross-sectional study in Switzerland, *BMC family practice* 2018;19:66.

59 Tinetti ME, McAvay GJ, Fried TR, et al. Health outcome priorities among competing cardiovascular, fall injury, and medication-related symptom outcomes, *J Am Geriatr Soc* 2008;56:1409-16.

60 DeruazLuyet A., Alexandra N'Goran A., Tandjung R., et al. Multimorbidity in primary care: Protocol of a national cross-sectional study in Switzerland. *BMJ Open* 2015;5:Arte Number: e009165. ate of Pubaton: 2015.

61 Martin LA, Moye J, Street Jr RL, et al. Reconceptualizing cancer survivorship through veterans' lived experiences, *J Psychosoc Oncol* 2014;32:289-309.

62 Lindsay S, Bellaby P, Smith S, et al. Enabling healthy choices: is ICT the highway to health improvement? *Health:* 2008;12:313-31.

For peer review only - http://bmjopen.bmj.com/site/about/guidelines.xhtml

63 Hurley MV, Walsh N, Bhavnani V, et al. Health beliefs before and after participation on an exercised-based rehabilitation programme for chronic knee pain: doing is believing, *BMC Musculoskeletal Disorders* 2010;11:31.

64 Bower P, Harkness E, Macdonald W, et al. Illness representations in patients with multimorbid long-term conditions: Qualitative study, *Psychol Health* 2012;27:1211-26.

65 Grime J, Richardson JC, Ong BN. Perceptions of joint pain and feeling well in older people who reported being healthy: a qualitative study, *Br J Gen Pract* 2010;60:597-603.

66 Nio Ong B, Jinks C, Morden A. The hard work of self-management: Living with chronic knee pain, *International journal of qualitative studies on health and well-being* 2011;6:7035.

67 Hansen H., Schafer I., Schon G., et al. Agreement between self-reported and general practitioner-reported chronic conditions among multimorbid patients in primary care - results of the MultiCare Cohort Study. *BMC family practice* 2014;15:39.

68 Noel P.H., Frueh B.C., Larme A.C., et al. Collaborative care needs and preferences of primary care patients with multimorbidity. *Health Expectations* 2005;8:54-63.

69 Lechner S., Herzog W., Boehlen F., et al. Control preferences in treatment decisions among older adults - Results of a large population-based study. *J Psychosom Res* 2016;86:28-33.

70 Noel PH, Parchman ML, Williams JWJ, et al. The challenges of multimorbidity from the patient perspective. *Journal of General Internal Medicine* 2007;22:419-24.

71 Coventry PA, Fisher L, Kenning C, et al. Capacity, responsibility, and motivation: a critical qualitative evaluation of patient and practitioner views about barriers to self-management in people with multimorbidity, *BMC health services research* 2014;14:536.

72 Sinnott C., Mc Hugh S., Boyce M.B., et al. What to give the patient who has everything? A qualitative study of prescribing for multimorbidity in primary care. *British Journal of General Practice* 2015;65:e184-91.

73 Luijks HD, Loeffen MJW, Lagro-Janssen AL, et al. GPs' considerations in multimorbidity management: a qualitative study. *British Journal of General Practice* 2012;62:e503-10.

74 Heiberg T, Kvien TK. Preferences for improved health examined in 1,024 patients with rheumatoid arthritis: pain has highest priority, *Arthritis Care & Research* 2002;47:391-7.

75 Barnett K, Mercer SW, Norbury M, et al. Epidemiology of multimorbidity and implications for health care, research, and medical education: a cross-sectional study, *The Lancet* 2012;380:37-43.

76 Smith SM, Wallace E, Salisbury C, et al. A Core Outcome Set for Multimorbidity Research (COSmm), *Ann Fam Med* 2018;16:132-8.

77 Heisler M, Vijan S, Anderson RM, et al. When do patients and their physicians agree on diabetes treatment goals and strategies, and what difference does it make? *Journal of general internal medicine* 2003;18:893-902.

For peer review only

78 Okubo Y, Tsuruta D, Tang A, et al. Analysis of treatment goal alignment between Japanese psoriasis patients and their paired treating physicians, *Journal of the European Academy of Dermatology and Venereology* 2018;32:606-14.

79 Mead N, Bower P. Patient-centredness: a conceptual framework and review of the empirical literature, *Soc Sci Med* 2000;51:1087-110.

80 Kaba R, Sooriakumaran P. The evolution of the doctor-patient relationship, *International Journal of Surgery* 2007;5:57-65.

81 Jowsey T, Dennis S, Yen L, et al. Time to manage: patient strategies for coping with an absence of care coordination and continuity, *Sociol Health Illn* 2016;38:854-73.

82 Mangin D., Stephen G., Bismah V., et al. Making patient values visible in healthcare: A systematic review of tools to assess patient treatment priorities and preferences in the context of multimorbidity. *BMJ Open* 2016;6:Arte Number: e010903. ate of Pubaton: 01 Jun 2016.

Figure legends:

Figure 1: Flow diagram to illustrate process from literature searching to selection of studies for inclusion [28]

For peer review only

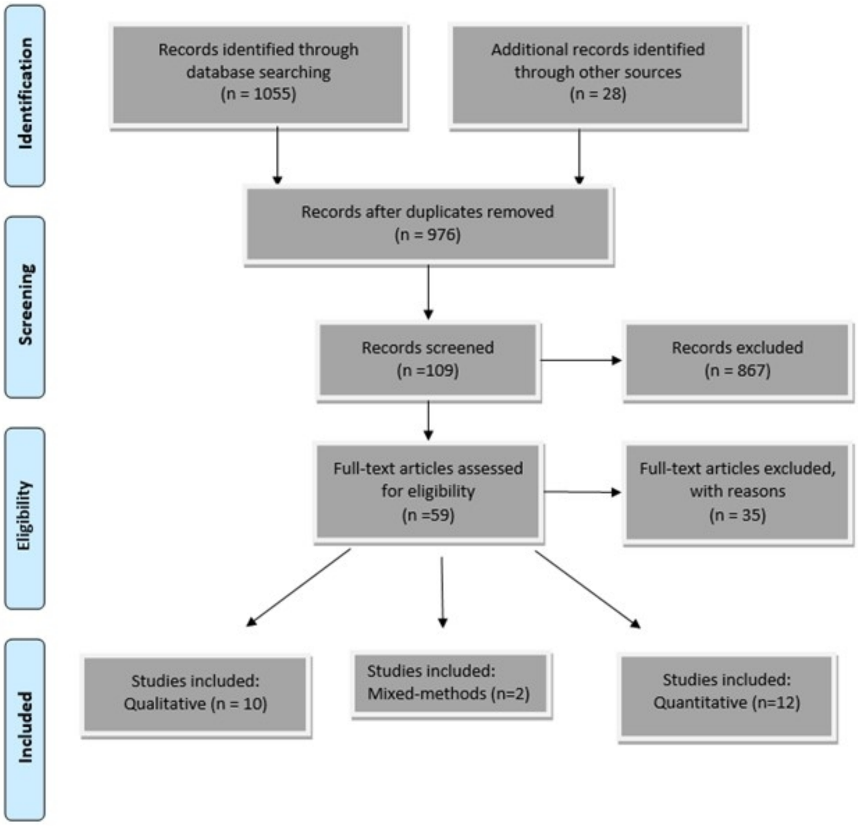

Figure 1 Flow diagram to illustrate process from literature searching to selection of studies for inclusion [28]

121x111mm (150 x 150 DPI)

**1. Patient*.mp.**
**2. Patients/**
**3. 1 or 2**
**4. Priorit*.mp. [mp=title, abstract, original title, name of substance word, subject heading word, keyword heading word, protocol supplementary concept word, rare disease supplementary concept word, unique identifier, synonyms]**
**5. Choice*.mp.**
**6. Preference*.mp.**
**7. Aim*.mp.**
**8. Goal*.mp.**
**9. 4 or 5 or 6 or 7 or 8**
**10. Doctor*.mp.**
**11. Physicians/**
**12. Clinician*.mp.**
**13. Primary Health Care/ or Physicians, Family/ or Family Practice/ or General Practitioners/**
**14. General practitioner*.mp.**
**15. 10 or 11 or 12 or 13 or 14**
**16. Multimorbidit*.mp. [mp=title, abstract, original title, name of substance word, subject heading word, keyword heading word, protocol supplementary concept word, rare disease supplementary concept word, unique identifier, synonyms]**
**17. Multi-morbidit*.mp. [mp=title, abstract, original title, name of substance word, subject heading word, keyword heading word, protocol supplementary concept word, rare disease supplementary concept word, unique identifier, synonyms]**
**18. Multiple morbidit*.mp. [mp=title, abstract, original title, name of substance word, subject heading word, keyword heading word, protocol supplementary concept word, rare disease supplementary concept word, unique identifier, synonyms]**
**19. 16 or 17 or 18**
**20. 3 and 9 and 15 and 19**
**21. Multi morbid*.mp. [mp=title, abstract, original title, name of substance word, subject heading word, keyword heading word, protocol supplementary concept word, rare disease supplementary concept word, unique identifier, synonyms]**
**22. 16 or 17 or 18 or 21**
**23. 3 and 9 and 15 and 22**

Appendix 1

For peer review only

CASP checklist for qualitative research summary table

	**Kuluski et al** [1]	**Schoenberg et al** [2]	**Cheraghi-Sohi et al** [3]	**Naik et al** [4]	**Lindsay et al** [5]	**Hansen et al** [6]	**Morris et al** [7]	**Elliott et al** [8]	**Fried et al** [9]	**Turner et al** [10]	**Van Summeren et al** [11]	**Caughey et al** [12]
Was there a clear statement of the aims of the research?	YES	YES	YES	YES	YES	YES	YES	YES	YES	YES	YES	YES
Is a qualitative methodology appropriate?	YES	YES	YES	YES	YES	YES	YES	YES	YES	NO- Quantitative or mixed methods methodology would have been more appropriate as the aim was to rank factors, although data collected using a qualitative technique, it lacks richness and appears to be presented in a quantitative manner	YES	YES
Was the research design appropriate to the aims of the research?	YES	YES	YES	YES	YES	YES	YES	YES	YES	YES	YES	YES
Was the recruitment strategy	YES	YES	YES	YES	YES	YES	NO- no explanation given as to	YES	YES	YES	YES	YES

For peer review only

appropriate to the aims of the research							why the specific conditions were chosen (COPD, IBS etc)					
Were the data collected in a way that addressed the research issue?	YES	YES	YES	YES	YES	YES	YES	YES	YES	YES	YES	YES
Has the relationship between researcher and participants been adequately considered?	YES	YES	YES	NO- no information given on background of main researcher and no consideration given to possibility of researcher bias at any point.	NO- There is no background information given on the researcher (sole in this case) and there has been no evidence of any consideration of researcher bias at any point during the study.	NO- there has been no evidence of any consideration of researcher bias at any point during the study	NO- no information given on background of main researcher and no consideration given to possibility of researcher bias at any point. However there was some evidence of reflexivity during the data collection process when emerging areas of interest that could be incorporated into future interviews	NO- background of RAE who conducted interviews and main aspect of analysis not specified and no consideration has been given to any possibility of researcher bias	NO- No explanatio n given of the profession al backgroun d of the researcher s or the moderator for the focus groups, and there has been no evidence of any considerati on of researcher bias at any point during the study.	NO- no mention of the background of the researchers or how this may have influenced the results	NO- role of second interviewer carrying out the in-depth interviews not mentioned, and there has been no consideration given to the possibility of bias from the interviewers. One of the interviewers was a FP, which could have led to bias with the interviewees responses.	NO- there has been no consideratio n given to the role of the researcher and the potential for researcher bias at any point.

							were considered.					
Have ethical issues been taken into consideration ?	YES	YES	YES- in the original studies, however further ethical issues regarding secondary qualitative analysis were not taken into account.	YES	YES	YES	YES	YES	YES	YES	YES	YES
Was the data analysis sufficiently rigorous?	YES	YES	YES	YES	NO- superficial description of analytic process and no information given on how many researchers analysed the transcripts- assumed one as there is only one author- risk of bias not taken into account for the analytic process	YES	YES	YES	YES	NO- the data analysis process is very ambiguous and the qualitative analysis has not been described in sufficient depth.	YES- clear description of the analytic process with two researchers independently analysing the data for rigour. However no description of the interpretation phase from the data.	NO- there is only a superficial description of the data analysis process, and there is very little detail given on how the themes were derived from the data. There is no presentation at all of quotes from the data to support the authors interpretation of the data.
Is there a clear	YES	YES	YES	YES	YES	YES	YES	YES	YES	YES	YES- however the qualitative	YES- however no quotes given

For peer review only

statement of findings?											data from the patient interviews has only been summarised- no direct quotes given	to support findings
How valuable is the research?	Valuable	Valuable	Valuable	Valuable	Valuable	Valuable	Valuable	Valuable	Valuable	Valuable	Valuable	Valuable

For peer review only

Newcastle-ottawa scale for cohort and observational studies summary table

	Representativeness of the exposed cohort	Selection of the non-exposed cohort	Ascertainment of exposure	Demonstration that outcome of interest was not present at start of study	Comparability of cohorts on the basis of the design or analysis controlled for confounders	Assessment of outcome	Was follow-up long enough for outcomes to occur	Adequacy of follow-up of cohorts
Zulman et al [13]	Somewhat representative (one star) *	Drawn from the same community as the exposed cohort (one star) *	Secure record (one star) *	N/A	The study controls for age, sex and marital status (one star)*	Self-report	N/A	No statement

For peer review only

Axis tool for cross-sectional studies summary table

**Introduction**		Junius-Walker et al [14]	Fried et al[15]	Fried et al [16]	Moore et al [17]	Van Summeren et al [18]	Voigt et al [19]	Van Summeren et al [11]	Caughey et al [20]	Mantelli et al [21]	Deruaz-Luyet et al [22]	Herzig et al [23]
1	Were the aims/objectives of the study clear?	Yes	Yes	Yes	Yes	Yes	Yes	Yes	Yes	Yes	Yes	Yes
Methods												
2	Was the study design appropriate for the stated aim(s)?	Yes	Yes	Yes	Yes	Yes	Yes	Yes	Yes	Yes	Yes	Yes
3	Was the sample size justified?	No- convenience sampling used, small sample size, however no explanation for sample size given	No- no justification for sample size given, convenience sampling used	No- recruitment strategy described clearly but no justification for sample size given	Yes	No	No- sampling strategy described well but no justification for sample size given	No- purposive sampling used, however no justification for sample size given	No- no justification for sample size given	No- convenience sampling used and no justification for sample size given	Yes- in the parent study [24]	Yes- in the parent study [24]
4	Was the target/reference population clearly defined? (Is it clear who the research was about?)	Yes	Yes	Yes	Yes	Yes	Yes	Yes	Yes	Yes	Yes	Yes
5	Was the sample frame taken from an appropriate population base so that it closely represented the target/reference population under investigation?	Yes	Yes- However assumption made that participants will have multiple chronic conditions	Yes	Yes	Yes	Yes	Yes	Yes	Yes- although only GP's who had previously taken part in other case-vignette studies were invited, leading to possibility of selection bias	Yes	Yes

For peer review only

6	Was the selection process likely to select subjects/participants that were representative of the target/reference population under investigation?	Yes	Yes- as above	Yes	Yes	Yes	Yes	Yes	Yes	Yes- as above	Yes	Yes
7	Were measures undertaken to address and categorise non-responders?	Yes	Don’t know- not reported	Yes	No	No	No	Yes- Purposive sampling used with efforts made to address gaps in participant types	Don’t know- not reported	Don’t know- not reported	Yes in the parent study [25].<br><br>Characteristics of participants who were not included due to missing data, were described in this study	Yes in the parent study [25]
8	Were the risk factor and outcome variables measured appropriate to the aims of the study?	Yes	Yes	Yes	Yes	Yes	Yes	Yes	Yes	Yes	Yes	Yes
9	Were the risk factor and outcome variables measured correctly using instruments/measurements that had been trialled, piloted or published previously?	Yes	Yes- piloted in a previous study	No- Tested in this study as it was a feasibility study	No- Pre-tested in this study but only using 2 FP’s and 1 NP	Yes	No- STEP assessment previously published however no testing done of measure used to collect importance ratings	Yes	Yes	Yes- the instruments used were piloted within this study using 5 GP’s as participants, but had not been published previously	No- instruments designed through “internal consensus discussions”.	No

For peer review only

10	Is it clear what was used to determined statistical significance and/or precision estimates? (e.g. p-values, confidence intervals)	Yes	N/A	Yes	Yes	N/A	Yes	N/A	N/A	Yes	Yes	Yes
11	Were the methods (including statistical methods) sufficiently described to enable them to be repeated?	Yes	Yes	Yes	Yes	Yes	Yes	Yes	Yes	Yes	Yes	Yes
Results												
12	Were the basic data adequately described?	Yes	Yes	Yes	Yes	No- No reporting of prioritisation of patients for whom no medication changes were proposed	Yes	Yes	Yes	Yes	Yes	Yes
13	Does the response rate raise concerns about non-response bias?	No	Don't know- response rate not reported	No	No	No	Don't know- response rate not reported	No	Don't know- response rate not reported	No	No	No
14	If appropriate, was information about non-responders described?	Yes	No	Yes	No	Yes	No	Yes	No	No	Yes in the parent study[25]<br><br>Characteristics of participants who were not included	Yes in the parent study[25]

For peer review only

											due to missing data, were described in this study	
15	Were the results internally consistent?	Yes	Yes	Yes	Yes	Yes	Yes	Yes	Yes	Yes	Yes	Yes
16	Were the results presented for all the analyses described in the methods?	Yes	Yes	Yes	Yes	No- No reporting of prioritisation of patients for whom no medication changes were proposed	Yes	Yes	Yes	Yes	Yes	Yes
Discussion												
17	Were the authors' discussions and conclusions justified by the results?	No- very small sample of GP's compared to patients therefore generalizable conclusions regarding concordance between doctors and patients cannot accurately be drawn from this study	Yes	Yes	Yes	Yes	Yes	Yes- Small sample size for quantitative aspect of study taken into account	No- very small sample size across patients and clinicians, meaning results are not generalizable	Yes	Yes	Yes

For peer review only

18	Were the limitations of the study discussed?	Yes	No	Yes	Yes	Yes	Yes	Yes	Yes	Yes	Yes	Yes
Other												
19	Were there any funding sources or conflicts of interest that may affect the authors' interpretation of the results?	No	No	No	No	No	No	No	No	No	No	No
20	Was ethical approval or consent of participants attained?	Yes	Yes	Yes	Yes	Yes	Yes	Yes	Yes	Yes	Yes	Yes

For peer review only

The Cochrane Collaboration's tool for assessing risk of bias in randomised controlled trials summary table		
Study	Junius-Walker et al [26]	
Bias	Authors' judgement	Support for judgement
Random sequence generation (selection bias)	Low risk	"Participating doctors were allocated 1:1 into the intervention and control group using random block sizes of four"
Allocation concealment (selection bias)	Unclear risk	No information given regarding any efforts to conceal the allocation sequence
Blinding of participants and researchers (performance bias)	Low risk	Participants were only informed of the procedures of their own trial arm.
Blinding of outcome assessment (detection bias)	Low risk	Participants were blinded to the pre-intervention importance ratings, when completing the final importance ratings.
Incomplete outcome data (attrition bias)	High risk	25 patients dropped out prior to baseline ratings and 5 further patients dropped out prior to final ratings, these patients were excluded from analysis, however intention to treat analysis cannot be carried out in this context due to the nature of the intervention
Selective reporting (reporting bias)	Low risk	Adequate reporting on all of the specified outcomes
Other bias	None detected	

For peer review only

References

1 Kuluski K, Gill A, Naganathan G, et al. A qualitative descriptive study on the alignment of care goals between older persons with multi-morbidities, their family physicians and informal caregivers. *BMC Family Practice* 2013;14:133.

2 Schoenberg NE, Leach C, Edwards W. "It's a toss up between my hearing, my heart, and my hip": prioritizing and accommodating multiple morbidities by vulnerable older adults, *J Health Care Poor Underserved* 2009;20:134-51.

3 Cheraghi-Sohi S, Morden A, Bower P, et al. Exploring patient priorities among long-term conditions in multimorbidity: A qualitative secondary analysis. *SAGE Open Medicine* 2013;1:2050312113503955.

4 Naik A.D., Martin L.A., Moye J., et al. Health Values and Treatment Goals of Older, Multimorbid Adults Facing Life-Threatening Illness. *J Am Geriatr Soc* 2016;64:625-31.

5 LINDSAY S. PRIORITIZING ILLNESS: LESSONS IN SELFa MANAGING MULTIPLE CHRONIC DISEASES, *CANADIAN JOURNAL OF SOCIOLOGY/CAHIERS CANADIENS DE SOCIOLOGIE* 2009;34:983.

6 Hansen H., Pohontsch N., van den Bussche H., et al. Reasons for disagreement regarding illnesses between older patients with multimorbidity and their GPs - a qualitative study. *BMC family practice* 2015;16:68.

7 Morris R.L., Sanders C., Kennedy A.P., et al. Shifting priorities in multimorbidity: A longitudinal qualitative study of patient's prioritization of multiple conditions. *Chronic Illness* 2011;7:147-61.

8 Elliott RA, Ross-Degnan D, Adams AS, et al. Strategies for coping in a complex world: adherence behavior among older adults with chronic illness, *Journal of General Internal Medicine* 2007;22:805-10.

9 Fried TR, McGraw S, Agostini JV, et al. Views of older persons with multiple morbidities on competing outcomes and clinical decision-making, *J Am Geriatr Soc* 2008;56:1839-44.

10 Turner J.P., Edwards S., Stanners M., et al. What factors are important for deprescribing in Australian long-term care facilities? Perspectives of residents and health professionals. *BMJ Open* 2016;6:Arte Number: e009781. ate of Pubaton: 2016.

11 Summeren JJ, Haaijer-Ruskamp FM, Schuling J. Eliciting preferences of multimorbid elderly adults in family practice using an outcome prioritization tool, *J Am Geriatr Soc* 2016;64.

For peer review only

12 Caughey G.E., Huynh E., Shakib S., et al. Influence of medication risks and benefits on patient and clinician preferences for treatment in multimorbidity: A discrete-choice experiment. 2017.

13 Zulman D.M., Kerr E.A., Hofer T.P., et al. Patient-provider concordance in the prioritization of health conditions among hypertensive diabetes patients. *Journal of General Internal Medicine* 2010;25:408-14.

14 Junius-Walker U, Stolberg D, Steinke P, et al. Health and treatment priorities of older patients and their general practitioners: a cross-sectional study. *Quality in primary care* 2011;19.

15 Fried TR, Tinetti ME, Iannone L, et al. Health outcome prioritization as a tool for decision making among older persons with multiple chronic conditions, *Arch Intern Med* 2011;171:1856-8.

16 Fried TR, Tinetti M, Agostini J, et al. Health outcome prioritization to elicit preferences of older persons with multiple health conditions, *Patient Educ Couns* 2011;83:278-82.

17 Moore A., Patterson C., Nair K., et al. Minding the gap: Prioritization of care issues among nurse practitioners, family physicians and geriatricians when caring for the elderly. *Journal of interprofessional care* 2015;29:401-3.

18 Van Summeren J.J.G.T., Schuling J., HaaijerRuskamp F.M., et al. Outcome prioritisation tool for medication review in older patients with multimorbidity: A pilot study in general practice. *British Journal of General Practice* 2017;67:e501-6.

19 Voigt I, Wrede J, Diederichs-Egidi H, et al. Priority setting in general practice: health priorities of older patients differ from treatment priorities of their physicians, *Croat Med J* 2010;51:483-92.

20 Caughey G.E., Tait K., Vitry A.I., et al. Influence of medication risks and benefits on treatment preferences in older patients with multimorbidity. *Patient Preference and Adherence* 2017;11:131-40.

21 Mantelli S, Jungo KT, Rozsnyai Z, et al. How general practitioners would deprescribe in frail oldest-old with polypharmacy—the LESS study, *BMC family practice* 2018;19:169.

22 Déruaz-Luyet A, N'Goran AA, Pasquier J, et al. Multimorbidity: can general practitioners identify the health conditions most important to their patients? Results from a national cross-sectional study in Switzerland, *BMC family practice* 2018;19:66.

For peer review only

23 Herzig L, Mueller Y, Haller DM, et al. Family practitioners' top medical priorities when managing patients with multimorbidity: a cross-sectional study, *BJGP open* 2019;3:bjgpopen18X101622.

24 DeruazLuyet A., Alexandra N'Goran A., Tandjung R., et al. Multimorbidity in primary care: Protocol of a national cross-sectional study in Switzerland. *BMJ Open* 2015;5:Arte Number: e009165. ate of Pubaton: 2015.

25 Deruaz-Luyet A, N'Goran AA, Senn N, et al. Multimorbidity and patterns of chronic conditions in a primary care population in Switzerland: a cross-sectional study, *BMJ Open* 2017;7:e013664,2016-013664.

26 Junius-Walker U, Wrede J, Voigt I, et al. Impact of a priority-setting consultation on doctor-patient agreement after a geriatric assessment: cluster randomised controlled trial in German general practices. *Quality in primary care* 2012;20.

For peer review only

Reporting checklist for systematic review and meta-analysis.

Based on the PRISMA guidelines.

		Reporting Item	Page Number
**Title**			
	#1	Identify the report as a systematic review, meta-analysis, or both.	1
**Abstract**			
Structured summary	#2	Provide a structured summary including, as applicable: background; objectives; data sources; study eligibility criteria, participants, and interventions; study appraisal and synthesis methods; results; limitations; conclusions and implications of key findings; systematic review registration number	2
**Introduction**			
Rationale	#3	Describe the rationale for the review in the context of what is already known.	3

For peer review only

Objectives	#4	Provide an explicit statement of questions being addressed with reference to participants, interventions, comparisons, outcomes, and study design (PICOS).	3
**Methods**			
Protocol and registration	#5	Indicate if a review protocol exists, if and where it can be accessed (e.g., Web address) and, if available, provide registration information including the registration number.	3
Eligibility criteria	#6	Specify study characteristics (e.g., PICOS, length of follow-up) and report characteristics (e.g., years considered, language, publication status) used as criteria for eligibility, giving rational	3,4
Information sources	#7	Describe all information sources in the search (e.g., databases with dates of coverage, contact with study authors to identify additional studies) and date last searched.	3
Search	#8	Present full electronic search strategy for at least one database, including any limits used, such that it could be repeated.	3, Appendix 1
Study selection	#9	State the process for selecting studies (i.e., for screening, for determining eligibility, for inclusion in the systematic review, and, if applicable, for inclusion in the meta-analysis).	4, Figure 1

For peer review only

Data collection process	#10	Describe the method of data extraction from reports (e.g., piloted forms, independently by two reviewers) and any processes for obtaining and confirming data from investigators.	4
Data items	#11	List and define all variables for which data were sought (e.g., PICOS, funding sources), and any assumptions and simplifications made.	3
Risk of bias in individual studies	#12	Describe methods used for assessing risk of bias in individual studies (including specification of whether this was done at the study or outcome level, or both), and how this information is to be used in any data synthesis.	4
Summary measures	#13	State the principal summary measures (e.g., risk ratio, difference in means).	N/A
Planned methods of analyis	#14	Describe the methods of handling data and combining results of studies, if done, including measures of consistency (e.g., I2) for each meta-analysis.	4,5
Risk of bias across studies	#15	Specify any assessment of risk of bias that may affect the cumulative evidence (e.g., publication bias, selective reporting within studies).	4
Additional analyses	#16	Describe methods of additional analyses (e.g., sensitivity or subgroup analyses, meta-regression), if done, indicating which were pre-specified.	N/A

Results

For peer review only

Study selection	#17	Give numbers of studies screened, assessed for eligibility, and included in the review, with reasons for exclusions at each stage, ideally with a flow diagram.	Figure 1
Study characteristics	#18	For each study, present characteristics for which data were extracted (e.g., study size, PICOS, follow-up period) and provide the citation.	5,6,7,8,9,10 (Table 1)
Risk of bias within studies	#19	Present data on risk of bias of each study and, if available, any outcome-level assessment (see Item 12).	Appendix 2
Results of individual studies	#20	For all outcomes considered (benefits and harms), present, for each study: (a) simple summary data for each intervention group and (b) effect estimates and confidence intervals, ideally with a forest plot.	N/A
Synthesis of results	#21	Present the main results of the review. If meta-analyses are done, include for each, confidence intervals and measures of consistency.	11,12,13,14,15
Risk of bias across studies	#22	Present results of any assessment of risk of bias across studies (see Item 15).	10
Additional analysis	#23	Give results of additional analyses, if done (e.g., sensitivity or subgroup analyses, meta-regression [see Item 16]).	N/A
**Discussion**			
Summary of Evidence	#24	Summarize the main findings, including the strength of evidence for each main outcome; consider their	16

For peer review only

		relevance to key groups (e.g., health care providers, users, and policy makers	
Limitations	#25	Discuss limitations at study and outcome level (e.g., risk of bias), and at review level (e.g., incomplete retrieval of identified research, reporting bias).	17
Conclusions	#26	Provide a general interpretation of the results in the context of other evidence, and implications for future research.	18
**Funding**			
Funding	#27	Describe sources of funding or other support (e.g., supply of data) for the systematic review; role of funders for the systematic review.	1

Notes:

- 8: 3, appendix 1
- 9: 4, Figure 1
- 18: 5,6,7,8,9,10 (Table 1)
- 21: 11,12,13,14,15

The PRISMA checklist is distributed under the terms of the Creative Commons Attribution License CC-BY. This checklist was completed on 13/11/2019

For peer review only

BMJ Open

Priorities of patients with multimorbidity and of clinicians regarding treatment and health outcomes: a systematic mixed studies review

Journal:	*BMJ Open*
Manuscript ID	bmjopen-2019-033445.R2
Article Type:	Original research
Date Submitted by the Author:	16-Dec-2019
Complete List of Authors:	Sathanapally, Harini; University of Leicester Diabetes Research Centre,<br>Sidhu, Manbinder S.; University of Birmingham,<br>Fahami, Radia; University of Leicester Diabetes Research Centre<br>Gillies, Clare; University of Leicester Diabetes Research Centre<br>Kadam , Umesh ; University of Leicester,<br>Davies, Melanie; University of Leicester Diabetes Research Centre<br>Khunti, Kamlesh; University of Leicester Diabetes Research Centre<br>Seidu, Samuel; University of Leicester Diabetes Research Centre
**Primary Subject Heading**:	General practice / Family practice
Secondary Subject Heading:	Patient-centred medicine, Communication
Keywords:	PRIMARY CARE, GENERAL MEDICINE (see Internal Medicine), QUALITATIVE RESEARCH

SCHOLARONE™
Manuscripts

I, the Submitting Author has the right to grant and does grant on behalf of all authors of the Work (as defined in the below author licence), an exclusive licence and/or a non-exclusive licence for contributions from authors who are: i) UK Crown employees; ii) where BMJ has agreed a CC-BY licence shall apply, and/or iii) in accordance with the terms applicable for US Federal Government officers or employees acting as part of their official duties; on a worldwide, perpetual, irrevocable, royalty-free basis to BMJ Publishing Group Ltd ("BMJ") its licensees and where the relevant Journal is co-owned by BMJ to the co-owners of the Journal, to publish the Work in this journal and any other BMJ products and to exploit all rights, as set out in our licence.

The Submitting Author accepts and understands that any supply made under these terms is made by BMJ to the Submitting Author unless you are acting as an employee on behalf of your employer or a postgraduate student of an affiliated institution which is paying any applicable article publishing charge ("APC") for Open Access articles. Where the Submitting Author wishes to make the Work available on an Open Access basis (and intends to pay the relevant APC), the terms of reuse of such Open Access shall be governed by a Creative Commons licence – details of these licences and which Creative Commons licence will apply to this Work are set out in our licence referred to above.

Other than as permitted in any relevant BMJ Author's Self Archiving Policies, I confirm this Work has not been accepted for publication elsewhere, is not being considered for publication elsewhere and does not duplicate material already published. I confirm all authors consent to publication of this Work and authorise the granting of this licence.

For peer review only

Priorities of patients with multimorbidity and of clinicians regarding treatment and health outcomes: a systematic mixed studies review

Harini Sathanapally[1], Manbinder Sidhu[2], Radia Fahami[1], Clare Gillies[1], Umesh Kadam[1], Melanie J Davies[1], Kamlesh Khunti[1] and Samuel Seidu[1]

1. *Diabetes research centre, College of Life Sciences, University of Leicester, UK*
2. *Health Services Management Centre, School of Social Policy, University of Birmingham, UK*

Corresponding author: Dr Harini Sathanapally (hs333@student.le.ac.uk)

Word count excluding titles, abstract, article summary, tables, figures and references: 5060

Author contributions: HS: Design of research question and methodology, data searching, data extraction, data analysis and manuscript development; MS: Design of methodology, data extraction, data analysis and manuscript development; RF: Data searching and data extraction; CG: Data analysis and manuscript development; UK: Data analysis and manuscript development; MJD: Data analysis and manuscript development; KK: Design of research question and methodology, manuscript development; SS: Conception of the idea for this review, design of research question and methodology, data extraction, data analysis and manuscript development. All authors have approved the final manuscript.

Funding: Harini Sathanapally is funded by the NIHR academic clinical fellowship award

Competing interests: None declared

Patient consent for publication: Not required

Data sharing statement: The majority of the data extracted and analysed as part of this review has been included in this article and associated appendices. Any further data can be acquired from the corresponding author by request.

For peer review only

ABSTRACT

Objectives: To identify studies that have investigated the health outcome and treatment priorities of patients with multimorbidity, clinicians, or both, in order to assess whether the priorities of the two groups are in alignment, or whether a disparity exists between the priorities of patients with multimorbidity and clinicians.

Design: Systematic review

Data sources: MEDLINE, EMBASE, CINHAL and Cochrane databases from inception to May 2019 using a pre-defined search strategy, as well as reference lists containing any relevant articles, as per PRISMA and Cochrane guidelines.

Eligibility criteria: We included studies reporting health outcome and treatment priorities of adult patients with multimorbidity, defined as suffering from two or more chronic conditions, or of clinicians in the context of multimorbidity, or both. There was no restriction by study design, and studies using quantitative and/or qualitative methodologies were included.

Data synthesis: We used a narrative synthesis approach to synthesise the quantitative findings, and a meta-ethnography approach to synthesise the qualitative findings.

Results: Our search identified twenty four studies for inclusion, which comprised of twelve quantitative studies, ten qualitative studies and two mixed-methods studies. Twelve studies reported the priorities of both patients and clinicians, ten studies reported the priorities of patients and two studies reported the priorities of clinicians alone. Our findings have shown a mostly low level of agreement between the priorities of patients with multimorbidity and clinicians. We found that prioritisation by patients was mainly driven by their illness experiences, whilst clinicians focused on longer term risks. Preserving functional ability emerged as a key priority for patients from across our quantitative and qualitative analyses.

Conclusion: Recognising that there may be a disparity in prioritisation and understanding the reasons for why this might occur, can facilitate clinicians in accurately eliciting the priorities that are most important to their patients and delivering patient-centred care.

KEY WORDS: Patient-centred care, Shared decision-making, Multimorbidity

ARTICLE SUMMARY

Strengths and limitations

- This is the first systematic review to assimilate and compare the findings of existing literature on the health outcome and treatment priorities of both clinicians treating and patients living with multimorbidity.
- We have included papers using both qualitative and quantitative methodologies and have been able to explore patterns and relationships in the findings, thus creating a comprehensive and well-rounded systematic review.
- Our findings facilitate clinicians in understanding both *how* and *why* the health outcome and treatment priorities of their patients with multimorbidity might differ from their own priorities.
- Meta-analysis of the quantitative studies was unfeasible as there was a large variation in the tools used to ascertain priorities, and we have attempted to mitigate this by using a well-described and transparent method of narrative synthesis.
- A number of our included quantitative studies did not use pre-validated tools to ascertain priorities, leading to a risk of measurement bias.

For peer review only

INTRODUCTION

Multimorbidity, defined as the co-existence of two or more long-term conditions [1] is a global problem [2], which has become the norm across high-income countries [2, 3][4, 5] and becoming increasingly prevalent in middle and low-income countries [6][7][2]. Guidelines for the management of chronic diseases are often single disease-orientated, and can lead to confusion and complications when applied to patients with multimorbidity [8]. Patients with multimorbidity have an increased risk of adverse drug-related events as a result of high levels of polypharmacy and receiving un-coordinated care from multiple healthcare providers [9]. These patients have a poorer health-related quality of life [10], poorer functional status [11] and greater psychological distress [12]. As a result, understanding and finding better strategies to facilitate the management of patients with multimorbidity has been identified as a priority for health research [13] .

Key to the effective management of multimorbidity is using patient-centred care and shared decision-making to set management goals that are acceptable to both the patient and the clinician [14]. Incorporating the priorities of patients in relation to treatments and health outcomes is integral to this process [15-17]. However, previous research has shown that whilst doctors recognise the importance of eliciting and incorporating the priorities of their patients with multimorbidity, they do not always engage with this process in real world settings, and find eliciting patients' priorities to be difficult [18] [19]. Previous research, completed in a single disease context, has shown that the treatment and health outcome priorities of patients and clinicians can differ [20-22], and some studies have highlighted a gap between what doctors' perceive to be the priorities of their patients, and the actual priorities of their patients [23-25].

This systematic review aims to identify studies that have investigated the health outcome and treatment priorities of patients with multimorbidity, clinicians, or both, in order to assess whether the priorities of the two groups are in alignment, or whether there is a disparity between the priorities of patients with multimorbidity and clinicians.

METHODS

Search strategy

This systematic review has been registered on PROSPERO (ID: CRD42018076076). A comprehensive search strategy (Appendix 1), was developed using guidance for best practice [26] and input from academic librarians at the University of Leicester. The search strategy was used to search MEDLINE, EMBASE, CINHAL and COCHRANE databases from inception to May 2019, as well as searching reference lists for any relevant articles based on PRISMA and Cochrane guidelines [26-28]. We undertook a scoping search using google scholar using our key terms (Patient*; Priorit*; Clinician, Physician, Doctor, General-practitioner, Family-practitioner; Multimorbidit*; Multi morbid*) to identify relevant grey literature. Citations were stored using Refworks. We have presented our process of article selection in Figure 1.

We included studies reporting the health outcome and treatment priorities of adult patients with multimorbidity [1] and/or clinicians, in relation to patients with multimorbidity. Studies which did not specify the definition of multimorbidity as "two or more chronic conditions" [1] in their inclusion criteria, but had a sample patients representative of being diagnosed with multimorbidity (i.e. with a minimum of two chronic conditions), were also included. There was no restriction by study design,

For peer review only - http://bmjopen.bmj.com/site/about/guidelines.xhtml

and we included studies using quantitative and/or qualitative methodologies. We excluded studies not published in English language, studies with participants aged under 18 years, and studies focusing on a single disease area.

Patient and Public Involvement

Patient and public involvement was not applicable in the design, conduct or reporting of this review.

Study selection

The titles and abstracts of all articles identified by the literature search were assessed independently and in duplicate by two reviewers (HS and RF). Studies that did not meet inclusion criteria were discarded. Full text of selected articles were retrieved and assessed to determine if they met the inclusion criteria, and those studies which met the inclusion criteria were included in the review. Any discrepancies regarding eligibility of an article were discussed, and consensus reached with MS and SS.

Methodological quality assessment and data extraction

Data was extracted using standardised data extraction forms by a single reviewer (HS), and these were checked independently for accuracy by a second reviewer (SS). The reported health outcome and treatment priorities of study participants were the key outcomes that were extracted.

Quality assessment was carried out in parallel with the data extraction process. For the quantitative studies, due to the heterogeneity of study design, we used the AXIS tool for assessment for the cross-sectional studies [29], the Newcastle-Ottawa scale for assessment of the longitudinal observational and cohort studies [30], and the Cochrane collaboration's risk of bias tool for assessment of randomised controlled trials [31]. For the qualitative studies, we used the CASP checklist for appraisal of qualitative research [32]. For the two mixed-methods studies, we used the AXIS tool [29] to assess the quantitative aspects of the study (both cross-sectional in study design), and the CASP checklist for qualitative research [32], to assess the qualitative aspects of these studies.

Data synthesis

We decided *a priori* not to carry out a meta-analysis due to the heterogeneity of the quantitative studies. Therefore, we have taken a narrative synthesis approach, described by Popay et al [33] to synthesise our quantitative findings. Our approach consists of three key steps:

1) *Development of a preliminary synthesis* in which study characteristics and descriptions are collated and findings presented in a summary table

2) Exploring relationships in the data between study characteristics and their findings, as well as between the reported findings across different studies with explanations considered where relationships were identified.

c) *Assessing the robustness of the synthesis using* quality assessment tools to guide conclusions and identify directions for clinical practice.

Qualitative studies were synthesised using a meta-ethnography approach [34, 35], which consisted of careful reading of the papers, extracting information regarding the context of the study and findings. *Key concepts* arising from each paper were also identified, with preservation of the terminology used by the authors where possible to ensure accurate representation of the findings of

For peer review only - http://bmjopen.bmj.com/site/about/guidelines.xhtml

the original studies. The key concepts across the papers were then *translated* using a table summarising the studies, their findings in relation to the key concepts and the *second order* interpretations of the authors, which enabled the exploration of any relationships and differences. The translations were then synthesised using a table containing the *first order* and *second order* interpretations for the key concepts across the studies, which then led to the development of further, *third order* interpretations by reviewers [34, 35].

From the results of our narrative synthesis of the quantitative studies and meta-ethnography of the qualitative studies, we considered how the findings of the two syntheses complement one another, particularly where our qualitative findings may provide possible explanations for our quantitative findings. The outcome of this process is described in the discussion section.

RESULTS

Overall study characteristics

Our search resulted in the identification of 24 studies for inclusion, which comprised of 12 quantitative studies, 10 qualitative studies and two mixed-methods studies. The characteristics of all of the included studies are described in Table 1. The included studies had all been conducted in high income developed countries, including Canada [36, 37], USA[38-44], Netherlands[45, 46], Australia[47, 48], UK[49-51], Germany [52-55]and Switzerland [56-58]. Sample sizes ranged from 15 to 1169 patients and 5 to 92 clinicians in the quantitative studies, and 15 to 146 patients and 4 to 19 clinicians in the qualitative studies.

Author and year of publication	Setting	Study type	Study aims	Target group and number of participants (n)	Outcomes measured
**QUANTITATIVE**					
**Health outcome priorities**					
Fried et al, 2011 [39]	USA- 3 senior centres and 1 assisted living facility	Quantitative: Cross-sectional study	*To explore the use of a simple tool to elicit older persons' health outcome priorities*	All volunteers included (n=357)	The prioritisation by participants of 4 universal health outcomes, namely:<br>-keeping alive<br>- maintaining independence<br>- reducing or eliminating pain<br>-reducing or eliminating other symptoms
Fried et al , 2011, [40]	USA- recruited from participants in a larger study, where they had been recruited from age-aggregated community housing [59]	Quantitative: Cross-sectional survey	*To determine the feasibility of using a simple tool to elicit the preferences of older persons based on their prioritization of universal outcomes*	Patients aged 65 and over with a known diagnosis of hypertension or use of anti-hypertensive medications, and having a known risk of falls (n=81)	> Rankings given by participants to 4 universal health outcomes in the outcome prioritisation tool:<br>--keeping alive<br>- maintaining independence<br>- reducing or eliminating pain<br>-reducing or eliminating other symptoms<br>> Feasibility of the use of outcome prioritisation tool

For peer review only - http://bmjopen.bmj.com/site/about/guidelines.xhtml

Mantelli et al, 2018[57]	Switzerland- General practitioners working in Switzerland who had previously taken part in case-vignette studies	Quantitative: cross-sectional survey	*To determine whether, how and why GPs de-prescribe in frail oldest-old patients with multimorbidity and polypharmacy, and to identify factors that influenced their decision to de-prescribe*	General Practitioners (n=157)	- Percentage of GPs willing to de-prescribe at least one medication in the case of frail older patients with CVD and compared to frail older patients without CVD<br>- Reasons for de-prescribing<br>- Importance ratings given to factors influencing decision to de-prescribe
Van Summeren et al, 2017 [46]	Netherlands- General practice centres	Quantitative: Cross-sectional and implementation study	*To determine proposed and observed medication changes when using an outcome prioritisation tool during a medication review in older patients with multimorbidity and polypharmacy. A secondary aim was to explore the relationship between the prioritized health outcome of patients and the type of medication change, such as a stop, a dose adjustment, or a switch.*	Patients aged 69 or over with two or more chronic diseases (one of which had to be cardiovascular disease) and daily use of five or more medications. (n=59)<br><br>General practitioners (n=17)	>Patients' priority rankings of the four health outcomes in the outcome prioritisation tool:<br>-Maintaining independence<br>-Remaining alive<br>-Reducing other symptoms<br>-Reducing pain<br><br>>Medication changes proposed by the GP, and observed in the patient records following incorporation of the priority rankings given by patients, into a medication review consultation.
Van Summeren et al, 2016 [45]	Netherlands- General practice centres	Mixed-methods: Cross-sectional survey pilot and qualitative interviews to assess acceptability (semi-structured and in-depth)	*To explore whether an outcome prioritization tool (OPT) is appropriate in the context of medication review in family practice, focusing on its acceptability and practicality*	Patients aged 69 or over with two or more chronic diseases (one of which had to be cardiovascular disease) and daily use of five or more medications (n=60)<br><br>General practitioners (n=13)	>Patients' prioritisation of the four domains of the outcome prioritisation tool:<br>-Maintaining independence<br>-Remaining alive<br>-Reducing other symptoms<br>-Reducing pain<br><br>> Family practitioners views on the acceptability and practicality of using the outcome prioritisation tool for medication review
**Problem-based priorities**					
Junius-Walker et al 2012 [52]	Germany- General practice centres	Quantitative: Randomised controlled trial	*To investigate whether a structured priority-setting consultation reconciles the often-differing doctor–patient views on the importance of problems.*	Patients aged 70 or over (n=317)<br><br>General practitioners (n=40)	-Baseline importance rankings given by patients and clinicians to a list of problems generated from a geriatric assessment for each patient.<br>- Importance rankings given again after a structured consultation incorporating the baseline problem list and importance rankings and degree of reconciliation in

For peer review only - http://bmjopen.bmj.com/site/about/guidelines.xhtml

					doctor- patient agreement after the structured consultation
Junius-Walker et al, 2011[53]	Germany- General practice centres	Quantitative: Cross-sectional survey	*To gain insight into setting individual priorities with older patients using a priority definition that was coherent to the patients' life and doctors' work context*	Patients aged 70 or over and living at home (n=123)<br><br>General practitioners (n=11)	Importance rankings given by patients and clinicians to a list of problems generated from a geriatric assessment for each patient.
Voigt et al, 2010 [54]	Germany- General practice centres	Quantitative: Cross-sectional survey	*To ascertain health priorities of older patients and treatment priorities of their general practitioners (GP) on the basis of a geriatric assessment and to determine the agreement between these priorities.*	Patients aged 70 or over and at least one contact with the general practitioner in the preceding 3 months (n= 35)<br><br>General practitioners (n=9)	-Importance rankings given to problems generated from a geriatric assessment by patients and clinicians<br>-Degree of agreement between patients and clinicians on the above
**Condition-focused priorities**					
Moore et al, 2014 [36]	Canada- Databases of all practising nurse practitioners, family practitioners and geriatricians in Ontario	Quantitative: Cross-sectional survey	*To quantify how family physicians, nurse practitioners and geriatricians prioritize syndromes, diseases and conditions when caring for seniors*	Nurse practitioners (n=68)<br><br>Family practitioners (n=84)<br><br>Geriatricians (n=27)	Frequency and importance rankings given by family practitioners, nurse practitioners and geriatricians to 41 health issues known to arise in elderly patients
Zulman et al, 2010 [44]	USA- Scheduled primary care visit for patients at 9 veteran affairs facilities	Quantitative: Prospective cohort study	*To understand patterns of patient-provider concordance in the prioritization of health conditions in patients with multimorbidity*	Patients with diabetes and hypertension who had their primary diabetes care provider enrolled in the study (n = 1169)<br><br>Primary care providers i.e. physicians, physician assistants or nurse practitioners (n= 92)	-Patient rankings given in terms of their most important health concerns and providers rankings in terms of conditions most likely to affect each patient's outcomes<br>-Concordance between the importance ratings of patient-provider "pairs"
Herzig et al, 2019 [56]	Switzerland- Primary data was from "Multimorbidity in Family medicine" study [60].<br><br>Patients enrolled by General practitioners during scheduled consultations.	Quantitative: Cross-sectional survey	*To describe FPs' medical priority ranking of conditions relative to their prevalence in patients with multimorbidity*	Patients suffering from at least 3 of 75 chronic conditions on a pre-defined list (based on the International classification of primary care 2 (n=888)<br><br>General Practitioners (n=100)	Importance rankings given by family practitioners to the list of chronic conditions that each patient had on the day of their inclusion in the study
Déruaz-Luyet et al, 2018 [58]	Switzerland- Primary data was from "Multimorbidity in Family	Quantitative: Cross-sectional survey	*To evaluate whether GPs could identify the condition that their patients*	Patients suffering from at least 3 of 75 chronic conditions on a pre-defined list (based on the International	Whether there is agreement between what patients considered to be their most important health condition and what GPs thought patients

For peer review only

For peer review only - http://bmjopen.bmj.com/site/about/guidelines.xhtml

	medicine" study [60].<br><br>Patients enrolled by General practitioners during scheduled consultations.		*with multimorbidity considered most important.*	classification of primary care 2, and receiving follow-up from their GP for at least the preceding 6 months<br>(n= 572 for main analysis, 585 for sensitivity analysis)<br><br>General Practitioners (n=100)	considered to be their most important health condition
**Treatment priorities**					
Caughey et al, 2017 [47]	Australia- Multi-disciplinary ambulatory consulting service clinics at tertiary teaching hospitals	Mixed-methods: Structured quantitative interviews with patients then semi-structured qualitative interviews with patients and clinicians	*To investigate how older patients with multimorbidity balance the benefits and harms associated with medication for prevention of CVD, and in the presence of competing health outcomes.*<br>*To investigate the factors that clinicians consider when making treatment decisions for older patients with multimorbidity.*	Patients aged 65 or older with 2 or more chronic conditions (n=15)<br><br>Clinicians (n=5)	-Patient willingness to take a medication when presented with different scenarios with variable degree of benefit, impact on daily living, adverse outcomes and impact on other comorbid conditions<br><br>-Patient-reported data during semi-structured interviews where they were asked about their treatment preferences, medication effects and shared decision making<br><br>-Clinician reported data during semi-structured interviews on treatment decisions, patient preferences and polypharmacy.
**QUALITATIVE**					
Kuluski et a, 2013 [37]	Canada- A Family Health Team in Ontario	Qualitative: Semi-structured interviews	*To examine patient goals of care from the perspectives of older persons with multi-morbidities, their family physicians and informal caregivers (i.e., family member or friend who provides ongoing support) and then examine the extent of alignment between these three perspectives*	Patients aged 65 or older with a diagnosis of at least two chronic health conditions (n=28)<br><br>Informal Caregivers of included patients (n=28)<br><br>Family physicians (n=4)	>Patient, caregiver and physician reported data on goals of care for the patients<br><br>>Degree of alignment of goals of care across patient, caregiver and physician "triads"
Schoenberg et al, 2009 [38]	USA- Senior centres, Low income senior housing complexes, churches and a civic meeting hall	Qualitative: In-depth interviews	*To understand how vulnerable older adults with multimorbidity prioritize and manage their chronic conditions*	Patients aged 55 or older with a diagnosis of at least two chronic illnesses, from low-income backgrounds (n= 41)	Patient-reported data from in-depth interviews, regarding their medical history, self-care procedures, patient prioritisation by means of health-related areas of worry and health-related "expenditures" in terms of money, time and need for reliance on others.

For peer review only - http://bmjopen.bmj.com/site/about/guidelines.xhtml

Fried et al, 2008 [41]	USA- Senior centres, Doctors' practices and a congregate housing site	Qualitative: Focus groups	*To examine the ways in which older persons with multiple conditions think about potentially competing outcomes, in order to gain insight into how processes to elicit values regarding these outcomes can be grounded in the patient's perspective*	Patients aged 65 or older and were taking 5 or more medications (participants also had a minimum of 3 chronic conditions)	Patient-reported data regarding their perceptions of the interactions between their different illnesses and treatment regimens, goals of treatment and decisions regarding treatment
Naik et al, 2016 [42]	USA- Qualitative data from the VETCARES study [61] , in which participants recruited from the VA tumour registry	Qualitative: Open-ended questions as part of mixed methods interviews which also included structured questions	*To identify a taxonomy of health-related values that frame goals of care of older adults with multimorbidity who recently faced cancer diagnosis and treatment*	Veterans with a diagnosis of head and neck, gastric, oesophageal, or colorectal cancer, and diagnosis fell one month prior to the study's opening eligibility window (6 months) (n=146)	Patient-reported data regarding their priorities or concerns regarding their future healthcare decisions
Elliott et al, 2007 [43]	USA- Harvard Pilgrim Health Centre, a HMO (health maintenance organisation) in New England	Qualitative: Semi-structured interviews	*To explore how older adults with multiple illnesses make choices about medicines*	Patients taking more than three medicines with purposive sampling to reflect symptomatic comorbidities and asymptomatic comorbidities and mental health issues (participants had a minimum of 3 comorbidities) (n=20)	Patient-reported data regarding beliefs about medicines, medicine-taking behaviour, historical vs potential choices between different medicines, and factors influencing these choices
Turner et al, 2016 [48]	Australia- Long term care facilities in South Australia	Qualitative: Nominal group technique	*To use nominal group technique to generate then rank factors that general medical practitioners, nurses, pharmacists and residents or their representatives perceive are most important when deciding whether or not to de-prescribe medication*	Residents/representatives of residents (n=11)<br><br>General Practitioners (n=19)<br><br>Nurses (n=12)<br><br>Pharmacists (n=14)	-Generated factors important for de-prescribing according to residents/resident representatives, general practitioners, nurses and pharmacists<br><br>-Priority rankings given by groups containing representatives from all of the above, to the list of priorities generated previously.
Lindsay, 2009 [49]	UK- Participants recruited from CHD registries in Greater Manchester as part of a larger RCT[62]	Qualitative: Focus groups and two interviews	*To use the concepts of "chronic illness trajectory" and "biographical disruption" to examine how patients self-manage multiple chronic conditions and especially how they prioritize their conditions*	Participants from the parent study who had more than one chronic condition (i.e. at least two) (n=53)	Patient-reported data regarding how they prioritised their multiple conditions, what strategies they used to cope with their conditions and barriers in being able to manage their illnesses

For peer review only - http://bmjopen.bmj.com/site/about/guidelines.xhtml

Cheraghi-Sohi et al, 2013 [50]	UK- secondary analysis of qualitative data from four other studies [63-66]	Qualitative: In-depth interviews	*To explore how and why people with multimorbidity prioritise some long-term conditions over others and what the potential implications may be for self-management activity, and in turn, suggest how such information may help clinicians negotiate the management of multimorbidity patients*	Participants from original studies who had two or more long term conditions, and had given data regarding prioritisation (n=41)	Patient-reported data pertaining to prioritisation of their long term conditions
Morris et al [51]	UK- General Practices in North-West England	Qualitative: Semi-structured interviews	*To examine what influences self-management priorities for individuals with multiple long-term conditions and how this changes over time*	Patients with more than one chronic condition and at least one of COPD, IBS or Diabetes (n=21)	Patient-reported data on management strategies and experiences with primary health care, and data from follow-up interviews on any changes in their illness management.
Hansen et al, 2015 [55]	Germany- Participants recruited from the "Multicare cohort study" [67]	Qualitative: Focus groups	*To identify reasons for disagreement regarding illnesses between patients and their GPs*	Patients who had 3 or more chronic conditions from a list of 29 conditions (n=21)<br><br>General Practitioners of the recruited patients (n=15)	Data from separate focus groups for patients and clinicians in which any communication problems and reasons for disagreement between patients and clinicians were explored

Table 1 Characteristics of all of the included studies in order of reference

Summary of quality assessment

The outcome of quality assessment based on each of the afore-mentioned tools is summarised in Appendix 2. The majority of the quantitative studies were cross-sectional in design [36, 39, 40, 45, 46, 53, 54, 56-58] [47], including the quantitative elements of the two mixed-methods studies. The other studies included one cohort study [44] and one randomised controlled trial [52]. The cross-sectional studies were of moderate quality, with a number of studies having small sample sizes [40, 45, 46, 54]. The sample sizes of clinicians in most of the cross-sectional studies were particularly small, ranging from of 9 to 157 clinicians [45, 46, 54, 57], which impacts upon the generalisability and application of their findings. We noted that a number of the studies did not use pre-validated questions and tools to ascertain priorities [36, 54, 56-58], leading to a degree of subjectivity in the way in which priorities were ascertained, and the risk of measurement bias which again impacts on the generalisability of their findings.

The majority of the qualitative studies, including the qualitative aspects of the two mixed-methods studies, used interviews for data collection (n=8). Two studies used focus groups [41, 55], one study used a combination of focus groups and interviews [49] and one study used the nominal group technique [48]. The qualitative studies were of good quality, with appropriate use of qualitative methodology and transparent descriptions of the data analysis processes. Three studies only gave a limited description of their analytic process [47-49], with two of these studies [47, 48] and one mixed-methods study [45], not presenting any quotes.

For peer review only

QUANTITATIVE SYNTHESIS

Within our quantitative synthesis, we found that the studies focused either on the overall state of the patients' health, the problems posed by different chronic disease groups, or the patients' treatment regimens. Some of the quantitative studies elicited patient and/or clinician priorities as part of an intervention [52] [46]. Therefore, in order to reduce the risk of bias from the interventions, we included only the pre-intervention results from these studies.

Health outcome priorities

Four studies reported patient priorities of overall health outcomes using a "health outcome prioritisation tool" [39, 40, 45], which is a visual analogue scale requiring the following health outcomes to be given a score out of 100: "Maintaining independence"; "Staying alive"; "Pain relief"; "Symptom relief". Maintaining independence was the outcome that had the highest importance after a pooling of the *most important* rankings from the four studies, followed by "Staying alive" (Table 2). For clinicians' priorities, one study reported that 98% of a sample of 157 general practitioners identified the "quality of life for the patient", and 96% identified the "life expectancy of the patient", as the most important factors in influencing their clinical decision-making to de-prescribe for elderly, patients with multimorbidity [57].

Study	*Health outcome prioritisation as a tool for decision making among older persons with multiple chronic conditions*[39]	*Health outcome prioritisation to elicit preferences of older persons with multiple health conditions*[40]	*Outcome prioritisation tool for medication review in older patients with multimorbidity: A pilot study in general practice*[46]	*Eliciting Preferences of multi-morbid Elderly Adults in Family Practice Using an Outcome Prioritisation Tool*[45]	*Aggregate ranking as most important (%)*
**Maintaining independence**	270 (75.6%)	34 (42.0%)	7 (36.8%)	19 (35.8%)	330 (64.7%)
**Staying alive**	40 (11.2%)	22 (27.2%)	6 (31.6%)	18 (34.0%)	86 (16.9%)
**Pain relief**	26 (7.3%)	17 (21.0%)	1 (5.3 %)	6 (11.3%)	50 (9.8%)
**Symptom relief**	21 (5.9%)	8 (9.8%)	5 (26.3%)	10 (18.9%)	44 (8.6%)
**Total number of participants**	357	81	19[a]	53	510

Table 2-Summary of most important rankings for studies using the Outcome Prioritisation Tool
a= although there were 59 patients included in this study [46] *priorities were only reported for 19 patients*

Priorities based on health problems

Three studies reported patient and general practitioners' priorities based on various health *problems*, following a geriatric assessment [52-54]. These problems were then categorised into domains, and the importance rankings for each of the domains were presented. Problems in the domains of "Social" "Mood" and "Function" recurrently featured in the top four of the most highly

For peer review only - http://bmjopen.bmj.com/site/about/guidelines.xhtml

ranked priorities by patients across all three studies. In terms of the importance rankings by clinicians, problems in the domains of “Mood” and “Function” also featured in the top four importance rankings across all three studies, whilst “Social” problems were rated highly in one study [53] and problems in the domain of “Medication” were ranked highly in the other two studies [52, 54]. Interestingly, the authors in one study[53] found that patients feeling “Emotionally affected” was the strongest predictor for a problem being rated as important (OR 11.1 CI 6.73 to 18.33), whereas “Poor prognosis” was the strongest predictor for clinicians (OR 6.39 CI 4.61 TO 8.87)

Condition-focused priorities

Two studies reported patient priorities in relation to specific conditions or groups of conditions [44, 58], in the context of multimorbidity. Zulman et al. reported that “Diabetes/glycaemic control” was most frequently ranked as “most important”, with “Hypertension” coming second [44]. However, the sample of patients included in this study were all diabetic, hypertensive patients. Deruaz-luyet et al. found that musculoskeletal conditions including back pain, were most frequently reported to be the most important conditions for their patients, however endocrine/metabolic conditions (including obesity) were second and cardiovascular conditions were third [58].

Three studies reported condition-focused priorities of clinicians in the context of multimorbidity. Herzig et al. reported the priorities of general practitioners alone [56], and found that “multiple sclerosis”, “mental retardation”, and “bronchus lung neoplasm” were all highly prioritised by their participants. Zulman et al reported the priorities of “primary care providers” who consisted of physicians, physician assistants or nurse practitioners [44], and found that diabetes was the top priority for primary care providers, with hypertension coming second, in alignment with their previously described patient priorities [44]. Moore et al. examined the priorities of different types of clinicians, including family physicians, geriatricians and nurse practitioners [36], and as with Zulman et al., found that diabetes was the top priority for family physicians and also nurse practitioners, whereas dementia was the top priority for geriatricians [36]. In addition, heart failure, atrial fibrillation and hypertension formed three of the top five conditions considered to be most important by the family practitioners in the study [36].

Treatment priorities

As part of a study to examine the influence of the risks and benefits of medications on treatment preferences of patients, Caughey et al. also examined the priorities of patients in the face of “competing outcomes” [47]. They found that 80% of participants would not be willing to take medication to reduce “joint pain”, if the medication increased their risk of a myocardial infarction by 10%. However, this was deduced from a sample of only 15 patients [47].

Agreement between patients and clinicians

Five of the included studies investigated the level of agreement in priority rankings between patients and their clinicians [44, 52-54, 58]. Three studies reported a low level of agreement between patient and clinicians’ priority rankings [52-54]. Two of these studies used a Cohen’s Kappa calculation to estimate the degree of agreement between the importance ratings of patients and clinicians, and the values of which were 0.18 and 0.11 respectively, indicating “slight agreement” after allowing for chance [53][54]. One study used a weighted kappa calculation to measure the degree of agreement, which, at a pre-intervention point in this study, was low at 6% [52].

For peer review only - http://bmjopen.bmj.com/site/about/guidelines.xhtml

Two studies reported that there was a “high” level of agreement [44, 58]. Deruaz-Luyet et al. found that in the case of 54.9% (n=314) of their patients, the condition that their GP had considered to be either the first or second most important, was in the same disease-group as the condition that the patient considered to be most important [58].

Zulman et al. reported that 60% of “patient-provider pairs” had a “high concordance”, meaning that the same three conditions had been rated as top three priorities by both parties, or that two of the same conditions had been rated in the top three priorities by both parties [44]. In this case, given that the sample of patients were all diabetic and hypertensive could have led to a narrowing of the range of chronic diseases across the sample, which in turn could have led to an increased likelihood of agreement. However, the participant characteristics reported by the authors state that the patients had a mean of eight health conditions (SD 3.00), suggesting that the patients did not have a narrow range of chronic diseases. Furthermore, the questions posed to patients and providers were phrased differently, in that providers were asked to choose the top three most important medical concerns “that are likely to affect health outcomes for this patient”, whereas patients were asked to choose their top three most important health concerns. The authors acknowledge this in their paper, and justify this difference as being due to their aim of exploring the concordance in priorities about the “most important problems facing the patient”, rather which problems “providers thought the patient would have prioritised”, which, they argue, is a different concept to their aim [44].

QUALITATIVE SYNTHESIS

Whilst our quantitative synthesis allowed us to investigate *which* health outcomes, diseases or treatments were important to patients with multimorbidity and their clinicians, our qualitative analysis enabled us to explore *how* prioritisation occurs. Below, we describe the key findings from our qualitative analysis.

Mechanisms of prioritisation

In the qualitative studies that approached prioritisation from a disease-specific perspective, patients were able to identify an illness as their main priority [49, 50]. For many patients, prioritisation appeared to be driven by their experience of the illness, which formed part of its “meaning as consequence” [50] as phrased by Cheraghi-Sohi et al. The ‘consequences’ of an illness consisted of the *impact* that the illness was having on the patients’ everyday lives, which included functional limitation and the symptomatic burden of the illness, including its “unpredictability” (Table 3) [49]. For others, prioritisation appeared to be driven by their perception of the risk now and in the future with respect to functional deterioration and mortality.

In other studies, patients framed their priorities between *quality of life vs length of life* (Table 3) [42]. Patients in the study by Naik et al who were adults with multimorbidity and suffering from cancer, prioritised “quality of life” more highly than “length of life” [42]. This was also reflected in the findings of Fried et al., who found that when considering medication with competing outcomes in terms of extending life compared to quality of life, participants appeared to prioritise preserving quality of life [41].

Van Summeren et al. found that prioritisation was “difficult” when there was no “specific need” for a treatment decision to be made [45]. This concept of a difference in prioritisation based on hypothetical, or experiential levels, was also shared in the findings of Elliott et al [43] and Fried et al [41].

For peer review only - http://bmjopen.bmj.com/site/about/guidelines.xhtml

Where clinicians' perspectives were explored alongside patients, clinicians reported that exploring patients' priorities was "extremely important" when managing "competing interests" [47] and beneficial in providing patient-centred care [45]. Some clinicians in the mixed-methods study carried out by Van Summeren et al. reported that exploring their patients' priorities allowed them to have a "deeper understanding" of the patient, helped with making patient-centred treatment decisions and advance care planning (Table 3) [45]. However, other clinicians in the same study found exploring patient priorities to be difficult due its "novelty" and the fact that it represented a change to their usual consultations [45].

	Concept	Examples from included studies
Mechanisms of prioritisation	Unpredictability of symptoms	"My final issue is diverticulitis. In many ways that is the thing that makes the most impact on my life because of the unreliability of it. You make plans to do something to go somewhere and at the last minute you don't dare leave the house because you don't leave the loo. In itself it's not an important medical issue. It's the social problem more than anything else." – Lindsay et al [49]
	Quality of life vs length of life	"If you don't feel good, you can't take care of yourself and you have to depend on somebody else, what's the good of living another 10 years?"- Fried et al [41]
	Facilitating clinicians' decision making	"In future, I'll be happier to be more decisive in keeping an eye on what we do and do not do as regards this patient." Van Summeren et al [45]

Table 3- Examples from included studies for key concepts relating to mechanisms of prioritisation

Factors influencing prioritisation:

Our analysis revealed that there were a number of factors that appeared to influence how both patients and clinicians arrived at their priorities, and which priorities they chose.

i. Functional ability

Preserving functional ability as a priority for patients was a dominant concept across the majority of the qualitative studies [37, 38, 49, 51] [42][47][41]. Preserving independence emerged as the most significant reason for prioritising functional ability for patients, and maintaining the ability to engage in activities of daily living, mobility, maintaining cognitive ability and wanting to avoid being a "burden" or lacking social support to help them cope with functional deterioration (Table 4) [38, 49, 50].

Conditions which caused limitation to patients' ability to self-manage their health conditions, led to a "tension" between the patients' expectations of themselves and what they were physically able to do [51]. Lifestyle management, particularly reduced ability to exercise and the adverse impact of this on weight, was cited as part of patients' ability to self-manage [49].

Maintaining patients' functional ability was reported as a priority by some clinicians [37] [47]. Clinicians considered the wider implications of the patients' functional deterioration, particularly cognitive deterioration, and spoke of wanting to reduce the risk of "burnout" for the patients' family members/caregivers [37].

ii. Mortality

Reducing the risk of mortality emerged as a recurrent priority for clinicians [47, 55]. Caughey et al found that clinicians prioritised mortality in younger (less than 65 years) patients with

For peer review only - http://bmjopen.bmj.com/site/about/guidelines.xhtml

multimorbidity rather than older patients with multimorbidity, as they felt they could be more "aggressive" in their treatment [47]. Reducing the risk of mortality also emerged as a priority for patients across a number of studies [37, 38, 43, 50, 51] [42]. Some patients found the asymptomatic nature of hypertension to be concerning; hence, the consequences of hypertension could be unpredictable, compared to some other chronic illnesses where symptoms can give warning of onset and severity (Table 4) [38, 43].

iii. Symptom control

The symptomatic burden of a condition contributed to its "meaning as consequence" for patients [50]. Symptoms were cited as being a cause of functional limitation [38, 49], and in some cases their "unpredictability" could cause significant disruption to patients' daily lives [49]. Symptom control was reported to be a priority by some clinicians [37][47]. However, clinicians in one study considered symptom control to be less important, particularly when there was no risk of mortality [55]. In these cases, clinicians seemed to be aware that patients may still be prioritising symptom control highly, even if the clinicians did not (Table 4).

iv. Treatment burden

Factors related to the treatment burden of an illness appeared to adversely impact prioritisation for patients, leading to *de-prioritisation* of certain medications and treatments [38, 41, 43, 48]. Elliot et al. reported that cost and distressing side effects, were factors which led patients to stop taking a medication [43]. Similarly, Fried et al. found that patients reported unpleasant side effects to be a "competing outcome", which negatively influenced their decision regarding continuing a medication [41]. However, difficulty with achieving control over the management of an illness, as well as requirement for high levels of engagement with self-management, emerged as factors that contributed to the prioritisation of an illness by some patients (Table 4) [49].

	Concept	Examples from included studies
Factors influencing prioritisation	Functional ability	"I mean, because I have to be mobile, I am living on my own, no one is going to take care of me, I have got to look after myself.." Cheraghi-Sohi et al [50]
	Mortality	"Well I really do worry the most about the high blood pressure. 'Cause see you know you got arthritis and you can tell when it's coming on. But you can't hardly tell about high blood pressure. It can just hit you like that [snaps fingers] ….'" Lindsay et al [49]
	Symptom control	"I would not want to live with pain. I won't allow that to happen"- Naik et al [42]
	Disparity in prioritisation of symptom control	".. I talk [to her] for a quarter of an hour about this and that every time after which she replies, "but my vertigo," and I answer every time, well, unfortunately there is nothing I can do about it, we have already tried and done everything. But it is probably the first diagnosis she will mention: "What are you suffering from?". "Vertigo". For me, this would be somewhere all the way at the bottom." – Hansen et al [55]
	Treatment burden	"It's the knee that's the most concerning because everything else is controlled by tablets. The knee is a problem because if I have one little slip I'm in plaster again for 6 weeks." Lindsay et al [49]

Table 4- Examples from included studies for key concepts relating to factors influencing prioritisation

For peer review only - http://bmjopen.bmj.com/site/about/guidelines.xhtml

DISCUSSION

Prioritisation as a concept is broad, context-dependent and difficult to confine into a single definitive definition. As a result, determining what can be interpreted as a health outcome or treatment priority as part of our study selection in this review, was inherently difficult. We excluded some studies that investigated the preferences of patients with multimorbidity or clinicians, in contexts that we judged to be different to the aim of this review. These included patient preferences for healthcare delivery [68][69], levels of engagement with self-management practices [70][71] and clinicians' experiences of the management of patients with multimorbidity [18][72][73]. Whilst these studies represent very important areas of research, they were not within the scope of our aim in this review i.e. identifying studies that report the health outcome and treatment priorities of patients with multimorbidity or those of clinicians in relation to patients with multimorbidity. A discussion from our synthesis of findings of the included studies in this review is presented below.

Health outcome and treatment priorities

From our findings, patients' prioritisation appeared to be driven by weighing up the empirical compared to the hypothetical impact of a disease, whereby the empirical impact of a disease, which included its impact on function, symptomatic and treatment burden, was the most dominant driver of prioritisation. This is consistent with the findings of previous literature showing patients with rheumatoid arthritis who had reported experiencing higher levels of pain, were more likely to report pain as a priority [74].

Amongst empirical factors, preserving functionality emerged as most highly prioritised by patients amongst the quantitative studies that took a health outcome approach[39, 40, 46], whilst "function" was a domain that was prioritised highly by both patients and clinicians in the studies where prioritisation of various health *problems* were investigated [52-54]. From our qualitative findings, functional ability formed a key part of the preservation of various aspects of the patients' independence and their quality of life, as well as their ability to self-manage. Existing evidence shows that the prevalence of multimorbidity is highest in those aged over 65 years [75], and the population for the majority of the included studies were older adults with multimorbidity. This could provide an explanation for why preserving functionality was highly prioritised.

Prioritisation was not a static process and was subject to change, based on factors such as illness exacerbations, life events, whether there was a need for a treatment decision to be made, and whether the priority related to retrospective or prospective healthcare [49, 51]. When considering the hypothetical impact of an illness, perceptions of future risk came into play, and in particular, the risk of mortality [43]. This was particularly evident in relation to cardiovascular disease, where patients appeared to perceive the risk of mortality to be high [38].

Risk of mortality was a dominant driver for prioritisation amongst clinicians. This was shown in our quantitative synthesis, where amongst studies assessing disease-specific priorities, conditions with a higher risk of mortality, such as cardiovascular disease and diabetes, recurrently emerged as being highly prioritised by clinicians [36, 44, 56] and differentiated by age [47]. This age-based consideration could explain why clinicians prioritised "quality of life for the patient" as higher, albeit marginally, than "life expectancy of the patient" in their clinical decision-making for de-prescribing for elderly, patients with multimorbidity [57].

Smith et al previously developed a "Core Outcome Set" [76] in which a Delphi consensus panel formed of 26 international health experts, identified and prioritised a set of outcomes tailored for

For peer review only - http://bmjopen.bmj.com/site/about/guidelines.xhtml

application to research studies targeting patients with multimorbidity. Mortality, mental health outcomes and quality of life featured most highly in their list of prioritised outcomes, which also emerged in this review. However, we found that relatively few studies reported the prioritisation of mental health outcomes, with the exception of the studies that took a *problem-based* approach to prioritisation, where problems with regard to "Mood" were prioritised highly by both patients and clinicians [52-54].

Our findings show a varying degree of agreement between the priorities of patients with multimorbidity and clinicians. Previous studies carried out in the context of diabetes[77], and psoriasis[78] have found a low level of agreement on health outcome and treatment priorities between patients and clinicians, which correlates with the findings of some studies included in this review [52-54], but not others [44]. The nature of the patients' illnesses emerged as a factor for concordance or discordance of priorities with their clinicians [37]. Patients and clinicians were in agreement in situations where patients were currently experiencing an exacerbation of a particular condition, or had a "stable" state of health. However, in patients who suffered from illnesses with more complex courses, discordance of priorities tended to occur between patients and clinicians [37].

In recent times, the traditional *paternalistic* model for the doctor-patient relationship has given way to an *egalitarian* model [79], where doctors and patients each play an equitable role in a shared-decision making process, which places the patient at its core and thus achieving greater *patient-centred* care [80][79]. A shared agreement between patients and doctors on treatment priorities have been highlighted to play an important part in achieving patient-centred care and creating a *therapeutic alliance*, the benefits of which can include improved treatment adherence [79, 80]. Indeed, Jowsey et al found that agreement between patients and clinicians in the formulation of care plans promoted adherence to these plans, whereas a lack of agreement led to disengagement with care plans by patients [81].

Strengths and limitations

To our knowledge, this is the first systematic review to assimilate and compare the findings of existing literature on the health outcome and treatment priorities of both patients and clinicians for patients living with multi-morbidities. In this review, we have been able to add a novel line of argument to the ongoing discussion on this subject. By incorporating papers using both qualitative and quantitative methodologies, we have been able to explore patterns and relationships in the findings of a wide range of studies, thus creating a comprehensive and well-rounded systematic review.

There are noteworthy limitations. We did not include the term "comorbidity", in our search terms, and whilst "comorbidity" is distinctive from multimorbidity, there is also some conceptual overlap between the two terms. We felt that including "comorbidity" in our search strategy would identify studies focusing on a specific condition rather than multimorbidity.

A number of the quantitative studies did not use pre-validated tools to ascertain priorities [36, 54, 56-58], leading to a risk of measurement bias, which could limit the generalisability of findings in this review. All of the included studies were conducted in developed, western countries, which limits the global generalisability of our findings, as the priorities of patients with multimorbidity and of clinicians in developing and/or eastern countries may differ to the findings of this review.

We also detected a large variation in the tools used to ascertain priorities, which meant that carrying out a meta-analysis to synthesise the findings of the quantitative studies was not possible. Yet, we

For peer review only - http://bmjopen.bmj.com/site/about/guidelines.xhtml

have tried to mitigate the lack of meta-analysis by using a well-described and well-established method of narrative synthesis [33], in order to maintain rigour and transparency.

Another limitation is that in our inclusion criteria we chose to also include studies which did not explicitly specify a definition of multimorbidity as “two or more chronic conditions” in their inclusion criteria but had a sample of participants that were reflective of multimorbidity (i.e. with a minimum of two chronic conditions which could be identified from participant demographic data). We chose to do this as in the absence of a universally accepted and uniform definition of multimorbidity, we sought to base our judgement on the inclusivity of each paper on its value in answering our review question. This, along with the previously discussed difficulty in defining prioritisation, may have introduced a degree of subjective interpretation in the process of study selection, despite our attempt to mitigate this by incorporating independent review of the results of our literature searching by two reviewers in duplicate.

Recommendations for the future

We recommend that future guidelines developed for clinicians in the management of multimorbidity highlight the need to elicit and consider both short term and long term priorities for their patients’, as our review has shown that patients’ priorities for their current illness experiences and future risks posed by illnesses, may differ. In accordance with current NICE guidance, we also reiterate the need to review these priorities continually, and particularly when exacerbations, changes to illness course or treatment regimens, or other wider socially-contextualised changes occur in their patients’ lives.

There was a large variation in how priorities were ascertained, and in the tools used to ascertain priorities. The relative lack of standardised and validated tools for use to ascertain patient priorities in everyday clinical practice has also been described in previous literature [82]. We highlight a need for the development of a standardised and validated tool that is acceptable to both patients and clinicians, and can be used to ascertain patient-priorities in the multiple dimensions described in this review. Such a tool would a valuable aid to treatment decision-making, advance care planning and achieving patient-centeredness for patients living with multimorbidity.

Conclusion

The findings from this review show the priorities of patients and clinicians can have varying degrees of concordance, being mostly low [52, 54], in alignment with previous findings in single disease contexts [77, 78]. We have found that the mechanisms of prioritisation can also differ between our two groups, in that patients are driven by illness experiences, whereas clinicians may be focused on managing longer term risks. Understanding these differences can help clinicians to better recognise situations where the patients’ priorities may be different to theirs and elicit the most important priorities for their patients.

For peer review only - http://bmjopen.bmj.com/site/about/guidelines.xhtml

REFERENCES

1 van den Akker M, Buntinx F, Knottnerus JA. Comorbidity or multimorbidity: what's in a name? A review of literature, *The European Journal of General Practice* 1996;2:65-70.

2 Garin N, Koyanagi A, Chatterji S, et al. Global multimorbidity patterns: a cross-sectional, population-based, multi-country study, *Journals of Gerontology Series A: Biomedical Sciences and Medical Sciences* 2015;71:205-14.

3 Rijken M, Struckmann V, Dyakova M, et al. ICARE4EU: Improving care for people with multiple chronic conditions in Europe. 2013.

4 Roberts KC, Rao DP, Bennett TL, et al. Prevalence and patterns of chronic disease multimorbidity and associated determinants in Canada, *Health Promot Chronic Dis Prev Can* 2015;35:87-94.

5 Rocca WA, Boyd CM, Grossardt BR, et al. Prevalence of multimorbidity in a geographically defined American population: patterns by age, sex, and race/ethnicity. 2014;89:1336-49.

6 Arokiasamy P, Uttamacharya U, Jain K, et al. The impact of multimorbidity on adult physical and mental health in low-and middle-income countries: what does the study on global ageing and adult health (SAGE) reveal? *BMC medicine* 2015;13:178.

7 Afshar S, Roderick PJ, Kowal P, et al. Multimorbidity and the inequalities of global ageing: a cross-sectional study of 28 countries using the World Health Surveys, *BMC Public Health* 2015;15:776.

8 Tinetti ME, Bogardus Jr ST, Agostini JV. Potential pitfalls of disease-specific guidelines for patients with multiple conditions, *N Engl J Med* 2004;351:2870-4.

9 Calderon-Larranaga A, Poblador-Plou B, Gonzalez-Rubio F, et al. Multimorbidity, polypharmacy, referrals, and adverse drug events: are we doing things well? *Br J Gen Pract* 2012;62:e821-6.

10 Fortin M, Bravo G, Hudon C, et al. Relationship between multimorbidity and health-related quality of life of patients in primary care, *Quality of Life Research* 2006;15:83-91.

11 Marengoni A, Angleman S, Melis R, et al. Aging with multimorbidity: a systematic review of the literature, *Ageing research reviews* 2011;10:430-9.

12 Fortin M, Bravo G, Hudon C, et al. Psychological distress and multimorbidity in primary care, *Ann Fam Med* 2006;4:417-22.

13 Academy of Medical Sciences. Multimorbidity: a priority for global health research, 2018.

14 Muth C, van den Akker M, Blom JW, et al. The Ariadne principles: how to handle multimorbidity in primary care consultations. *BMC Medicine* 2014;12:223.

15 Azad NA, Mielniczuk L. A call for collaboration: improving cardiogeriatric care, *Can J Cardiol* 2016;32:1041-4.

16 Roland M, Paddison C. Better management of patients with multimorbidity, *BMJ: British Medical Journal (Online)* 2013;346.

For peer review only - http://bmjopen.bmj.com/site/about/guidelines.xhtml

17 Bierman AS, Tinetti ME. Precision medicine to precision care: managing multimorbidity, *Lancet* 2016;388:2721-3.

18 Mc Namara KP, Breken BD, Alzubaidi HT, et al. Health professional perspectives on the management of multimorbidity and polypharmacy for older patients in Australia, *Age & Ageing* 2017;46:291-9.

19 Sinnott C, Mc Hugh S, Browne J, et al. GPs' perspectives on the management of patients with multimorbidity: systematic review and synthesis of qualitative research, *BMJ Open* 2013;3:e003610,2013-003610.

20 Rothwell PM, McDowell Z, Wong CK, et al. Doctors and patients don't agree: cross sectional study of patients' and doctors' perceptions and assessments of disability in multiple sclerosis, *BMJ* 1997;314:1580-3.

21 Montgomery AA, Fahey T. How do patients' treatment preferences compare with those of clinicians? *Qual Health Care* 2001;10 Suppl 1:i39-43.

22 Thomson S, Doody G. Parallel paths? Patient and doctor priorities in psychiatric outpatient consultations, *Journal of Mental Health* 2010;19:461-9.

23 Lee CN, Hultman CS, Sepucha K. Do patients and providers agree about the most important facts and goals for breast reconstruction decisions? *Ann Plast Surg* 2010;64:563-6.

24 Volandes AE, Paasche-Orlow MK, Barry MJ, et al. Video decision support tool for advance care planning in dementia: randomised controlled trial, *BMJ* 2009;338:b2159.

25 Pager CK, McCluskey PJ. Surgeons' perceptions of their patients' priorities, *Journal of Cataract & Refractive Surgery* 2004;30:591-7.

26 Higgins JP, Green S. Cochrane handbook for systematic reviews of interventions: John Wiley & Sons 2011.

27 Moher D, Shamseer L, Clarke M, et al. Preferred reporting items for systematic review and meta-analysis protocols (PRISMA-P) 2015 statement, *Systematic reviews* 2015;4:1.

28 Moher D, Liberati A, Tetzlaff J, et al. Preferred reporting items for systematic reviews and meta-analyses: the PRISMA statement, *Ann Intern Med* 2009;151:264-9.

29 Downes MJ, Brennan ML, Williams HC, et al. Development of a critical appraisal tool to assess the quality of cross-sectional studies (AXIS), *BMJ Open* 2016;6:e011458,2016-011458.

30 Stang A. Critical evaluation of the Newcastle-Ottawa scale for the assessment of the quality of nonrandomized studies in meta-analyses, *Eur J Epidemiol* 2010;25:603-5.

31 Higgins JP, Altman DG, Gotzsche PC, et al. The Cochrane Collaboration's tool for assessing risk of bias in randomised trials, *BMJ* 2011;343:d5928.

32 Critical Appraisal Skills Programme UK. CASP qualitative research checklist. *CASP checklists* 13/03/2017.

For peer review only

33 Popay J, Roberts H, Sowden A, et al. Guidance on the conduct of narrative synthesis in systematic reviews, *A product from the ESRC methods programme Version* 2006;1:b92.

34 Noblit GW, Hare RD. Meta-ethnography: Synthesizing qualitative studies: sage 1988.

35 Britten N, Campbell R, Pope C, et al. Using meta ethnography to synthesise qualitative research: a worked example, *J Health Serv Res Policy* 2002;7:209-15.

36 Moore A., Patterson C., Nair K., et al. Minding the gap: Prioritization of care issues among nurse practitioners, family physicians and geriatricians when caring for the elderly. *Journal of interprofessional care* 2015;29:401-3.

37 Kuluski K, Gill A, Naganathan G, et al. A qualitative descriptive study on the alignment of care goals between older persons with multi-morbidities, their family physicians and informal caregivers. *BMC Family Practice* 2013;14:133.

38 Schoenberg NE, Leach C, Edwards W. "It's a toss up between my hearing, my heart, and my hip": prioritizing and accommodating multiple morbidities by vulnerable older adults, *J Health Care Poor Underserved* 2009;20:134-51.

39 Fried TR, Tinetti ME, Iannone L, et al. Health outcome prioritization as a tool for decision making among older persons with multiple chronic conditions, *Arch Intern Med* 2011;171:1856-8.

40 Fried TR, Tinetti M, Agostini J, et al. Health outcome prioritization to elicit preferences of older persons with multiple health conditions, *Patient Educ Couns* 2011;83:278-82.

41 Fried TR, McGraw S, Agostini JV, et al. Views of older persons with multiple morbidities on competing outcomes and clinical decision-making, *J Am Geriatr Soc* 2008;56:1839-44.

42 Naik A.D., Martin L.A., Moye J., et al. Health Values and Treatment Goals of Older, Multimorbid Adults Facing Life-Threatening Illness. *J Am Geriatr Soc* 2016;64:625-31.

43 Elliott RA, Ross-Degnan D, Adams AS, et al. Strategies for coping in a complex world: adherence behavior among older adults with chronic illness, *Journal of General Internal Medicine* 2007;22:805-10.

44 Zulman D.M., Kerr E.A., Hofer T.P., et al. Patient-provider concordance in the prioritization of health conditions among hypertensive diabetes patients. *Journal of General Internal Medicine* 2010;25:408-14.

45 Summeren JJ, Haaijer-Ruskamp FM, Schuling J. Eliciting preferences of multimorbid elderly adults in family practice using an outcome prioritization tool, *J Am Geriatr Soc* 2016;64.

46 van Summeren JJ, Schuling J, Haaijer-Ruskamp FM, et al. Outcome prioritisation tool for medication review in older patients with multimorbidity: a pilot study in general practice, *Br J Gen Pract* 2017;67:e501-6.

47 Caughey G.E., Tait K., Vitry A.I., et al. Influence of medication risks and benefits on treatment preferences in older patients with multimorbidity. *Patient Preference and Adherence* 2017;11:131-40.

For peer review only

48 Turner J.P., Edwards S., Stanners M., et al. What factors are important for deprescribing in Australian long-term care facilities? Perspectives of residents and health professionals. *BMJ Open* 2016;6:Arte Number: e009781. ate of Pubaton: 2016.

49 LINDSAY S. PRIORITIZING ILLNESS: LESSONS IN SELFa MANAGING MULTIPLE CHRONIC DISEASES, *CANADIAN JOURNAL OF SOCIOLOGY/CAHIERS CANADIENS DE SOCIOLOGIE* 2009;34:983.

50 Cheraghi-Sohi S, Morden A, Bower P, et al. Exploring patient priorities among long-term conditions in multimorbidity: A qualitative secondary analysis. *SAGE Open Medicine* 2013;1:2050312113503955.

51 Morris R.L., Sanders C., Kennedy A.P., et al. Shifting priorities in multimorbidity: A longitudinal qualitative study of patient's prioritization of multiple conditions. *Chronic Illness* 2011;7:147-61.

52 Junius-Walker U, Wrede J, Voigt I, et al. Impact of a priority-setting consultation on doctor-patient agreement after a geriatric assessment: cluster randomised controlled trial in German general practices. *Quality in primary care* 2012;20.

53 Junius-Walker U, Stolberg D, Steinke P, et al. Health and treatment priorities of older patients and their general practitioners: a cross-sectional study. *Quality in primary care* 2011;19.

54 Voigt I, Wrede J, Diederichs-Egidi H, et al. Priority setting in general practice: health priorities of older patients differ from treatment priorities of their physicians, *Croat Med J* 2010;51:483-92.

55 Hansen H., Pohontsch N., van den Bussche H., et al. Reasons for disagreement regarding illnesses between older patients with multimorbidity and their GPs - a qualitative study. *BMC family practice* 2015;16:68.

56 Herzig L, Mueller Y, Haller DM, et al. Family practitioners' top medical priorities when managing patients with multimorbidity: a cross-sectional study, *BJGP open* 2019;3:bjgpopen18X101622.

57 Mantelli S, Jungo KT, Rozsnyai Z, et al. How general practitioners would deprescribe in frail oldest-old with polypharmacy—the LESS study, *BMC family practice* 2018;19:169.

58 Déruaz-Luyet A, N'Goran AA, Pasquier J, et al. Multimorbidity: can general practitioners identify the health conditions most important to their patients? Results from a national cross-sectional study in Switzerland, *BMC family practice* 2018;19:66.

59 Tinetti ME, McAvay GJ, Fried TR, et al. Health outcome priorities among competing cardiovascular, fall injury, and medication-related symptom outcomes, *J Am Geriatr Soc* 2008;56:1409-16.

60 DeruazLuyet A., Alexandra N'Goran A., Tandjung R., et al. Multimorbidity in primary care: Protocol of a national cross-sectional study in Switzerland. *BMJ Open* 2015;5:Arte Number: e009165. ate of Pubaton: 2015.

61 Martin LA, Moye J, Street Jr RL, et al. Reconceptualizing cancer survivorship through veterans' lived experiences, *J Psychosoc Oncol* 2014;32:289-309.

62 Lindsay S, Bellaby P, Smith S, et al. Enabling healthy choices: is ICT the highway to health improvement? *Health:* 2008;12:313-31.

For peer review only - http://bmjopen.bmj.com/site/about/guidelines.xhtml

63 Hurley MV, Walsh N, Bhavnani V, et al. Health beliefs before and after participation on an exercised-based rehabilitation programme for chronic knee pain: doing is believing, *BMC Musculoskeletal Disorders* 2010;11:31.

64 Bower P, Harkness E, Macdonald W, et al. Illness representations in patients with multimorbid long-term conditions: Qualitative study, *Psychol Health* 2012;27:1211-26.

65 Grime J, Richardson JC, Ong BN. Perceptions of joint pain and feeling well in older people who reported being healthy: a qualitative study, *Br J Gen Pract* 2010;60:597-603.

66 Nio Ong B, Jinks C, Morden A. The hard work of self-management: Living with chronic knee pain, *International journal of qualitative studies on health and well-being* 2011;6:7035.

67 Hansen H., Schafer I., Schon G., et al. Agreement between self-reported and general practitioner-reported chronic conditions among multimorbid patients in primary care - results of the MultiCare Cohort Study. *BMC family practice* 2014;15:39.

68 Noel P.H., Frueh B.C., Larme A.C., et al. Collaborative care needs and preferences of primary care patients with multimorbidity. *Health Expectations* 2005;8:54-63.

69 Lechner S., Herzog W., Boehlen F., et al. Control preferences in treatment decisions among older adults - Results of a large population-based study. *J Psychosom Res* 2016;86:28-33.

70 Noel PH, Parchman ML, Williams JWJ, et al. The challenges of multimorbidity from the patient perspective. *Journal of General Internal Medicine* 2007;22:419-24.

71 Coventry PA, Fisher L, Kenning C, et al. Capacity, responsibility, and motivation: a critical qualitative evaluation of patient and practitioner views about barriers to self-management in people with multimorbidity, *BMC health services research* 2014;14:536.

72 Sinnott C., Mc Hugh S., Boyce M.B., et al. What to give the patient who has everything? A qualitative study of prescribing for multimorbidity in primary care. *British Journal of General Practice* 2015;65:e184-91.

73 Luijks HD, Loeffen MJW, Lagro-Janssen AL, et al. GPs' considerations in multimorbidity management: a qualitative study. *British Journal of General Practice* 2012;62:e503-10.

74 Heiberg T, Kvien TK. Preferences for improved health examined in 1,024 patients with rheumatoid arthritis: pain has highest priority, *Arthritis Care & Research* 2002;47:391-7.

75 Barnett K, Mercer SW, Norbury M, et al. Epidemiology of multimorbidity and implications for health care, research, and medical education: a cross-sectional study, *The Lancet* 2012;380:37-43.

76 Smith SM, Wallace E, Salisbury C, et al. A Core Outcome Set for Multimorbidity Research (COSmm), *Ann Fam Med* 2018;16:132-8.

77 Heisler M, Vijan S, Anderson RM, et al. When do patients and their physicians agree on diabetes treatment goals and strategies, and what difference does it make? *Journal of general internal medicine* 2003;18:893-902.

For peer review only

78 Okubo Y, Tsuruta D, Tang A, et al. Analysis of treatment goal alignment between Japanese psoriasis patients and their paired treating physicians, *Journal of the European Academy of Dermatology and Venereology* 2018;32:606-14.

79 Mead N, Bower P. Patient-centredness: a conceptual framework and review of the empirical literature, *Soc Sci Med* 2000;51:1087-110.

80 Kaba R, Sooriakumaran P. The evolution of the doctor-patient relationship, *International Journal of Surgery* 2007;5:57-65.

81 Jowsey T, Dennis S, Yen L, et al. Time to manage: patient strategies for coping with an absence of care coordination and continuity, *Sociol Health Illn* 2016;38:854-73.

82 Mangin D., Stephen G., Bismah V., et al. Making patient values visible in healthcare: A systematic review of tools to assess patient treatment priorities and preferences in the context of multimorbidity. *BMJ Open* 2016;6:Arte Number: e010903. ate of Pubaton: 01 Jun 2016.

Figure legends:

Figure 1: Flow diagram to illustrate process from literature searching to selection of studies for inclusion [28]

For peer review only

For peer review only - http://bmjopen.bmj.com/site/about/guidelines.xhtml

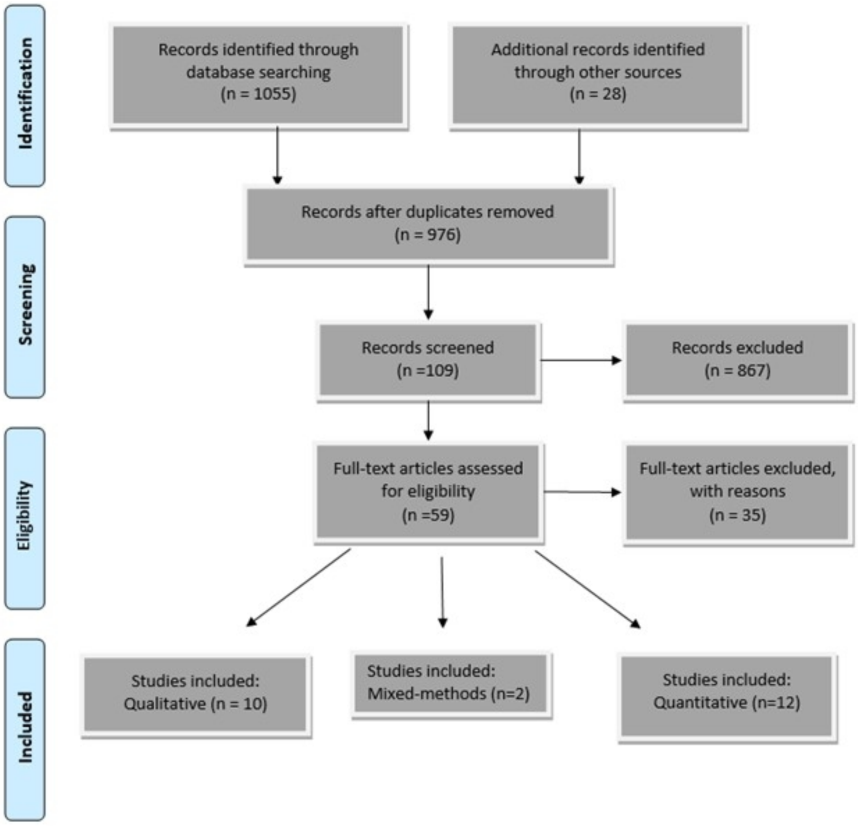

Figure 1 Flow diagram to illustrate process from literature searching to selection of studies for inclusion [28]

121x111mm (150 x 150 DPI)

1. Patient*.mp.
2. Patients/
3. 1 or 2
4. Priorit*.mp. [mp=title, abstract, original title, name of substance word, subject heading word, keyword heading word, protocol supplementary concept word, rare disease supplementary concept word, unique identifier, synonyms]
5. Choice*.mp.
6. Preference*.mp.
7. Aim*.mp.
8. Goal*.mp.
9. 4 or 5 or 6 or 7 or 8
10. Doctor*.mp.
11. Physicians/
12. Clinician*.mp.
13. Primary Health Care/ or Physicians, Family/ or Family Practice/ or General Practitioners/
14. General practitioner*.mp.
15. 10 or 11 or 12 or 13 or 14
16. Multimorbidit*.mp. [mp=title, abstract, original title, name of substance word, subject heading word, keyword heading word, protocol supplementary concept word, rare disease supplementary concept word, unique identifier, synonyms]
17. Multi-morbidit*.mp. [mp=title, abstract, original title, name of substance word, subject heading word, keyword heading word, protocol supplementary concept word, rare disease supplementary concept word, unique identifier, synonyms]
18. Multiple morbidit*.mp. [mp=title, abstract, original title, name of substance word, subject heading word, keyword heading word, protocol supplementary concept word, rare disease supplementary concept word, unique identifier, synonyms]
19. 16 or 17 or 18
20. 3 and 9 and 15 and 19
21. Multi morbid*.mp. [mp=title, abstract, original title, name of substance word, subject heading word, keyword heading word, protocol supplementary concept word, rare disease supplementary concept word, unique identifier, synonyms]
22. 16 or 17 or 18 or 21
23. 3 and 9 and 15 and 22

Appendix 1

For peer review only

CASP checklist for qualitative research summary table

	**Kuluski et al** [1]	**Schoenberg et al** [2]	**Cheraghi-Sohi et al** [3]	**Naik et al** [4]	**Lindsay et al** [5]	**Hansen et al** [6]	**Morris et al** [7]	**Elliott et al** [8]	**Fried et al** [9]	**Turner et al** [10]	**Van Summeren et al** [11]	**Caughey et al** [12]
Was there a clear statement of the aims of the research?	YES	YES	YES	YES	YES	YES	YES	YES	YES	YES	YES	YES
Is a qualitative methodology appropriate?	YES	YES	YES	YES	YES	YES	YES	YES	YES	NO- Quantitative or mixed methods methodology would have been more appropriate as the aim was to rank factors, although data collected using a qualitative technique, it lacks richness and appears to be presented in a quantitative manner	YES	YES
Was the research design appropriate to the aims of the research?	YES	YES	YES	YES	YES	YES	YES	YES	YES	YES	YES	YES
Was the recruitment strategy	YES	YES	YES	YES	YES	YES	NO- no explanation given as to	YES	YES	YES	YES	YES

For peer review only

appropriate to the aims of the research							why the specific conditions were chosen (COPD, IBS etc)					
Were the data collected in a way that addressed the research issue?	YES	YES	YES	YES	YES	YES	YES	YES	YES	YES	YES	YES
Has the relationship between researcher and participants been adequately considered?	YES	YES	YES	NO- no information given on background of main researcher and no consideration given to possibility of researcher bias at any point.	NO- There is no background information given on the researcher (sole in this case) and there has been no evidence of any consideration of researcher bias at any point during the study.	NO- there has been no evidence of any consideration of researcher bias at any point during the study	NO- no information given on background of main researcher and no consideration given to possibility of researcher bias at any point. However there was some evidence of reflexivity during the data collection process when emerging areas of interest that could be incorporated into future interviews	NO- background of RAE who conducted interviews and main aspect of analysis not specified and no consideration has been given to any possibility of researcher bias	NO- No explanation given of the professional background of the researchers or the moderator for the focus groups, and there has been no evidence of any consideration of researcher bias at any point during the study.	NO- no mention of the background of the researchers or how this may have influenced the results	NO- role of second interviewer carrying out the in-depth interviews not mentioned, and there has been no consideration given to the possibility of bias from the interviewers. One of the interviewers was a FP, which could have led to bias with the interviewees responses.	NO- there has been no consideration given to the role of the researcher and the potential for researcher bias at any point.

For peer review only

							were considered.					
Have ethical issues been taken into consideration?	YES	YES	YES- in the original studies, however further ethical issues regarding secondary qualitative analysis were not taken into account.	YES	YES	YES	YES	YES	YES	YES	YES	YES
Was the data analysis sufficiently rigorous?	YES	YES	YES	YES	NO- superficial description of analytic process and no information given on how many researchers analysed the transcripts- assumed one as there is only one author- risk of bias not taken into account for the analytic process	YES	YES	YES	YES	NO- the data analysis process is very ambiguous and the qualitative analysis has not been described in sufficient depth.	YES- clear description of the analytic process with two researchers independently analysing the data for rigour. However no description of the interpretation phase from the data.	NO- there is only a superficial description of the data analysis process, and there is very little detail given on how the themes were derived from the data. There is no presentation at all of quotes from the data to support the authors interpretation of the data.
Is there a clear	YES	YES	YES	YES	YES	YES	YES	YES	YES	YES	YES- however the qualitative	YES- however no quotes given

For peer review only

statement of findings?											data from the patient interviews has only been summarised- no direct quotes given	to support findings
How valuable is the research?	Valuable	Valuable	Valuable	Valuable	Valuable	Valuable	Valuable	Valuable	Valuable	Valuable	Valuable	Valuable

For peer review only

Newcastle-ottawa scale for cohort and observational studies summary table								
	Representativeness of the exposed cohort	Selection of the non-exposed cohort	Ascertainment of exposure	Demonstration that outcome of interest was not present at start of study	Comparability of cohorts on the basis of the design or analysis controlled for confounders	Assessment of outcome	Was follow-up long enough for outcomes to occur	Adequacy of follow-up of cohorts
Zulman et al [13]	Somewhat representative (one star) *	Drawn from the same community as the exposed cohort (one star) *	Secure record (one star) *	N/A	The study controls for age, sex and marital status (one star)*	Self-report	N/A	No statement

For peer review only

Axis tool for cross-sectional studies summary table

Introduction		Junius-Walker et al [14]	Fried et al[15]	Fried et al [16]	Moore et al [17]	Van Summeren et al [18]	Voigt et al [19]	Van Summeren et al [11]	Caughey et al [20]	Mantelli et al [21]	Deruaz-Luyet et al [22]	Herzig et al [23]
1	Were the aims/objectives of the study clear?	Yes	Yes	Yes	Yes	Yes	Yes	Yes	Yes	Yes	Yes	Yes
Methods												
2	Was the study design appropriate for the stated aim(s)?	Yes	Yes	Yes	Yes	Yes	Yes	Yes	Yes	Yes	Yes	Yes
3	Was the sample size justified?	No- convenience sampling used, small sample size, however no explanation for sample size given	No- no justification for sample size given, convenience sampling used	No- recruitment strategy described clearly but no justification for sample size given	Yes	No	No- sampling strategy described well but no justification for sample size given	No- purposive sampling used, however no justification for sample size given	No- no justification for sample size given	No- convenience sampling used and no justification for sample size given	Yes- in the parent study [24]	Yes- in the parent study [24]
4	Was the target/reference population clearly defined? (Is it clear who the research was about?)	Yes	Yes	Yes	Yes	Yes	Yes	Yes	Yes	Yes	Yes	Yes
5	Was the sample frame taken from an appropriate population base so that it closely represented the target/reference population under investigation?	Yes	Yes- However assumption made that participants will have multiple chronic conditions	Yes	Yes	Yes	Yes	Yes	Yes	Yes- although only GP's who had previously taken part in other case-vignette studies were invited, leading to possibility of selection bias	Yes	Yes

For peer review only

6	Was the selection process likely to select subjects/participants that were representative of the target/reference population under investigation?	Yes	Yes- as above	Yes	Yes	Yes	Yes	Yes	Yes	Yes- as above	Yes	Yes
7	Were measures undertaken to address and categorise non-responders?	Yes	Don't know- not reported	Yes	No	No	No	Yes- Purposive sampling used with efforts made to address gaps in participant types	Don't know- not reported	Don't know- not reported	Yes in the parent study [25].<br><br>Characteristics of participants who were not included due to missing data, were described in this study	Yes in the parent study [25]
8	Were the risk factor and outcome variables measured appropriate to the aims of the study?	Yes	Yes	Yes	Yes	Yes	Yes	Yes	Yes	Yes	Yes	Yes
9	Were the risk factor and outcome variables measured correctly using instruments/measurements that had been trialled, piloted or published previously?	Yes	Yes- piloted in a previous study	No- Tested in this study as it was a feasibility study	No- Pre-tested in this study but only using 2 FP's and 1 NP	Yes	No- STEP assessment previously published however no testing done of measure used to collect importance ratings	Yes	Yes	Yes- the instruments used were piloted within this study using 5 GP's as participants, but had not been published previously	No- instruments designed through "internal consensus discussions".	No

10	Is it clear what was used to determined statistical significance and/or precision estimates? (e.g. p-values, confidence intervals)	Yes	N/A	Yes	Yes	N/A	Yes	N/A	N/A	Yes	Yes	Yes
11	Were the methods (including statistical methods) sufficiently described to enable them to be repeated?	Yes	Yes	Yes	Yes	Yes	Yes	Yes	Yes	Yes	Yes	Yes
Results												
12	Were the basic data adequately described?	Yes	Yes	Yes	Yes	No- No reporting of prioritisation of patients for whom no medication changes were proposed	Yes	Yes	Yes	Yes	Yes	Yes
13	Does the response rate raise concerns about non-response bias?	No	Don't know- response rate not reported	No	No	No	Don't know- response rate not reported	No	Don't know- response rate not reported	No	No	No
14	If appropriate, was information about non-responders described?	Yes	No	Yes	No	Yes	No	Yes	No	No	Yes in the parent study[25]<br><br>Characteristics of participants who were not included	Yes in the parent study[25]

For peer review only

											due to missing data, were described in this study	
15	Were the results internally consistent?	Yes	Yes	Yes	Yes	Yes	Yes	Yes	Yes	Yes	Yes	Yes
16	Were the results presented for all the analyses described in the methods?	Yes	Yes	Yes	Yes	No- No reporting of prioritisation of patients for whom no medication changes were proposed	Yes	Yes	Yes	Yes	Yes	Yes
Discussion												
17	Were the authors' discussions and conclusions justified by the results?	No- very small sample of GP's compared to patients therefore generalizable conclusions regarding concordance between doctors and patients cannot accurately be drawn from this study	Yes	Yes	Yes	Yes	Yes	Yes- Small sample size for quantitative aspect of study taken into account	No- very small sample size across patients and clinicians, meaning results are not generalizable	Yes	Yes	Yes

For peer review only

18	Were the limitations of the study discussed?	Yes	No	Yes	Yes	Yes	Yes	Yes	Yes	Yes	Yes	Yes
Other												
19	Were there any funding sources or conflicts of interest that may affect the authors' interpretation of the results?	No	No	No	No	No	No	No	No	No	No	No
20	Was ethical approval or consent of participants attained?	Yes	Yes	Yes	Yes	Yes	Yes	Yes	Yes	Yes	Yes	Yes

For peer review only

The Cochrane Collaboration's tool for assessing risk of bias in randomised controlled trials summary table		
Study	Junius-Walker et al [26]	
Bias	Authors' judgement	Support for judgement
Random sequence generation (selection bias)	Low risk	"Participating doctors were allocated 1:1 into the intervention and control group using random block sizes of four"
Allocation concealment (selection bias)	Unclear risk	No information given regarding any efforts to conceal the allocation sequence
Blinding of participants and researchers (performance bias)	Low risk	Participants were only informed of the procedures of their own trial arm.
Blinding of outcome assessment (detection bias)	Low risk	Participants were blinded to the pre-intervention importance ratings, when completing the final importance ratings.
Incomplete outcome data (attrition bias)	High risk	25 patients dropped out prior to baseline ratings and 5 further patients dropped out prior to final ratings, these patients were excluded from analysis, however intention to treat analysis cannot be carried out in this context due to the nature of the intervention
Selective reporting (reporting bias)	Low risk	Adequate reporting on all of the specified outcomes
Other bias	None detected	

For peer review only

References

1 Kuluski K, Gill A, Naganathan G, et al. A qualitative descriptive study on the alignment of care goals between older persons with multi-morbidities, their family physicians and informal caregivers. *BMC Family Practice* 2013;14:133.

2 Schoenberg NE, Leach C, Edwards W. "It's a toss up between my hearing, my heart, and my hip": prioritizing and accommodating multiple morbidities by vulnerable older adults, *J Health Care Poor Underserved* 2009;20:134-51.

3 Cheraghi-Sohi S, Morden A, Bower P, et al. Exploring patient priorities among long-term conditions in multimorbidity: A qualitative secondary analysis. *SAGE Open Medicine* 2013;1:2050312113503955.

4 Naik A.D., Martin L.A., Moye J., et al. Health Values and Treatment Goals of Older, Multimorbid Adults Facing Life-Threatening Illness. *J Am Geriatr Soc* 2016;64:625-31.

5 LINDSAY S. PRIORITIZING ILLNESS: LESSONS IN SELFa MANAGING MULTIPLE CHRONIC DISEASES, *CANADIAN JOURNAL OF SOCIOLOGY/CAHIERS CANADIENS DE SOCIOLOGIE* 2009;34:983.

6 Hansen H., Pohontsch N., van den Bussche H., et al. Reasons for disagreement regarding illnesses between older patients with multimorbidity and their GPs - a qualitative study. *BMC family practice* 2015;16:68.

7 Morris R.L., Sanders C., Kennedy A.P., et al. Shifting priorities in multimorbidity: A longitudinal qualitative study of patient's prioritization of multiple conditions. *Chronic Illness* 2011;7:147-61.

8 Elliott RA, Ross-Degnan D, Adams AS, et al. Strategies for coping in a complex world: adherence behavior among older adults with chronic illness, *Journal of General Internal Medicine* 2007;22:805-10.

9 Fried TR, McGraw S, Agostini JV, et al. Views of older persons with multiple morbidities on competing outcomes and clinical decision-making, *J Am Geriatr Soc* 2008;56:1839-44.

10 Turner J.P., Edwards S., Stanners M., et al. What factors are important for deprescribing in Australian long-term care facilities? Perspectives of residents and health professionals. *BMJ Open* 2016;6:Arte Number: e009781. ate of Pubaton: 2016.

11 Summeren JJ, Haaijer-Ruskamp FM, Schuling J. Eliciting preferences of multimorbid elderly adults in family practice using an outcome prioritization tool, *J Am Geriatr Soc* 2016;64.

For peer review only

12 Caughey G.E., Huynh E., Shakib S., et al. Influence of medication risks and benefits on patient and clinician preferences for treatment in multimorbidity: A discrete-choice experiment. 2017.

13 Zulman D.M., Kerr E.A., Hofer T.P., et al. Patient-provider concordance in the prioritization of health conditions among hypertensive diabetes patients. *Journal of General Internal Medicine* 2010;25:408-14.

14 Junius-Walker U, Stolberg D, Steinke P, et al. Health and treatment priorities of older patients and their general practitioners: a cross-sectional study. *Quality in primary care* 2011;19.

15 Fried TR, Tinetti ME, Iannone L, et al. Health outcome prioritization as a tool for decision making among older persons with multiple chronic conditions, *Arch Intern Med* 2011;171:1856-8.

16 Fried TR, Tinetti M, Agostini J, et al. Health outcome prioritization to elicit preferences of older persons with multiple health conditions, *Patient Educ Couns* 2011;83:278-82.

17 Moore A., Patterson C., Nair K., et al. Minding the gap: Prioritization of care issues among nurse practitioners, family physicians and geriatricians when caring for the elderly. *Journal of interprofessional care* 2015;29:401-3.

18 Van Summeren J.J.G.T., Schuling J., HaaijerRuskamp F.M., et al. Outcome prioritisation tool for medication review in older patients with multimorbidity: A pilot study in general practice. *British Journal of General Practice* 2017;67:e501-6.

19 Voigt I, Wrede J, Diederichs-Egidi H, et al. Priority setting in general practice: health priorities of older patients differ from treatment priorities of their physicians, *Croat Med J* 2010;51:483-92.

20 Caughey G.E., Tait K., Vitry A.I., et al. Influence of medication risks and benefits on treatment preferences in older patients with multimorbidity. *Patient Preference and Adherence* 2017;11:131-40.

21 Mantelli S, Jungo KT, Rozsnyai Z, et al. How general practitioners would deprescribe in frail oldest-old with polypharmacy—the LESS study, *BMC family practice* 2018;19:169.

22 Déruaz-Luyet A, N'Goran AA, Pasquier J, et al. Multimorbidity: can general practitioners identify the health conditions most important to their patients? Results from a national cross-sectional study in Switzerland, *BMC family practice* 2018;19:66.

For peer review only

23 Herzig L, Mueller Y, Haller DM, et al. Family practitioners' top medical priorities when managing patients with multimorbidity: a cross-sectional study, *BJGP open* 2019;3:bjgpopen18X101622.

24 DeruazLuyet A., Alexandra N'Goran A., Tandjung R., et al. Multimorbidity in primary care: Protocol of a national cross-sectional study in Switzerland. *BMJ Open* 2015;5:Arte Number: e009165. ate of Pubaton: 2015.

25 Deruaz-Luyet A, N'Goran AA, Senn N, et al. Multimorbidity and patterns of chronic conditions in a primary care population in Switzerland: a cross-sectional study, *BMJ Open* 2017;7:e013664,2016-013664.

26 Junius-Walker U, Wrede J, Voigt I, et al. Impact of a priority-setting consultation on doctor-patient agreement after a geriatric assessment: cluster randomised controlled trial in German general practices. *Quality in primary care* 2012;20.

For peer review only

Reporting checklist for systematic review and meta-analysis.

Based on the PRISMA guidelines.

		Reporting Item	Page Number
**Title**			
	#1	Identify the report as a systematic review, meta-analysis, or both.	1
**Abstract**			
Structured summary	#2	Provide a structured summary including, as applicable: background; objectives; data sources; study eligibility criteria, participants, and interventions; study appraisal and synthesis methods; results; limitations; conclusions and implications of key findings; systematic review registration number	2
**Introduction**			
Rationale	#3	Describe the rationale for the review in the context of what is already known.	3

For peer review only

Objectives	#4	Provide an explicit statement of questions being addressed with reference to participants, interventions, comparisons, outcomes, and study design (PICOS).	3
**Methods**			
Protocol and registration	#5	Indicate if a review protocol exists, if and where it can be accessed (e.g., Web address) and, if available, provide registration information including the registration number.	3
Eligibility criteria	#6	Specify study characteristics (e.g., PICOS, length of follow-up) and report characteristics (e.g., years considered, language, publication status) used as criteria for eligibility, giving rational	3,4
Information sources	#7	Describe all information sources in the search (e.g., databases with dates of coverage, contact with study authors to identify additional studies) and date last searched.	3
Search	#8	Present full electronic search strategy for at least one database, including any limits used, such that it could be repeated.	3, Appendix 1
Study selection	#9	State the process for selecting studies (i.e., for screening, for determining eligibility, for inclusion in the systematic review, and, if applicable, for inclusion in the meta-analysis).	4, Figure 1

For peer review only

Data collection process	#10	Describe the method of data extraction from reports (e.g., piloted forms, independently by two reviewers) and any processes for obtaining and confirming data from investigators.	4
Data items	#11	List and define all variables for which data were sought (e.g., PICOS, funding sources), and any assumptions and simplifications made.	3
Risk of bias in individual studies	#12	Describe methods used for assessing risk of bias in individual studies (including specification of whether this was done at the study or outcome level, or both), and how this information is to be used in any data synthesis.	4
Summary measures	#13	State the principal summary measures (e.g., risk ratio, difference in means).	N/A
Planned methods of analyis	#14	Describe the methods of handling data and combining results of studies, if done, including measures of consistency (e.g., I2) for each meta-analysis.	4,5
Risk of bias across studies	#15	Specify any assessment of risk of bias that may affect the cumulative evidence (e.g., publication bias, selective reporting within studies).	4
Additional analyses	#16	Describe methods of additional analyses (e.g., sensitivity or subgroup analyses, meta-regression), if done, indicating which were pre-specified.	N/A

Results

For peer review only

Study selection	#17	Give numbers of studies screened, assessed for eligibility, and included in the review, with reasons for exclusions at each stage, ideally with a flow diagram.	Figure 1
Study characteristics	#18	For each study, present characteristics for which data were extracted (e.g., study size, PICOS, follow-up period) and provide the citation.	5,6,7,8,9,10 (Table 1)
Risk of bias within studies	#19	Present data on risk of bias of each study and, if available, any outcome-level assessment (see Item 12).	Appendix 2
Results of individual studies	#20	For all outcomes considered (benefits and harms), present, for each study: (a) simple summary data for each intervention group and (b) effect estimates and confidence intervals, ideally with a forest plot.	N/A
Synthesis of results	#21	Present the main results of the review. If meta-analyses are done, include for each, confidence intervals and measures of consistency.	11,12,13,14,15
Risk of bias across studies	#22	Present results of any assessment of risk of bias across studies (see Item 15).	10
Additional analysis	#23	Give results of additional analyses, if done (e.g., sensitivity or subgroup analyses, meta-regression [see Item 16]).	N/A

Discussion

Summary of Evidence	#24	Summarize the main findings, including the strength of evidence for each main outcome; consider their	16

For peer review only

		relevance to key groups (e.g., health care providers, users, and policy makers	
Limitations	#25	Discuss limitations at study and outcome level (e.g., risk of bias), and at review level (e.g., incomplete retrieval of identified research, reporting bias).	17
Conclusions	#26	Provide a general interpretation of the results in the context of other evidence, and implications for future research.	18

Funding

Funding	#27	Describe sources of funding or other support (e.g., supply of data) for the systematic review; role of funders for the systematic review.	1

Notes:

- 8: 3, appendix 1
- 9: 4, Figure 1
- 18: 5,6,7,8,9,10 (Table 1)
- 21: 11,12,13,14,15

The PRISMA checklist is distributed under the terms of the Creative Commons Attribution License CC-BY. This checklist was completed on 13/11/2019

For peer review only
